# Cardiac pericyte reprogramming by MEK inhibition promotes arteriologenesis and angiogenesis of the ischemic heart

**DOI:** 10.1172/JCI152308

**Published:** 2022-05-16

**Authors:** Elisa Avolio, Rajesh Katare, Anita C. Thomas, Andrea Caporali, Daryl Schwenke, Michele Carrabba, Marco Meloni, Massimo Caputo, Paolo Madeddu

**Affiliations:** 1 Bristol Medical School, Translational Health Sciences, and Bristol Heart Institute, University of Bristol, Bristol, United Kingdom.; 2 Department of Physiology, HeartOtago, School of Biomedical Sciences, University of Otago, Dunedin, New Zealand.; 3 University/BHF Centre for Cardiovascular Science, University of Edinburgh, Edinburgh, United Kingdom.

**Keywords:** Angiogenesis, Vascular Biology, Cardiovascular disease, Microcirculation, Molecular biology

## Abstract

Pericytes (PCs) are abundant yet remain the most enigmatic and ill-defined cell population in the heart. Here, we investigated whether PCs can be reprogrammed to aid neovascularization. Primary PCs from human and mouse hearts acquired cytoskeletal proteins typical of vascular smooth muscle cells (VSMCs) upon exclusion of EGF/bFGF, which signal through ERK1/2, or upon exposure to the MEK inhibitor PD0325901. Differentiated PCs became more proangiogenic, more responsive to vasoactive agents, and insensitive to chemoattractants. RNA sequencing revealed transcripts marking the PD0325901-induced transition into proangiogenic, stationary VSMC-like cells, including the unique expression of 2 angiogenesis-related markers, aquaporin 1 (AQP1) and cellular retinoic acid–binding protein 2 (CRABP2), which were further verified at the protein level. This enabled us to trace PCs during in vivo studies. In mice, implantation of Matrigel plugs containing human PCs plus PD0325901 promoted the formation of **α**SMA^+^ neovessels compared with PC only. Two-week oral administration of PD0325901 to mice increased the heart arteriolar density, total vascular area, arteriole coverage by PDGFR**β**^+^AQP1^+^CRABP2^+^ PCs, and myocardial perfusion. Short-duration PD0325901 treatment of mice after myocardial infarction enhanced the peri-infarct vascularization, reduced the scar, and improved systolic function. In conclusion, myocardial PCs have intrinsic plasticity that can be pharmacologically modulated to promote reparative vascularization of the ischemic heart.

## Graphical abstract

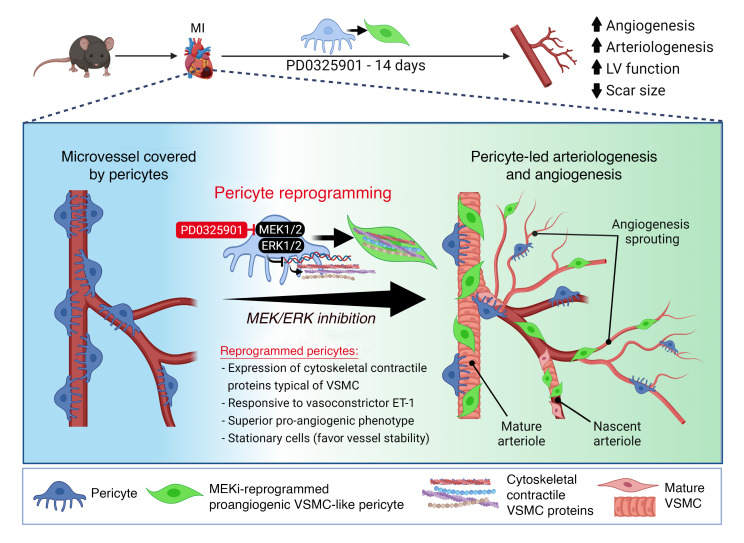

## Introduction

The outcome after myocardial infarction (MI) is tightly dependent on the proper growth of preexistent collateral arteries and the formation and maturation of capillaries into new arterioles through sprouting and mural cell coverage (1–3). Patients capable of developing good coronary circulation after an MI have a better outcome than patients with poor coronary circulation (3). Therefore, there is a tremendous interest in deploying new therapies capable of boosting the endogenous vascularization potential by reprogramming resident cardiac cells.

Pericytes (PCs) are mesoderm-derived cells that wrap around endothelial cells (ECs) in arterioles, capillaries, and venules. They share some antigenic markers with other stromal cells, such as myofibroblasts, but are supposed to play distinct functional roles in vascular stabilization, remodeling, and protracted contraction after ischemia-reperfusion (4–11). A lineage-tracing study showed that epicardial PCs are the ancestors of coronary vascular smooth muscle cells (VSMCs) in the developing murine heart (12). Nonetheless, the lack of unequivocal markers has so far precluded a full understanding of the PC plasticity in homeostasis and regeneration.

The present study aimed to determine whether induced phenotypic transition of myocardial PCs can aid heart neovascularization. First, we asked whether it would be possible to modulate myocardial PCs’ expressional and functional characteristics by removing selected growth factors (GFs) from the culture medium or inhibiting the downstream ERK1/2 signaling. Second, having demonstrated the PC commitment to a VSMC-like phenotype, we determined the underpinning molecular signature using whole-genome RNA sequencing (RNA-Seq). Third, we tested the proangiogenic effect of a MEK1/2 inhibitor (PD0325901) in in vivo models: (a) naive human PCs were embedded in Matrigel containing either PD0325901 or vehicle and injected subcutaneously in C57BL/6J mice and (b) in 2 randomized, controlled studies, PD0325901 was administered to intact or infarcted C57BL/6J mice. Results documented the capacity of cardiac PCs to transit to a contractile, proangiogenic phenotype in vitro and to participate in the neovascularization promoted in vivo by PD0325901.

## Results

### Human cardiac PC characterization.

**Figure 1:**
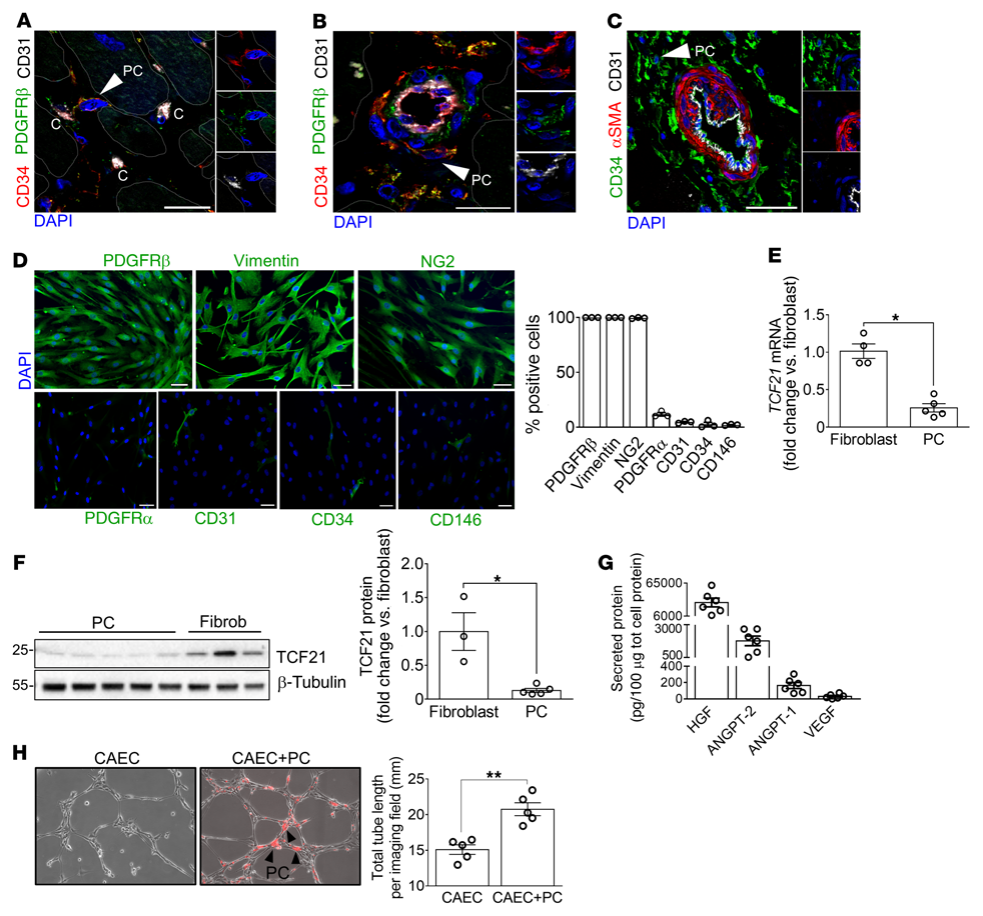
Human cardiac PC antigenic and functional characterization. (**A**–**C**) Confocal immunofluorescence images of human hearts. Arrows point to CD31^–^CD34^+^PDGFRβ^+^αSMA^–^ PCs around capillaries (indicated with “C”) and arterioles. Scale bars: 20 μm (**A** and **B**) and 100 μm (**C**). (**D**) Immunofluorescence images and bar graphs showing PC antigenic profile at passage 5 of culture. Scale bars: 50 μm. *n =* 3 patients’ PCs. Representative images are from 1 patient. (**E** and **F**) Expression of TCF21 in cardiac PCs and fibroblasts evaluated by RT-qPCR (**E**) and Western blotting (**F**). *n =* 4 fibroblasts in **E** (from 2 donors, assayed in independent experimental duplicates) and *n =* 3 fibroblast donors in **F**, *n =* 5 patients’ PCs. (**G**) Angiogenic factors secreted by cardiac PCs. Amounts of secreted factors throughout 48 hours were normalized against the total intracellular protein content. *n =* 6 patients’ PCs. (**H**) 2D-Matrigel assay with human coronary artery ECs (CAECs) in monoculture or coculture with cardiac PCs. PCs were labeled with dil (red fluorescent dye). Black arrowheads point to examples of PCs. *n =* 5 patients’ PCs, *n =* 1 CAEC. Representative images are from 1 patient’s PCs. All data are presented as individual values and mean ± SEM. *P < 0.05; **P < 0.01 by unpaired Mann-Whitney *U* test.

As previously reported in pediatric hearts (13), we identified CD31^–^CD34^+^PDGFRβ^+^α-smooth muscle actin^–^ (αSMA^–^) PCs around capillaries and within the adventitia of arteries in adult human hearts (Figure 1, A–C). CD31^–^CD34^+^ sorted PCs grew in culture, showing a spindle-shaped morphology and typical antigenic profile (Figure 1D and ref. 13). Compared with cardiac fibroblasts, PCs express remarkably lower PDGFRα and transcription factor 21 (*TCF21*) (Figure 1, D–F, and Supplemental Figure 1A; supplemental material available online with this article; https://doi.org/10.1172/JCI152308DS1), thus confirming the difference between the 2 populations (14–16). Cardiac PCs secrete the angiogenic factors HGF, angiopoietin-2 (ANGPT-2), ANGPT-1, and VEGF (Figure 1G), with their expression levels being significantly different from those of control coronary artery ECs (CAECs) and cardiac fibroblasts (Supplemental Figure 1B). Finally, PCs did not form networks on Matrigel but, when cocultured with CAECs, they promoted the formation of longer tubular networks, establishing mutual contacts with CAECs at the branch and intersection levels (Figure 1H and Supplemental Figure 1C).

### EGF and bFGF restrain cardiac PCs from differentiation into VSMC-like cells.

**Figure 2:**
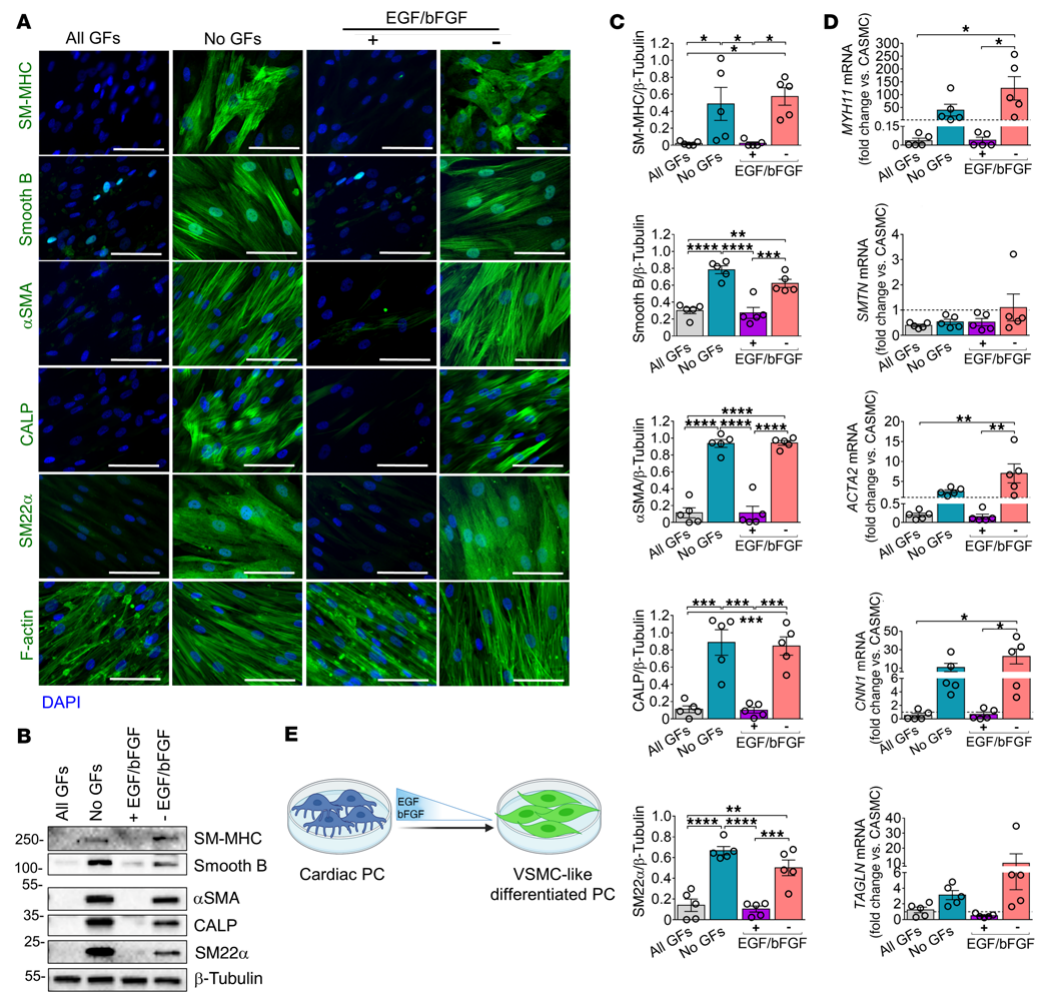
EGF and bFGF control human cardiac PC differentiation into VSMC-like cells. (**A**) Immunofluorescence images showing expression of cytoskeletal proteins by naive and differentiated PCs when cultured with different GF combinations for 10 days. All GFs: VEGF, IGF-1, EGF, bFGF. No GFs: depletion of all GFs. – EGF/bFGF: only VEGF and IGF1 were added to the culture medium. + EGF/bFGF: only EGF and bFGF. Scale bars: 50 μm. Representative images are from 1 patient. (**B** and **C**) Western blotting analysis of VSMC markers in naive and differentiated PCs. Representative blots are from 1 patient. Graphs show blot densitometry for all patients. (**D**) Transcriptional analysis of contractile SM genes in naive and differentiated PCs. mRNA data are expressed as fold change versus coronary artery SMCs (CASMCs) used as reference population (dashed line at *y* = 1). For all analyses, *n =* 5 patients’ PCs. Data are presented as individual values and mean ± SEM. *P < 0.05; **P < 0.01; ***P < 0.001; ****P < 0.0001 by ordinary 2-way ANOVA followed by Tukey’s multiple comparisons test. (**E**) Cartoon illustrating the role of EGF and bFGF in regulating the PC phenotype.

Like the earlier studied pediatric patients’ PCs (13), we found that the optimal medium to expand adult cardiac PCs contains human recombinant EGF, basic FGF (bFGF), IGF-1, and VEGF. In this medium, cardiac PCs remained viable for several passages and retained their original phenotype (“All GFs”; Figure 2, A–D). However, when testing different media, we discovered that PC culture in a medium depleted of all GFs induced a phenotypic change toward the VSMC phenotype. This intriguing observation prompted us to investigate entry points of PC plasticity and regenerative potential. As shown in Figure 2, A–D, expansion in medium depleted of GF for 10 days (“No GFs”) induced PCs to acquire intermediate and late-stage VSMC proteins: smooth muscle (SM) protein 22-α (SM22α, gene *TAGLN*), SM calponin (CALP, *CNN1*), αSMA (*ACTA2*), smoothelin B (*SMTN*), and SM myosin heavy chain (SM-MHC, *MYH11*) (Supplemental Table 1). Adding EGF and bFGF alone and, even more so in combination, to the basal medium prevented the expression of SM markers (“+EGF/bFGF”; Figure 2, A–E, and Supplemental Figure 2). Conversely, VEGF and IGF1 did not halt the cell differentiation (“–EGF/bFGF”; Figure 2, A–D). Both PC and PC-derived VSMC-like cells express neuron-glial antigen 2 (NG2, *CSPG4*) and PDGFRβ (*PDGFRB*) (Supplemental Figure 3), antigens shared by mural cells. Likewise, human coronary artery VSMCs (CASMCs) upregulated contractile markers in response to GF depletion (Supplemental Figure 4).

### Functional characterization of differentiated cardiac PCs.

**Figure 3:**
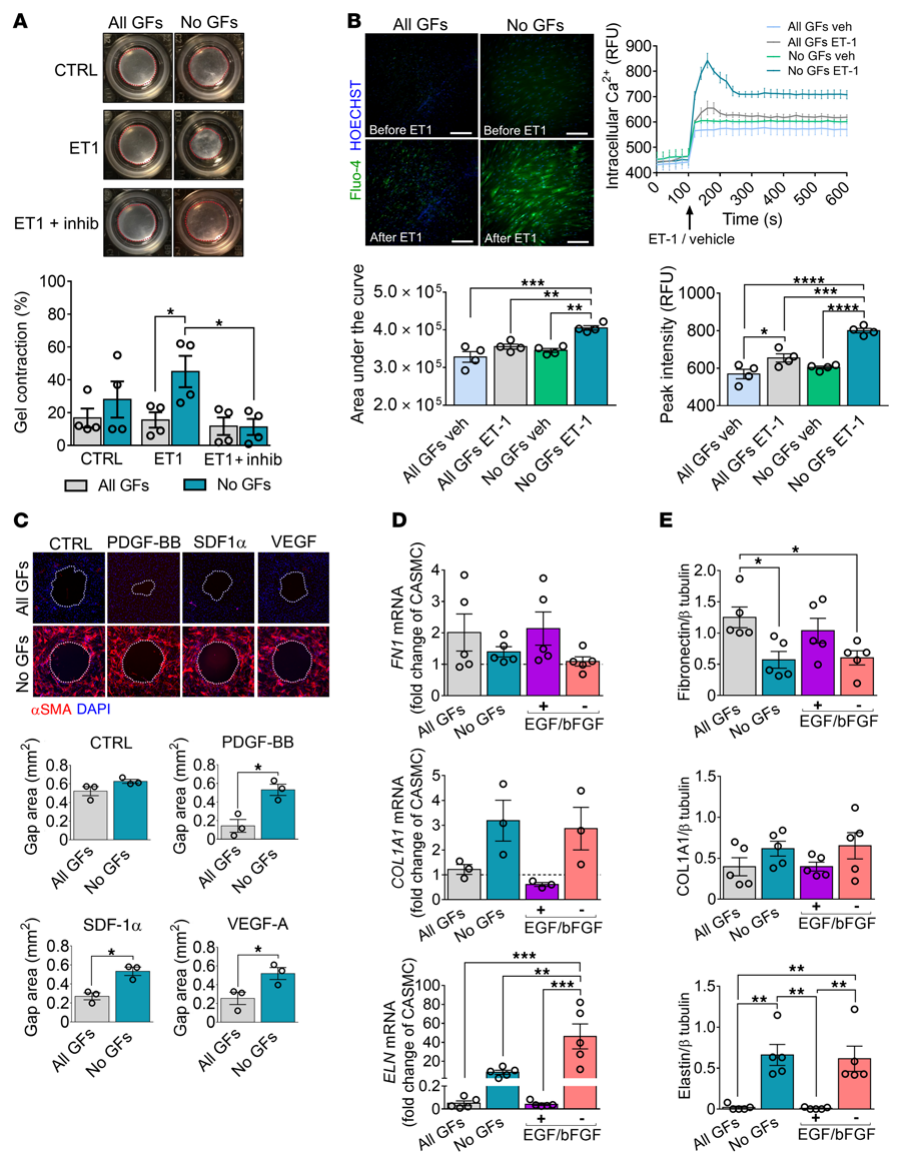
Human cardiac PCs differentiated without GFs display functional properties of contractile VSMCs. PCs were cultured with different GF combinations for 10 days and then used for functional assays. All GFs: VEGF, IGF-1, EGF, bFGF. No GFs: depletion of all GFs. – EGF/bFGF: only VEGF and IGF1. + EGF/bFGF: only EGF and bFGF. (**A**) Contraction assay. Cells were embedded in collagen gels, treated with a contraction inhibitor (inhib), and stimulated with endothelin-1 (ET-1). Bar graphs indicate the percentage of gel contraction after 24 hours. *n =* 4 patients’ PCs. Representative images are from 1 patient. (**B**) Fluo-4 calcium assay. Cells were loaded with the Fluo-4 dye and stimulated with ET-1 or vehicle. The intracellular calcium flux was measured as relative fluorescence units (RFU, green). Scale bars: 50 μm. Curves summarize *n =* 4 patients’ PCs (mean ± SEM are reported for each time point). Bar graphs show the quantification of the area under the curve and the peak fluorescence intensity. Representative images are from 1 patient. (**C**) Gap closure migration assay. Migration time was 24 hours. The absence of stimuli served as control (CTRL). Bar graphs show the final area of the gap. *n =* 3 patients’ PCs. Representative images are from 1 patient. (**D** and **E**) Expression of extracellular matrix proteins and transcripts. mRNA data are expressed as a fold change versus coronary artery SMCs (CASMCs) used as reference population (dashed line at *y* = 1). *n =* 3 to 5 patients’ PCs. All data are individual values and mean ± SEM. *P < 0.05; **P < 0.01; ***P < 0.001; ****P < 0.0001 by unpaired Kruskal-Wallis followed by Dunn’s multiple comparisons test to compare the 3 treatment groups (CTRL, ET-1, ET-1 + inhib) per experimental condition, and unpaired Mann-Whitney *U* test to compare the 2 experimental groups (All GFs and No GFs) per treatment (**A**); ordinary 2-way ANOVA followed by Tukey’s multiple comparisons test (**B**, **D**, and **E**); or unpaired Mann-Whitney *U* test (**C**).

Following GF deprivation, differentiated PCs (DPCs) became more responsive to endothelin-1 (ET-1) in a contraction assay, and this response was prevented by a myosin ATPase inhibitor (Figure 3A). They also presented a greater intracellular calcium mobilization in response to ET-1 (Figure 3B). The peak calcium fluorescence increased by 14% in ET-1–stimulated naive PCs compared with vehicle-treated controls, and this response was further amplified in DPCs (+33% vs. vehicle) (Figure 3B). In a wound closure assay, DPCs did not respond to chemoattractant stimuli that induced migration of naive PCs (Figure 3C). Moreover, PC differentiation impacted the transcription of extracellular matrix proteins; DPCs produced lower amounts of fibronectin (FN1) but more elastin (ELN) (Figure 3, D and E). Production of collagen 1, in contrast, did not change with treatment (Figure 3, D and E). Like DPCs, differentiated CASMCs showed limited migration, produced less FN1, and secreted ELN (Supplemental Figure 5).

These results indicate that GF-depleted PCs acquire antigenic and typical functional features of contractile VSMCs.

### PC differentiation is dependent on ERK1/2.

**Figure 4:**
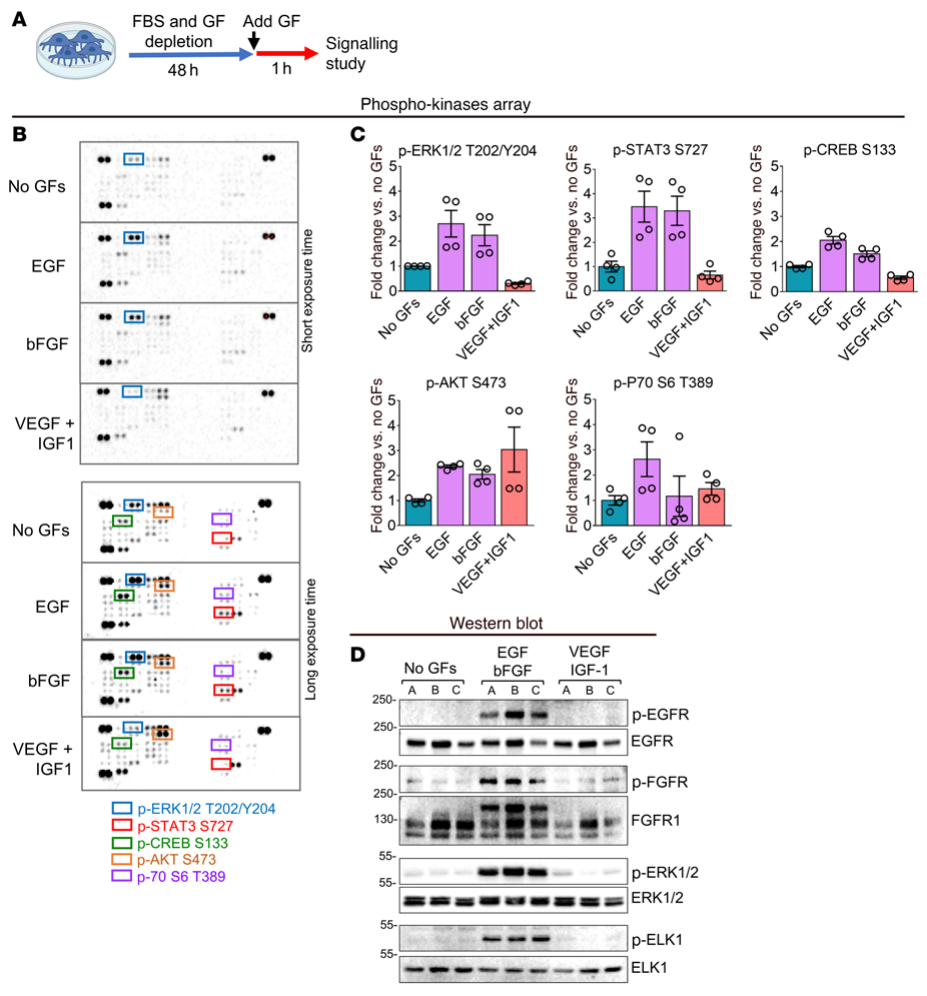
Signaling studies in cardiac PCs. (**A**–**C**) Phospho-kinase array. For a quick screening of the intracellular signaling activated by EGF and bFGF in cardiac PCs, we performed a human phospho-kinase protein array (*n =* 2 patients’ PCs). The array allowed the detection of the phosphorylation of 43 kinases. (**A**) Experimental protocol. (**B**) Membranes representative of *n =* 1 PC. (**C**) Targets whose phosphorylation was induced by EGF and bFGF. Densitometry graphs show the quantification of all replicate spots from *n =* 2 patients’ PCs (2 spots each). Data are presented as individual values and mean ± SEM. No statistical tests were applied. (**D**) Western blot indicating the activation of EGFR/FGFR/ERK1/-2/ELK1 signaling by EGF and bFGF in cardiac PCs. *n =* 3 patients’ PCs, indicated by A, B, and C.

**Figure 5:**
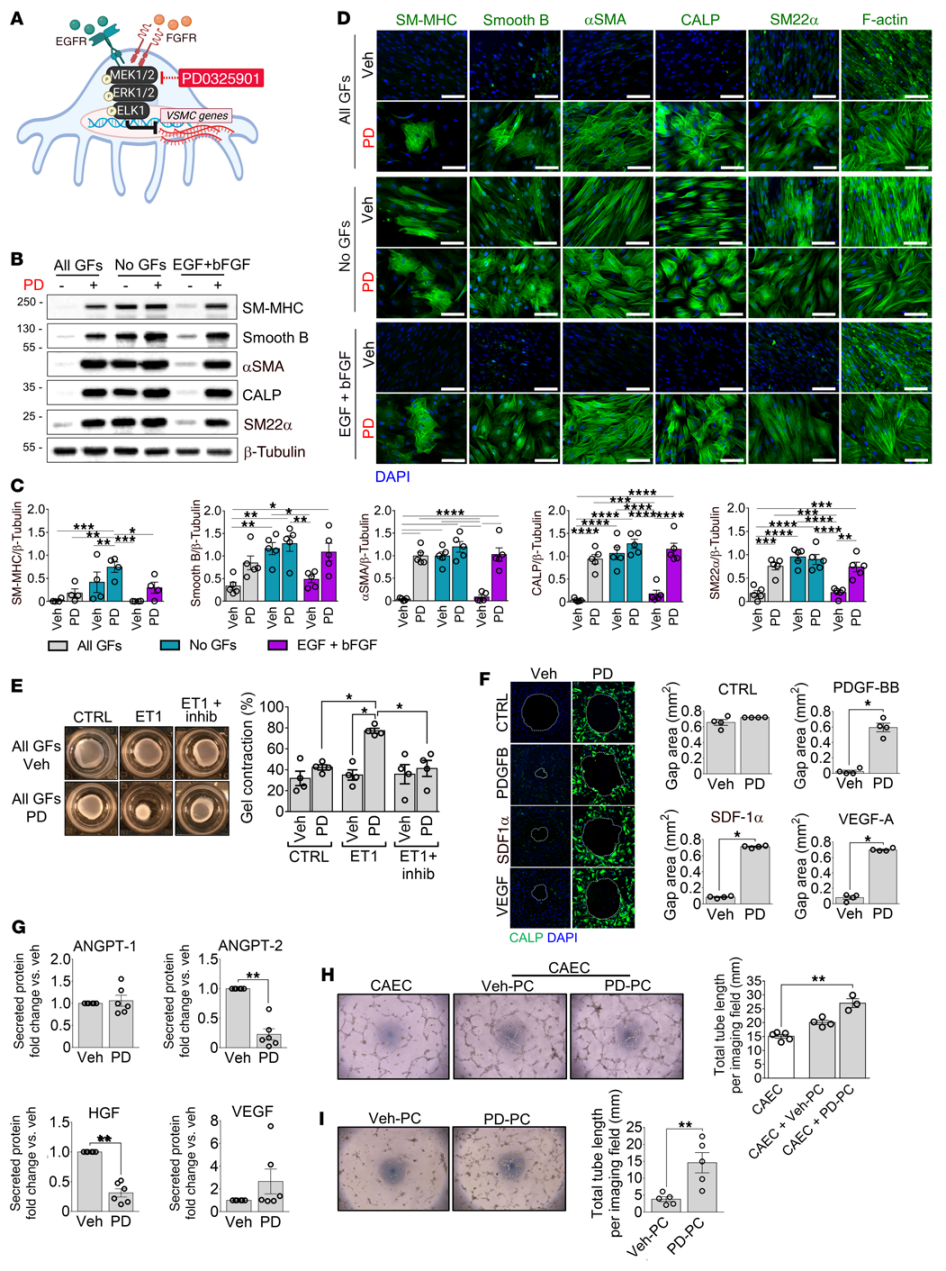
Inhibition of the MEK1/-2/ERK1/-2 signaling induces the switch from human cardiac PCs into VSMC-like cells in vitro. (**A**) Schematic showing EGF and bFGF signaling in cardiac PCs and the MEK1/2 inhibitor employed. (**B**–**I**) PCs were cultured for 10 days with different media as indicated, in the presence of PD0325901 (PD, 250 nM) or DMSO (Veh), before using them for the functional assays. (**B** and **C**) Analyses of protein expression using Western blotting. Representative blots are from 1 patient, and graphs show blot densitometry for *n =* 5 patients’ PCs. (**D**) Representative immunofluorescence images of PCs from 1 patient show contractile VSMC proteins and cytoskeletal F-actin expression (green). Scale bars: 50 μm. *n =* 5 patients’ PCs. (**E**) Contraction assay. Cells were embedded in collagen gels, treated with a contraction inhibitor (inhib), and stimulated with endothelin-1 (ET-1). Bar graphs indicate the percentage of gel contraction after 24 hours. (**F**) Gap closure migration assay. Migration time was 24 hours. Bar graphs report the area of the final gap. *n =* 4 patients’ PCs (**E** and **F**). Representative images are from 1 patient. SDF-1α, stromal cell–derived factor 1α. (**G**) Secreted angiogenic factors. *n =* 6 patients’ PCs. (**H**) 2D-Matrigel assay with human coronary artery ECs (CAECs) and PCs. CAECs were used in monoculture or cocultures with either Veh-PC or PD0325901-treated PC (PD-PC). *n =* 3 or 4 patients’ PCs. *n =* 1 CAEC (assayed 5 times). (**I**) 2D-Matrigel assay with PCs alone. *n =* 5 patients’ PCs. All data are plotted as individual values and mean ± SEM. *P < 0.05; **P < 0.01; ***P < 0.001; ****P < 0.0001 by ordinary 2-way ANOVA followed by Tukey’s multiple comparisons test (**C**); unpaired Kruskal-Wallis followed by Dunn’s multiple comparisons test to compare the 3 treatment groups (CTRL, ET-1, ET-1 + inhib) per experimental condition, and unpaired Mann-Whitney *U* test to compare the 2 experimental groups (All GFs Veh and All GFs PD) per treatment (**E**); unpaired Mann-Whitney *U* test (**F***,* **G***,* and **I**); or unpaired Kruskal-Wallis followed by Dunn’s multiple comparisons test (**H**).

An initial screening analysis using a phospho-kinase array showed that EGF and bFGF but not VEGF and IGF1 activated ERK1/2 and its downstream targets STAT3 and cAMP response element–binding protein (CREB) in PCs (Figure 4, A–C). Instead, all GFs activated the AKT pathway (Figure 4, A–C). The phosphorylation of other kinases included in the array was not affected by the addition of EGF or bFGF (data not shown). Phosphorylation/activation of E26 transformation-specific (ETS) like-1 protein (ELK1) by ERK1/2 reportedly prevents SM gene transcription (17, 18). In our study, Western blotting confirmed that EGF and bFGF induce the phosphorylation/activation of the EGFR/FGFR/ERK1/-2/ELK1 axis in cardiac PCs (Figure 4, A and D). The activation of ERK1/2 is induced by MEK1/2 phosphorylation of both Thr and Tyr residues in ERK1/2’s activation loop (19). Therefore, we interrogated the possibility of inducing PC differentiation through pharmacological inhibition of MEK1/2 activity (Figure 5A). Dose-response studies confirmed that the MEK inhibitor (MEKi) PD0325901, a small molecule that binds to an allosteric site in the MEK activation loop, prevented downstream ERK1/2 phosphorylation for at least 48 hours without affecting PC viability when used at a 250 nM concentration (Supplemental Figure 6). Higher concentrations, especially 1 μM and higher, were associated with decreased cell survival. Therefore, we used the 250 nM dose throughout subsequent in vitro experiments.

### Exposure to PD0325901 recapitulates the GF removal–induced differentiation of cardiac PCs into VSMC-like cells.

Culture of cardiac PCs with the medium supplemented with GFs plus PD0325901 for 10 days induced the cells to acquire the expression of cytoskeletal proteins that characterize the VSMC-like phenotype of GF-depleted DPCs (Figure 5, B–D). Moreover, PCs differentiated with all GFs plus PD0325901 contracted in response to ET-1 (Figure 5E) and became unresponsive to promigratory stimuli (Figure 5F). Furthermore, PD0325901-treated PCs secreted lower amounts of ANGPT-2 and HGF than PCs treated with DMSO vehicle (Figure 5G). In addition, PD0325901-treated PCs outperformed Veh-treated PCs in enhancing CAEC network formation on Matrigel (Figure 5H). PD0325901-treated PCs were also better than Veh-treated PCs in forming networks when seeded alone on Matrigel (Figure 5I). Instead, monocultures of CAECs treated with PD0325901 produced fewer networks than untreated cells, suggesting PD0325901 exerts differential effects on PCs and CAECs and requires the presence of both cell types to encourage in vitro network formation (Supplemental Figure 7). Additionally, either the removal of GFs or treatment with PD0325901, and even more so their combination, reduced PC proliferation (Supplemental Figure 8). To discern the contribution of the cell cycle arrest to PC differentiation, we compared the effects of PD0325901 and ribociclib, a selective inhibitor of the cyclin D1–cyclin-dependent kinase 4/6 (CDK4/6) complexes that prevents the progression from the G1 to the S phase of the cell cycle. As shown in Supplemental Figure 9, ribociclib strongly inhibited PC proliferation but, in contrast to PD0325901, was unable to induce PC differentiation. Finally, the PI3K inhibitor LY294002 failed to induce PC differentiation (Supplemental Figure 10).

PD0325901 upregulated contractile markers and inhibited proliferation also in control CASMCs (Supplemental Figure 11).

Last, we checked whether PD0325901 affected another relevant cardiac cell population, namely, fibroblasts. As shown in Supplemental Figure 12, A and B, MEKi treatment caused a significant upregulation of the αSMA protein while downregulating FN1 and vimentin. Furthermore, a wound closure assay indicated that fibroblasts preconditioned with PD0325901 quickly migrate in response to stimulation with FBS, while untreated fibroblasts do not (Supplemental Figure 12C), a behavior opposite to that of PD0325901-treated PCs.

### Global RNA analysis of PC differentiation.

**Figure 6:**
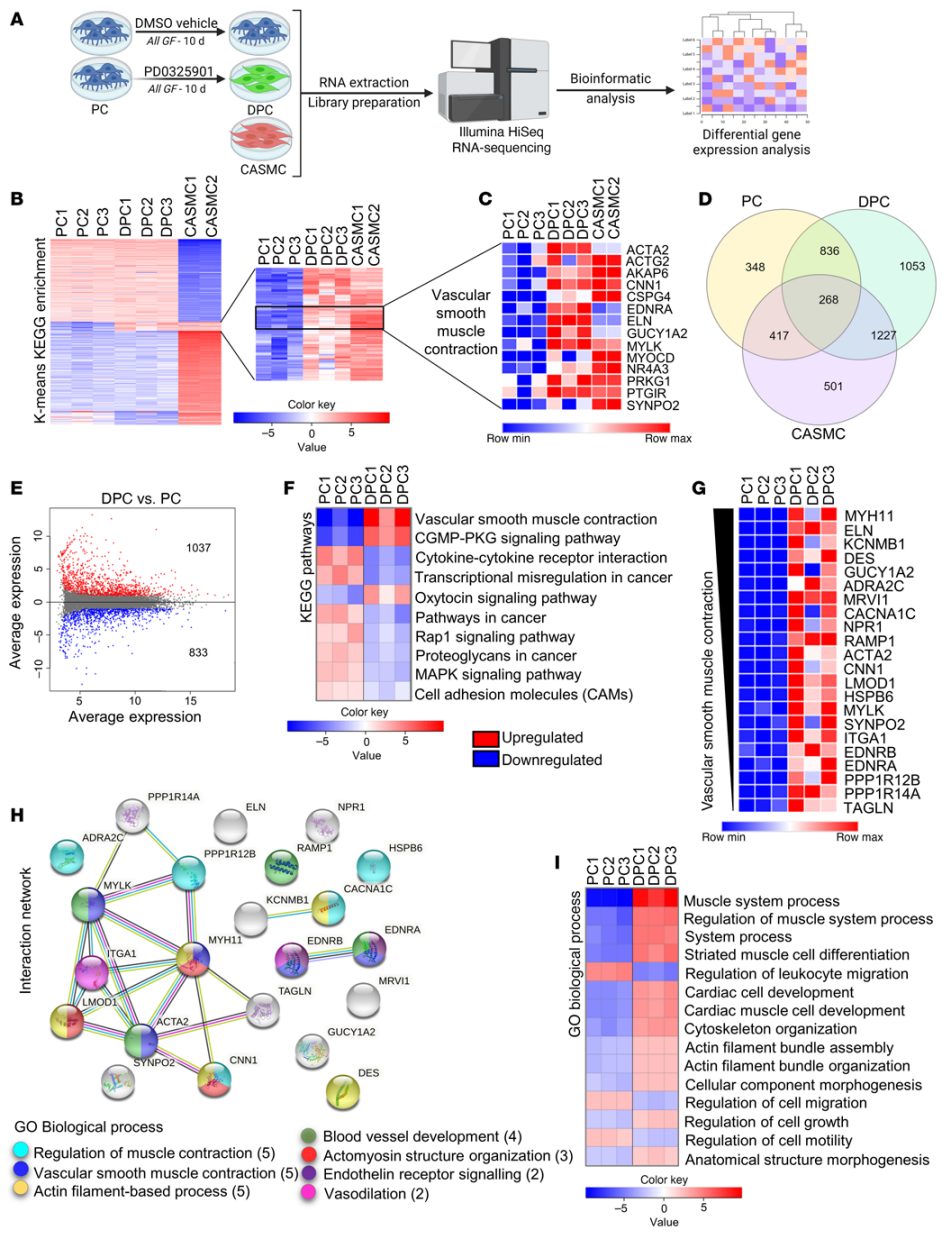
Next-generation RNA-Seq analysis of naive and differentiated human cardiac PCs. (**A**) Experimental design. RNA-Seq was performed in vehicle-treated PCs (*n =* 3 patients), PD0325901-differentiated PCs (DPC, *n =* 3 patients), and human coronary artery SMCs (CASMCs) (*n =* 2 donors). (**B**) K-means KEGG analysis of genes differentially expressed in the 3 cell populations. (**C**) List of most predominant genes associated with the pathway “Vascular smooth muscle contraction.” (**D**) The Venn diagram shows the number of transcripts expressed uniquely or shared by the 3 cell populations. (**E**) MA plot showing genes differentially expressed in DPCs versus naive PCs. (**F**) List of most regulated KEGG pathways in DPCs versus PCs. (**G**) Significant differentially expressed genes (DEGs) associated with “Vascular smooth muscle contraction.” (**H**) STRING protein-protein interaction analysis of genes in **F**, and emerging Gene Ontology (GO) Biological Process. (**I**) Main pathways resulting from the GO Biological Process analysis of DPCs versus PCs. Genes in the heatmap in **G** are ranked by log_2_FC. For **E**–**I**, adjusted *P* value < 0.05.

Next, to gather a more comprehensive view of the changes induced by PD0325901, we performed a whole-transcriptome analysis of naive PCs and DPCs. CASMCs were used as internal control (Figure 6A). As shown in Figure 6B, a cluster of genes was upregulated in both PD0325901-reprogrammed DPCs and CASMCs compared with naive PCs. Zooming into this cluster unveiled several genes encoding contractile proteins (Figure 6C). Moreover, the number of genes coexpressed by DPCs and CASMCs was 3-fold higher than that shared by naive PCs and CASMCs (Figure 6D). Supplemental Table 2 reports the 30 most differentially expressed genes (DEGs) in DPCs versus CASMCs.

The contrast between DPCs and PCs revealed 1,870 DEGs (FDR < 0.05 and absolute log_2_ fold change [log_2_FC] > 1), of which 1,037 were upregulated and 833 were downregulated (Figure 6E). The KEGG pathway “Vascular smooth muscle contraction” showed several genes upregulated in DPCs (Figure 6, F and G, and Supplemental Table 3; log_2_FC from +1.8 to +12.7). These genes were further analyzed in a STRING network, which showed that 13 proteins encoded by those genes have a strong biological connection (high confidence interaction score of 0.7 and protein-protein interaction [PPI] enrichment *P* value < 1 × 10^–16^) (Figure 6H). The main biological processes encompassed “Regulation of muscle contraction,” “Vascular smooth muscle contractile function,” and “Actomyosin structure organization.” As expected, the biological processes “Cytokine-cytokine receptor interaction,” “MAPK signaling,” and “Cell motility and migration” were downregulated in DPCs (Figure 6, F and I). A schematic view of the 2 main regulated pathways is further illustrated in Supplemental Figure 13, A and B. Moreover, RNA-Seq documented the significant downregulation of cyclin D1 transcripts (*CCND1*) in DPCs (log_2_FC = –1.77, *P* = 0.0000481; Supplemental Figure 13C).

**Figure 7:**
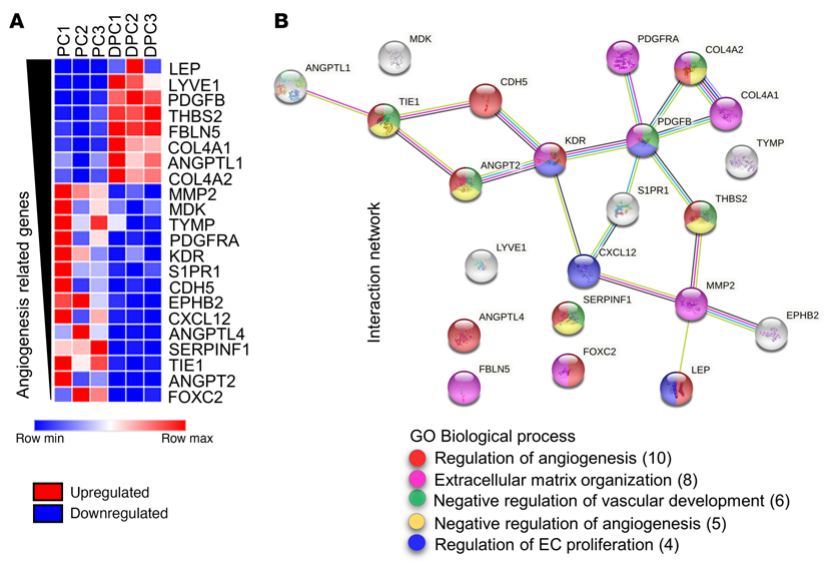
Next-generation RNA-Seq analysis of angiogenesis-related genes in naive and differentiated human cardiac PCs. (**A**) Analysis of angiogenesis-related DEGs in DPCs versus PCs. (**B**) STRING network analysis of angiogenesis-related genes and emerging GO Biological Process terms. Genes in the heatmap in **A** are ranked by log_2_FC. Absolute log_2_FC > 1.5. Adjusted *P* value < 0.05.

Last, we examined angiogenesis-related genes. Twenty-two genes were differentially expressed between DPCs and PCs (cutoff absolute log_2_FC > 1.5) (Figure 7A and Supplemental Table 4). A STRING analysis showed that 15 genes are biologically connected, with a confidence interaction score of 0.7 and PPI enrichment *P* value less than 1 × 10^–16^ (Figure 7B). Among downregulated genes, we found *ANGPT2* (encoding ANGPT-2), *TIE1* (tyrosine-protein kinase receptor tie-1), and *SERPINF1* (serpin family F member 1), all negative regulators of angiogenesis. Conversely, 2 factors secreted by PCs and enhancers of angiogenesis were the most upregulated (*LEP* [leptin] and *PDGFB*). Protein changes in secreted ANGPT-2, SERPINF1, and LEP in the cell-conditioned medium were validated using ELISA (Supplemental Figure 13D).

Altogether, these findings indicate that DPCs share transcriptional similarities with CASMCs and acquire a proangiogenic signature.

### Transcriptomics reveals markers unique to DPCs.

**Figure 8:**
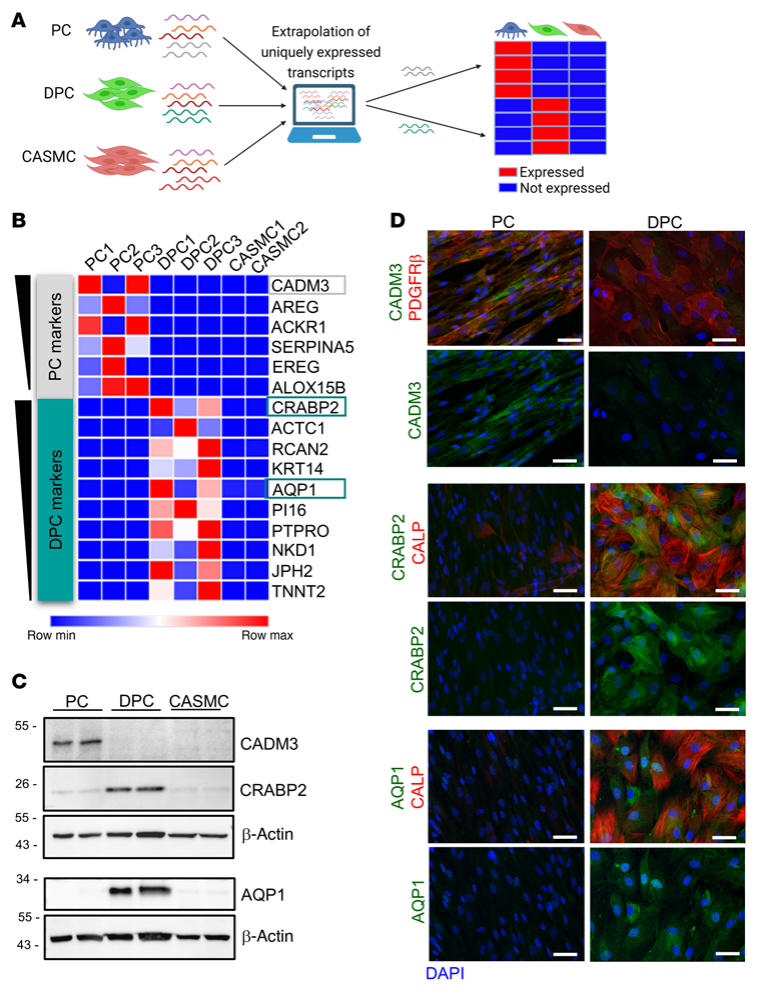
Discovery of unique antigens identifying naive PCs and VSMC-like differentiated PCs (DPCs). (**A**) Schematic illustrating the experimental design. We compared the RNA-Seq results for PCs, PD0325901-differentiated PCs (DPCs), and control human coronary artery SMCs (CASMCs) to identify transcripts uniquely expressed by PCs and DPCs. (**B**) List of top genes that emerged during the analysis. Genes in the heatmap are ranked for average transcripts per million (TPM) expression in the positive population. (**C** and **D**) Three transcripts were validated at the protein level using Western blotting (**C**) and immunocytochemistry (**D**) in human PCs (*n =* 2 patients, same patients’ cells used for the RNA-Seq). Scale bars: 50 μm. Representative immunofluorescence images of PCs are from 1 patient. CADM3, cell adhesion molecule 3; CRABP2, cellular retinoic acid–binding protein 2; AQP1, aquaporin 1. The antigens employed for histology were selected according to the following criteria: (a) high identity between the human and mouse proteins to allow matching data from studies in the 2 species, (b) intracellular or membrane marker for precise localization in PCs in situ (exclusion of soluble factors), and (c) suitability for microscopy imaging.

Next, we interrogated the RNA-Seq for transcripts uniquely expressed by PCs or DPCs versus CASMCs (Figure 8, A and B, Supplemental Figure 14, and Supplemental Table 5). Among the top genes uniquely expressed by each PC population, we further selected the hits that shared the highest identity between the human and mouse proteins (to allow matching data from studies in the 2 species), and that were suitable for histological identification. Unique protein expression was confirmed using Western blotting (Figure 8C) and immunocytochemistry (Figure 8D). This analysis unveiled cell adhesion molecule 3 (*CADM3*) (DPCs vs. PCs log_2_FC = –7.78, *P* = 1.8 × 10^–6^) as a marker of naive human PCs. In addition, the expression of the angiogenesis-related cellular retinoic acid–binding protein 2 (CRABP2) (DPCs vs. PCs log_2_FC = +7.52, *P* = 5.77 × 10^–14^) and aquaporin 1 (AQP1) (DPCs vs. PCs log_2_FC = +6.96, *P* = 0.00035) allowed distinguishing DPCs from naive PCs and CASMCs. CRABP2 controls angiogenesis through modulation of retinoic acid transport from the cytosol to the nuclear retinoic acid receptors (20), while AQP1 facilitates EC migration by a mechanism that involves water transport across angiogenic lamellipodia (21).

### PD0325901 promotes PC differentiation and neovascularization in an in vivo Matrigel plug assay.

**Figure 9:**
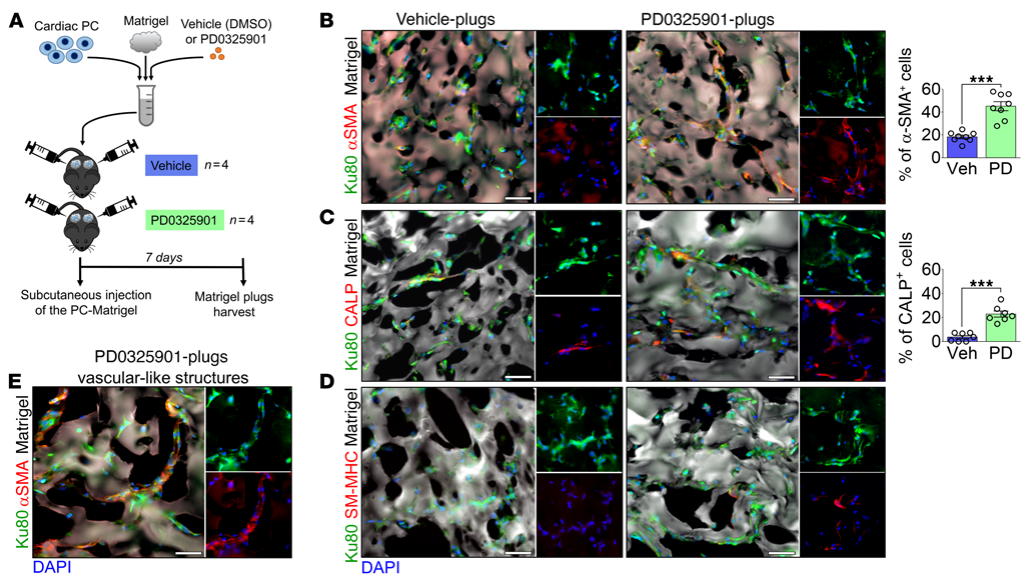
Inhibition of MEK1/-2/ERK1/-2 signaling induces the differentiation of human cardiac PCs into VSMC-like cells upon transplantation in vivo. (**A**) Experimental protocol of the in vivo Matrigel plug assay. (**B**–**D**) Immunofluorescence images of vehicle- and PD0325901-Matrigel plugs show that human cells express VSMC markers. Human PCs embedded in the Matrigel plugs were identified using the human Ku80 antigen. Bar graphs show the percentage of human PCs expressing αSMA and CALP. *n =* 7 or 8 plugs (from 4 mice). (**E**) Immunofluorescence image documenting the presence of αSMA^+^ vascular-like structures within the PD0325901-Matrigel plugs. Veh, vehicle; PD, PD0325901. Data are plotted as individual values and mean ± SEM. ***P < 0.001 by Mann-Whitney *U* test. Scale bars: 50 μm.

We then conducted in vivo studies to investigate the implications of cardiac PC reprogramming for tissue remodeling and repair. Naive human PCs were embedded in Matrigel containing either PD0325901 or DMSO vehicle and injected subcutaneously into C57BL/6J mice (Figure 9A). Plugs were harvested after 7 days, and the human Ku80-XRCC5 antigen was employed to recognize transplanted PCs (Figure 9, B–E, and Supplemental Figure 15A). Matrigel was identified using a secondary anti-mouse antibody (Supplemental Figure 15B). PD0325901 increased the fraction of spindle-shaped PCs that stained positive for αSMA and CALP within the Matrigel (Figure 9, B and C). Moreover, in the PD0325901 group, we identified occasional SM-MHC–positive cells (Figure 9D). Intriguingly, PC-covered tubular-like structures could be recognized only in PD0325901 plugs (Figure 9E). The influx of immune/inflammatory CD45^+^ cells was similar in the 2 groups, indicating that host immune response was irrelevant for differences regarding implanted cells (Supplemental Figure 15C).

### PD0325901 promotes myocardial arteriologenesis in healthy mice.

As shown initially in human hearts, we detected CD31^–^CD34^+^PDGFRβ^+^αSMA^–^ cells around arterioles and capillaries in the mouse heart (Supplemental Figure 16, A and B). We also confirmed that cultured murine PCs share a similar phenotype with human PCs (Supplemental Figure 16, C and D). Furthermore, treatment of murine PCs with 250 nM PD0325901 increased the expression of SM proteins while halting cell proliferation (Supplemental Figure 16, E–G).

**Figure 10:**
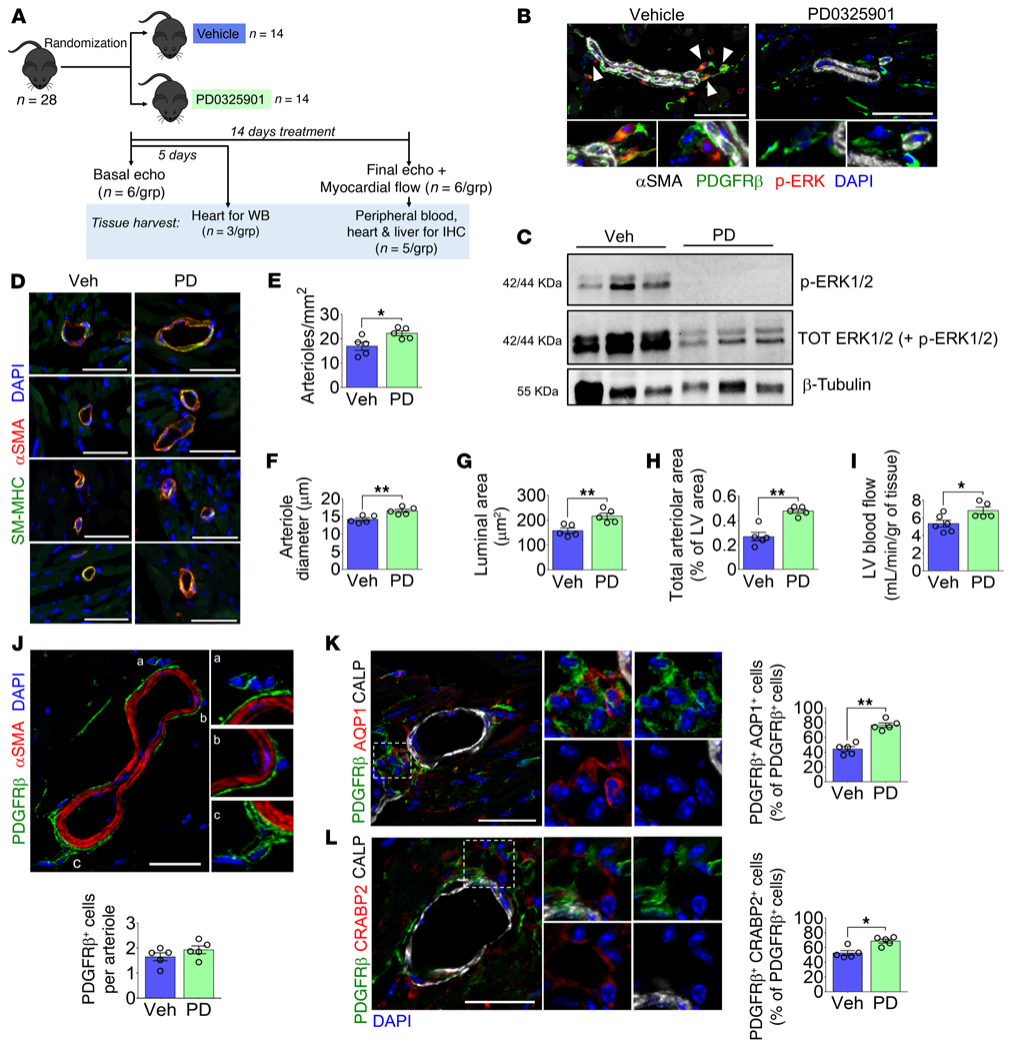
A 2-week treatment with PD0325901 induces arteriologenesis and improves perfusion of the healthy mouse heart. (**A**) Cartoon summarizing the experimental design. Mice were given the MEKi (10 mg/kg/d) or DMSO vehicle orally for 5 or 14 days. The drug was embedded in flavored jelly and eaten spontaneously by animals. All analyses were performed after 14 days, excluding the Western blot (WB) on heart samples done after 5 days. (**B**) Staining for p-ERK in PDGFRβ^+^ perivascular cells in the mouse hearts. Arrowheads point to p-ERK^+^ PCs in the vehicle group. Scale bars: 50 μm. *n =* 5 mice. (**C**) Western blot for p-ERK and total ERK using heart protein lysates confirmed the drug efficacy. *n =* 3 mice. (**D**) Immunofluorescence images showing examples of arterioles expressing αSMA and SM-MHC in vehicle- and PD-treated hearts. Scale bars: 50 μm. (**E**) Analysis of arteriole density in the left ventricle (LV). (**F**) Measurement of arterioles’ diameter in the LV. (**G**) Mean arteriolar luminal area, calculated starting from the mean diameter. (**H**) The total arteriolar area in the LV is expressed as a percentage of the whole LV area. (**I**) LV blood flow. (**J**) Immunofluorescence image of PDGFRβ^+^ PCs around arterioles and quantification of the average PC per arteriole in the LV. Scale bar: 20 μm. (**K** and **L**) Immunofluorescence images and analysis of PDGFRβ^+^AQP1^+^CRABP2^+^ cells around small arterioles in the LV. Scale bars: 20 μm. Graphs show the percentage of perivascular PDGFRβ^+^ cells expressing AQP1 or CRABP2. In **D**–**L**, *n =* 5 mice. Veh, vehicle; PD, PD0325901. Data are reported as individual values and mean ± SEM. *P < 0.05, **P < 0.01 by unpaired Mann-Whitney *U* test.

A controlled, randomized study was then conducted in C57BL/6J mice receiving PD0325901 at 10 mg/kg/d or vehicle (DMSO) orally for 14 days (Figure 10A). The absence of ERK1/2 phosphorylation in PD0325901 hearts, as demonstrated by immunostaining and Western blotting, confirmed the successful inhibition of MEK1/2 activity (Figure 10, B and C). In addition, we verified ERK1/2 inhibition in the liver (Supplemental Figure 17A).

Left ventricle (LV) function and dimension indices were similar between the 2 experimental groups (Supplemental Table 6). Likewise, histological examination of the LV showed no difference in cardiomyocyte cross-sectional area (Supplemental Figure 17B). Conversely, PD0325901-treated mice had a significant increase in the density and caliber of arterioles and an enlargement of the LV area occupied by arterioles (Figure 10, D–H). This is in line with an increased myocardial blood flow following PD0325901 treatment (Figure 10I). As shown in Figure 10J, the MEKi treatment did not affect the total number of PDGFRβ^+^ PCs surrounding arterioles. On the other hand, the treatment increased the relative abundance of PDGFRβ^+^AQP1^+^ and PDGFRβ^+^CRABP2^+^ cells, identifying bona fide DPCs (Figure 10, K and L).

The MEKi treatment had no effects on the heart and liver’s capillary density (Supplemental Figure 17, C and D), nor did it alter the population of cardiac fibroblasts and myofibroblasts (Supplemental Figure 17E).

Last, PD0325901 did not cause apoptosis in cardiomyocytes, vascular cells, and interstitial cells, nor did it increase plasmatic levels of cardiac troponin I (Supplemental Figure 17, F and G). These data indicate that PD0325901 safely and effectively enriched the myocardial vasculature with DPCs and improved perfusion without affecting cardiomyocytes and fibroblasts.

### PD0325901 improves LV function and revascularization in a mouse MI model.

**Figure 11:**
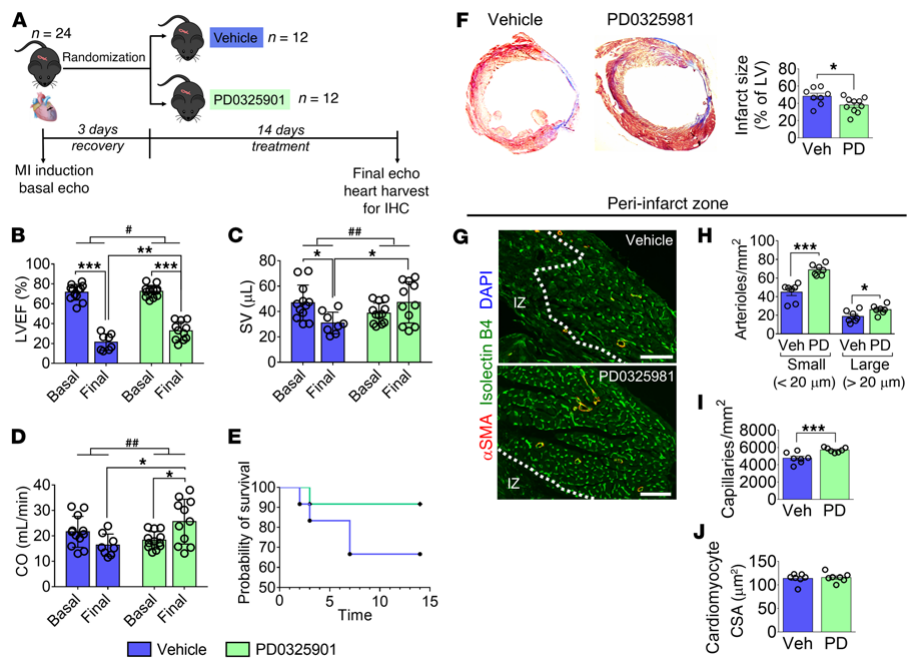
A 2-week treatment with PD0325901 improves left ventricular function and vascularization in a mouse MI model. (**A**) Cartoon summarizing the experimental design. Mice were given the MEKi (10 mg/kg/d) or DMSO vehicle orally for 14 days after MI induction. The drug was embedded in flavored jelly and eaten spontaneously by animals. (**B**–**D**) Graphs showing basal and final echocardiography indices. For vehicle, *n =* 12 mice basal and *n =* 8 final. For PD, *n =* 12 mice basal and *n =* 11 final. Individual values and mean ± SD. SV, stroke volume; CO, cardiac output. (**E**) Graph showing mouse survival. (**F**) Representative images showing the Azan-Mallory staining of the LV and bar graphs indicating the infarct size expressed as a percentage of the LV area. *n =* 8 mice for Veh, *n =* 10 mice for PD. (**G**) Representative immunofluorescence images showing arterioles (αSMA, red) and capillaries (isolectin B4, green) in the peri-infarct myocardium. The dashed line defines the infarct zone (IZ). Scale bars: 100 μm. (**H**–**J**) Graphs showing the quantification of arteriole (**H**) and capillary (**I**) densities and cardiomyocyte cross-sectional area (CSA) (**J**) in the LV. *n =* 7 mice. In **F**–**J**, individual values and mean ± SEM are shown. Veh, vehicle; PD, PD0325901. *P < 0.05, **P < 0.01, ***P < 0.001; ^#^*P <* 0.05, ^##^*P <* 0.01 in the comparison between changes (Δ). In **B**–**D**, 2-way ANOVA (mixed effects model with Sidak’s multiple comparison test) was performed considering that there were missing data in the 2 treatment groups due to premature death after MI. In addition, we compared the changes (Δ) from basal to final times in the 2 groups using an unpaired Student’s *t* test. In **F** and **H**–**J**, an unpaired Mann-Whitney *U* test was used.

Finally, a controlled, randomized study was conducted in C57BL/6J mice with MI. Three days after MI, mice were given PD0325901 or vehicle for 14 days (Figure 11A). Echocardiography confirmed MI induction, and baseline indices of LV function did not differ between groups.

At the endpoint, compared with vehicle, PD0325901-treated mice showed reduced LV dilatation (Supplemental Table 7) and improved contractile function, as indicated by higher LV ejection fraction (LVEF, decreased by 50% ± 3.8% from basal to final in the vehicle group, vs. –40% ± 3.0% in the PD0325901 group, *P* = 0.0455), stroke volume (–16 ± 5.2 μL from basal to final in the vehicle group, vs. +7.7 ± 5.8 μL in the PD0325901 group, *P* = 0.0092), and cardiac output (–5.6 ± 2.3 mL/min from basal to final in the vehicle group, vs. +7 ± 2.5 mL/min with PD0325901, *P* = 0.0025) (Figure 11, B–D). The survival rate did not differ between groups (Figure 11E). However, the composite endpoint of survival and LVEF above 30% was significantly better in the PD0325901 group (Fisher’s exact test *P =* 0.027) with an improved relative risk of 2.20 (95% CI = 1.23–4.80).

Histological analysis of the LV revealed smaller infarct scars in the PD0325901 group (Figure 11F). Moreover, in the peri-infarct zone, PD0325901 induced a significant increase in the small and large arteriole and capillary densities (Figure 11, G–I). Conversely, PD0325901 did not modify the vascularization in the remote myocardium (data not shown). Finally, the cardiomyocyte cross-sectional area was similar in the 2 experimental groups’ peri-infarct area (Figure 11J).

These data indicate that short-duration MEKi treatment benefits arteriologenesis and functional recovery of the infarcted heart.

## Discussion

We believe that this study provides a new mechanistic understanding of cardiac PC potential in vascular remodeling. In the heart, PCs may represent an incremental cellular reservoir for fueling arteriologenesis and recruiting/muscularizing newly formed capillaries. Importantly, we show that myocardial vascularization can be pharmacologically modulated in vivo using the selective MEKi PD0325901, although differences were observed between the normoperfused and the ischemic murine hearts. In the former, PD0325901 administration induced an increase in arterioles without affecting capillary density, whereas, when started at the early recovery stage from acute nonreperfused MI, the inhibitor potentiated arterioles selectively within the peri-infarct zone and incited capillarization. Differences in the temporal and spatial expression of GFs and phosphorylation/activation of p38 MAPK and ERK1/2 have been reported after an MI (22–24). These differences may account for the differential effect of PD0325901 on neovascularization in the remote and peri-infarct areas. Although further investigation is needed, our findings raise the intriguing possibility of manipulating mural cells to generate a robust microvasculature in the adult heart.

Environmental factors, including GFs that signal through ERK1/2 and p38 MAPK, can reportedly influence the phenotype and behavior of VSMCs in vitro and in vivo (25–29). In addition, PD0325901 was previously used to induce human pluripotent stem cell differentiation into the SMC lineage (30). Here, we show that both GF depletion and PD0325901 instigate naive PCs to acquire a contractile phenotype and functional properties instrumental to repair and regeneration. In vitro, DPCs became stationary in migration assays. This property is important for establishing a tighter interaction with ECs and stabilizing the nascent vasculature. We also observed that PD0325901-treated DPCs became able to assemble in vascular-like tubes in an in vitro angiogenesis assay and formed more complex tubular networks in cooperation with ECs. Interestingly, CAECs preconditioned with PD0325901 showed a decreased angiogenic activity in the absence of PCs in vitro, suggesting that both cell types are required to achieve the benefit of the drug treatment. The transcriptomic analysis further revealed that DPCs have a potent proangiogenic profile consequential to the downregulation of disruptors of angiogenesis, namely *ANGPT2*, *TIE1*, and *SERPINF1*, and the upregulation of the proangiogenic factor *LEP*. ANGPT2 antagonizes the proangiogenic ANGPT1/Tie2 signaling and was described to be upregulated in ischemic murine heart and cause abnormal vascular remodeling (31). Leptin is reportedly expressed by perivascular PDGFRβ^+^ cells (32) and contributes to transplanted PCs’ proangiogenic activity in a mouse model of limb ischemia (33). In vivo studies using Matrigel plug–implanted PCs confirmed the ability of PD0325901 to induce the formation of vascular structures covered by DPCs.

We found that DPCs uniquely expressed 2 cardiac muscle–related genes, namely *TNNT2* (encoding cardiac troponin T2) and *ACTC1* (encoding actin α cardiac muscle 1). Although highly expressed in cardiomyocytes, these genes were previously found to be expressed in other cells. While a role for troponin T was suggested in the control of calcium-mediated SM contraction in various human organs (34), *ACTC1* transcript was upregulated in human microvascular ECs endowed with a better angiogenic response (35). Therefore, the expression of these cardiac transcripts appears compatible with the VSMC-like phenotype and the superior angiogenic properties of DPCs.

The PC shift toward a VSMC phenotype was characterized by a significant reduction in cell proliferation. The ERK1/-2/STAT3 axis controls the transcription of *CCND1*, whose encoded protein — cyclin D1 — is required for the activation of CDK4/6 and the progression of the cell cycle from G1 into the S phase (36, 37). The significant drop in the *CCND1* mRNA in DPCs versus PCs, combined with the failure of the selective CDK4/6 inhibitor ribociclib (38) to induce PC differentiation, suggests that cell cycle arrest and differentiation are parallel but mutually independent phenomena.

Two independent studies in vivo support the potential of the MEKi strategy in regenerative medicine. Exploiting the identification of 2 angiogenesis-related markers, CRABP2 and AQP1, uniquely expressed by cardiac DPCs, we could demonstrate that PD0325901 administration promoted a significant increase in the density and caliber of arterioles in the normoperfused heart, likely due to the growth of preexisting vessels to bigger arterioles, alongside the enhanced coverage by DPCs. Interestingly, total PDGFRβ^+^ PCs remained unchanged, thus indicating the increase in DPCs did not result in the exhaustion of the PC pool, which is important to preserve vascular homeostasis. The enhanced arteriolar bed was associated with, and may be responsible for, the observed increase in the resting myocardial blood flow, as, to the best of our knowledge, PD0325901 has not been reported to have vasodilatory activity. In the infarct model, the potentiation of arteriologenesis was associated with increased capillarization of the area at risk. These angiogenic capillaries may undergo muscularization through the recruitment and differentiation of PCs, thus supporting the growth of new arterioles.

The systemic administration of a drug implies a broad effect on different cell populations. Beyond vascular cells, we investigated the effects of the MEKi in other 2 cell types, namely cardiac fibroblasts and myocytes. The marked upregulation of αSMA in fibroblasts in vitro and the increased migration in a wound closure assay are compatible with the cell differentiation into myofibroblasts (39, 40). The frequency of the 2 phenotypes remained unchanged in normoperfused hearts after MEKi treatment. However, a different response could occur after MI. Importantly, we found that the infarct size was reduced in MEKi-treated hearts. This could be attributed to the protective effect of the increased neovascularization on the area at risk and greater scar compaction by migrated fibroblasts (41). Last, the histological analysis of the mouse hearts showed that the short course with the MEKi did not alter cardiomyocyte size (implying no hypertrophic remodeling occurred) and viability (confirming the safety of the treatment).

### Clinical relevance and study limitations.

This study suggests that myocardial PCs are endowed with intrinsic vascular plasticity, which can be pharmacologically evoked to encourage arteriologenesis. Short-duration treatment with PD0325901 may aid the recovery from MI through enhanced vascularization of the area at risk. The MEKi showed efficacy in reducing neointima formation in a mouse model of arterial stenosis (42), which increases the potential cardiovascular benefits of this class of compounds. Additional preclinical studies, including dose titration in large animal models, are warranted to demonstrate the benefit of repeated administration in diseases characterized by arteriole regression, such as diabetic cardiomyopathy and chronic ischemic heart failure. Also, further safety studies are necessary before translating our preliminary findings into clinical therapy for cardiovascular diseases, primarily because the use of MEKis still presents safety concerns. Indeed, prolonged MEKi administration was associated with an increased risk of developing arterial hypertension and decreased LVEF in cancer patients (43). The toxicity of these compounds is possibly due to intraorgan accumulation with time but might be less frequent during shorter treatments, like in our experimental model. Chronic treatment might require lowering the therapeutic dosage to avoid systemic and cardiac toxicity (44, 45). Importantly, our hypothesis-testing study may fuel the production of safer same-class compounds for specific cardiovascular applications.

Finally, we are aware that the drug may influence other cardiac cell types either directly or through indirect action mediated by the PCs. The discrimination is particularly problematic in an in vivo setting, given the reciprocal influence within the heart. Therefore, it is appropriate to avoid any overstatement regarding the possibility that differentiated PCs represent the only mechanism underpinning MEKi-induced in vivo benefit. Conversely, safety studies following this hypothesis-testing research are mandatory to assess the effect of this class of drugs on different cardiac cells before clinical use.

## Methods

Detailed procedures are described in the supplemental material.

### Derivation of primary cardiac PCs

Human and mouse PCs were immunosorted as CD31^–^CD34^+^ cells from human and mouse myocardial samples, as previously described (13). Briefly, samples were finely minced using scissors and a scalpel until nearly homogeneous and digested with Liberase (Roche) for up to 1 hour at 37°C, with gentle rotation. The digest was passed through 70-, 40-, and 30-μm strainers. Finally, the cells were recovered and sorted using anti-CD31 and -CD34 microbeads (Miltenyi Biotec) to deplete the population of CD31^+^ ECs and select CD31^–^CD34^+^ cells. Cells were expanded in Endothelial Cell Growth Medium 2 (ECGM2, PromoCell) employing human or mouse recombinant GFs, and used for experiments between passage 4 and 7.

### In vitro studies

All human cells were routinely tested negative for mycoplasma contamination. Differentiation of PCs and CASMCs (sourced from PromoCell) was achieved by culturing the cells for 10 continuous days either under GF depletion or PD0325901 (250 nM, Sigma-Aldrich) supplementation, with full media exchange every 48 hours. Functional in vitro assays included antigenic profile (by real-time qPCR, immunocytochemistry, and Western blotting), secretome (ELISA), contraction (embedding of cells in collagen gels), migration (wound healing assay), angiogenesis (2D Matrigel), calcium flux (Fluo-4 dye–based imaging of calcium), proliferation (EdU incorporation), and production of extracellular matrix. When required, cells were stimulated with the vasoconstrictor ET-1. Ribociclib (CDK4/6 inhibitor, TOCRIS) was employed to study the contribution of the cell cycle to PC differentiation. In selected experiments, cardiac fibroblasts (PromoCell and Lonza) and CAECs (PromoCell) were treated with PD0325901 (250 nM) to investigate the effects of the MEKi on other cell types. Antibodies for immunofluorescence in tissues and cells and Western blotting are listed in Supplemental Tables 8 and 9. Primers are listed in Supplemental Table 10.

### Next-generation RNA-Seq

Total RNA was extracted from human cardiac PCs either differentiated using 250 nM PD0325901 or treated with DMSO vehicle for 10 days (*n =* 3 each), and from human CASMCs employed as reference control (*n =* 2 donors). Strand-specific RNA-Seq was carried out using an Illumina HiSeq platform, with a 2 × 150 bp configuration and approximately 20 million reads per sample (GENEWIZ). Genes with an FDR less than 0.05 and absolute log_2_FC greater than 1 were considered DEGs. The data sets have been deposited in NCBI’s Gene Expression Omnibus (46) and are accessible through GEO accession number GSE195917 (https://www.ncbi.nlm.nih.gov/geo/query/acc.cgi?acc=GSE195917).

For discovery of transcripts unique to PCs and DPCs, we selected the top genes ranked by transcripts per million (TPM) and not expressed by the other cell populations. We selected genes with arbitrary average TPM of 200 or greater.

### In vivo studies

Three independent, randomized, controlled experiments were conducted on mice.

#### Study 1.

Male and female C57BL/6J mice (Charles River) were injected subcutaneously, on both flanks, with Matrigel plugs containing human PCs and either PD0325901 (500 nM) or vehicle (DMSO) (*n =* 4/group, equal gender distribution) and sacrificed 7 days later for the histological study of PC differentiation.

#### Study 2.

Female C57BL/6J mice (Charles River) received either PD0325901 (orally, 10 mg/kg/d) or vehicle (DMSO) for 14 days (*n =* 11/group). Endpoints were myocardial perfusion, LV performance (3D echocardiography), histological analysis of PC phenotypes, and vascular remodeling. Blood flow measurement employed carboxylate-modified green-fluorescent microspheres, following previously published protocols (47, 48). An additional cohort of mice (*n =* 3/group) was treated for only 5 days to assess the phosphorylation of ERK1/2 in the hearts and confirm the MEKi efficacy.

#### Study 3.

Female C57BL/6J mice (Hercus Taieri Resource Centre of the University of Otago) underwent permanent ligation of the left anterior descending coronary artery, followed by oral administration of PD0325901 (orally, 10 mg/kg/d) or vehicle (DMSO) for 14 days, starting from day 3 after MI (*n =* 12/group), according to an intention-to-treat randomized protocol. Endpoints included LV performance (3D echocardiography), vascularization, and scar size.

#### PD0315901 dose and administration route.

In Studies 2 and 3, PD0325901 was given orally and voluntarily to the mice once a day by including the compound in sugar-free strawberry-flavored jelly, as previously described (49, 50). PD0325901 was dissolved in DMSO and incorporated within the jelly. The control group received DMSO in jelly. Mice were given jelly at 8 μL/g body weight. Individual housing was necessary to observe jelly consumption. All the mice ate the entire jelly during the experiments; therefore, none were excluded from the study. Mice were trained to eat the jelly for 5 days before starting the 14-day experimental protocol, to ensure compliance with the treatment.

### Data availability

The data underlying this article will be shared on reasonable request from the corresponding authors. The RNA-Seq data sets have been deposited in NCBI’s GEO (accession number GSE195917).

### Statistics

Continuous variables are presented as mean ± SEM or SD of independent samples and as individual values. The D’Agostino-Pearson and Kolmogorov-Smirnov normality tests were used to check for normal distribution when applicable. Continuous variables normally distributed were compared using the Student’s *t* test (2-group comparison) or 1-way ANOVA (for multiple group comparisons). Two-way ANOVA was used to compare the mean differences between groups when appropriate. Nonparametric tests, including the Mann-Whitney *U* test (2-group comparison) and the Kruskal-Wallis test (multiple group comparison) were used to compare data not normally distributed. Post hoc analyses included Tukey’s and Dunn’s comparisons tests, as appropriate. Echocardiography parameters (baseline and final assessed in the same animal) were compared using paired tests; for all other analyses, unpaired tests were applied. For in vivo studies, post hoc analyses of outcomes were conducted according to the intention-to-treat principle. In Study 2, when baseline echo measurements were found to differ between groups, ANCOVA was used, as it provides the optimal statistical analysis in terms of bias, precision, and statistical power. In Study 3, due to the occurrence of missing values at the final measurements, we used a mixed-effects model 2-way ANOVA followed by Sidak’s multiple comparisons test to compare the vehicle-treated and PD0325901-treated groups. Significance was assumed when the *P* value was 0.05 or less. Analyses were performed using GraphPad Prism 8.0 and 9.0.

### Study approval

This study complies with the guidelines of the Declaration of Helsinki. Discarded material from congenital heart defect surgery was obtained with adult and pediatric patients’ custodians’ informed consent (ethical approval 15/LO/1064 from the North Somerset and South Bristol Research Ethics Committee). Donors and sample characteristics are described in Supplemental Table 11.

Animal studies were covered by licenses from the British Home Office (30/3373, PP1377882, and PFF7D0506) and the University of Otago, New Zealand (AEC10/14), and complied with the EU Directive 2010/63/EU. Procedures were carried out according to the principles stated in the NIH *Guide for the Care and Use of Laboratory Animals* (National Academies Press, 2011). Termination was conducted according to humane methods outlined in the Guidance on the Operation of the Animals (Scientific Procedures) Act 1986 Home Office (2014). The report of results is in line with the ARRIVE guidelines.

## Author contributions

EA and PM contributed the research conception and design. EA and PM wrote the manuscript. EA, RK, ACT, AC, DS, and M Carrabba conducted experiments and acquired data. EA, RK, ACT, AC, DS, and PM analyzed data. EA, RK, ACT, AC, DS, MM, and PM interpreted data. M Caputo recruited patients and provided human samples. PM provided funding and supervised the study. All authors approved the authorship order and the final version of the manuscript for publication.

## Supplementary Material

Supplemental data

## Version 1 03/29/2022

In-Press Preview

## Version 2 05/16/2022

Electronic publication
