# Supplementary material for: Cardiac pericyte reprogramming by MEK inhibition promotes arteriologenesis and angiogenesis of the ischemic heart
Source: J Clin Invest. 2022 May 16;132(10):e152308. doi: 10.1172/JCI152308 (PMC9106362; doi:10.1172/JCI152308)
Supplement: Supplemental data [file jci-132-152308-s035.pdf]

Cardiac pericyte reprogramming by MEK inhibition promotes arteriologenesis and angiogenesis of the ischemic heart

Elisa Avolio, Rajesh Katare, Anita C Thomas, Andrea Caporali, Daryl Schwenke, Michele Carrabba, Marco Meloni, Massimo Caputo, Paolo Madeddu

ONLINE SUPPLEMENTAL MATERIAL

Supplemental Figure 1

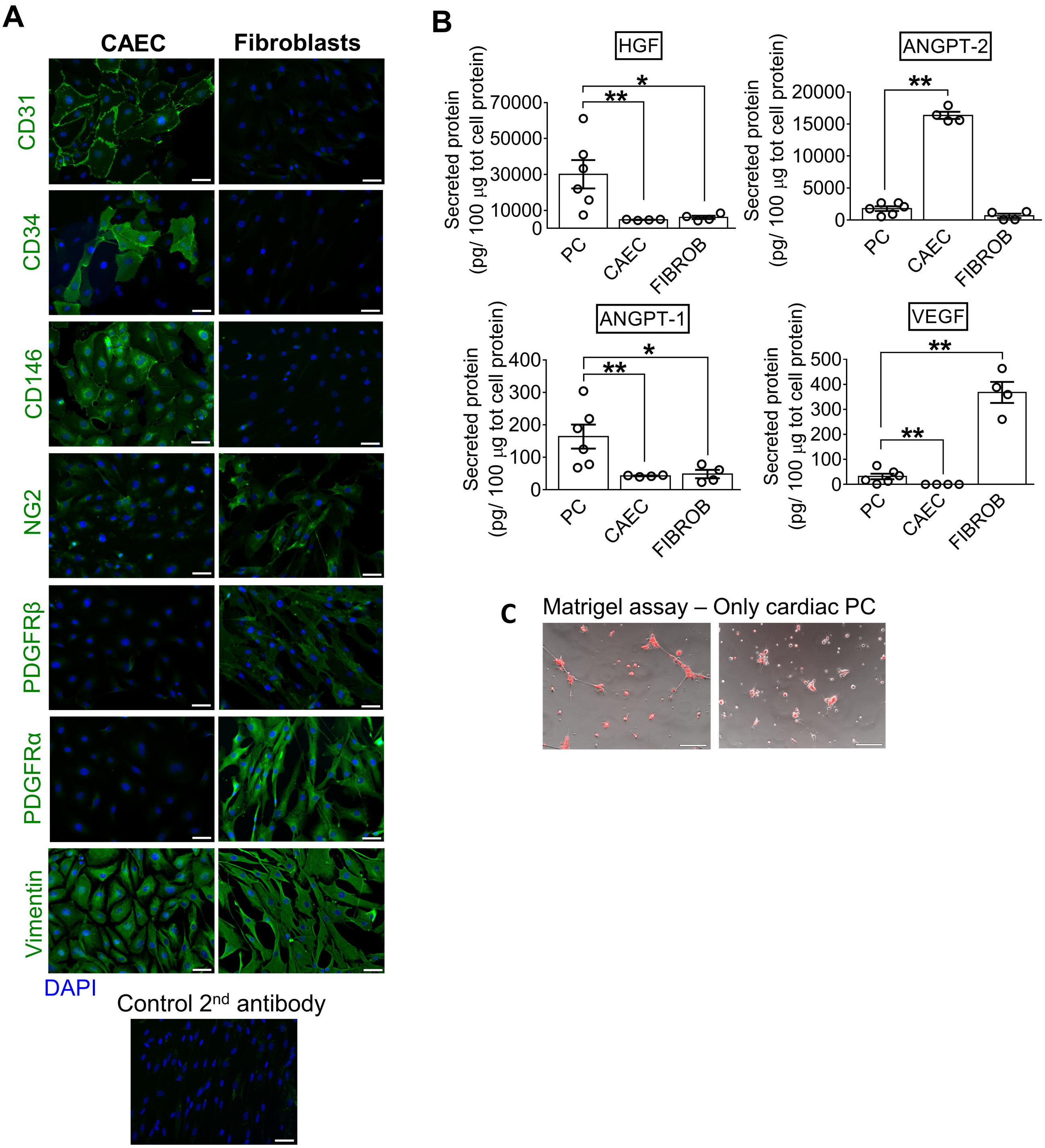

Supplemental Figure 1. Cellular controls for studies with human cardiac PC. (A) Immunofluorescence microphotographs showing expression of the indicated markers in human coronary artery endothelial cells (CAEC) and cardiac fibroblasts. Scale bar: 50 μm. Representative images are from one donor. **(B)** Cells' secretome. Comparison between PC, CAEC and fibroblasts. *n*=6 patients' PC, *n*=4 for CAEC and fibroblasts. For these latter, two different donors were used, each assayed in independent experimental duplicates. Data are plotted as individual values and means ± SEM. *Statistical test*: unpaired Mann-Whitney U test to compare PC with each control cell population. **P*<0.05, ***P*<0.01. **(C)** 2D-Matrigel assay: control represented by cardiac PC alone labelled with the red fluorescent tracker dil. PC failed to assemble in stable tubular networks. Scale bar: 100 μm.

Supplemental Figure 2

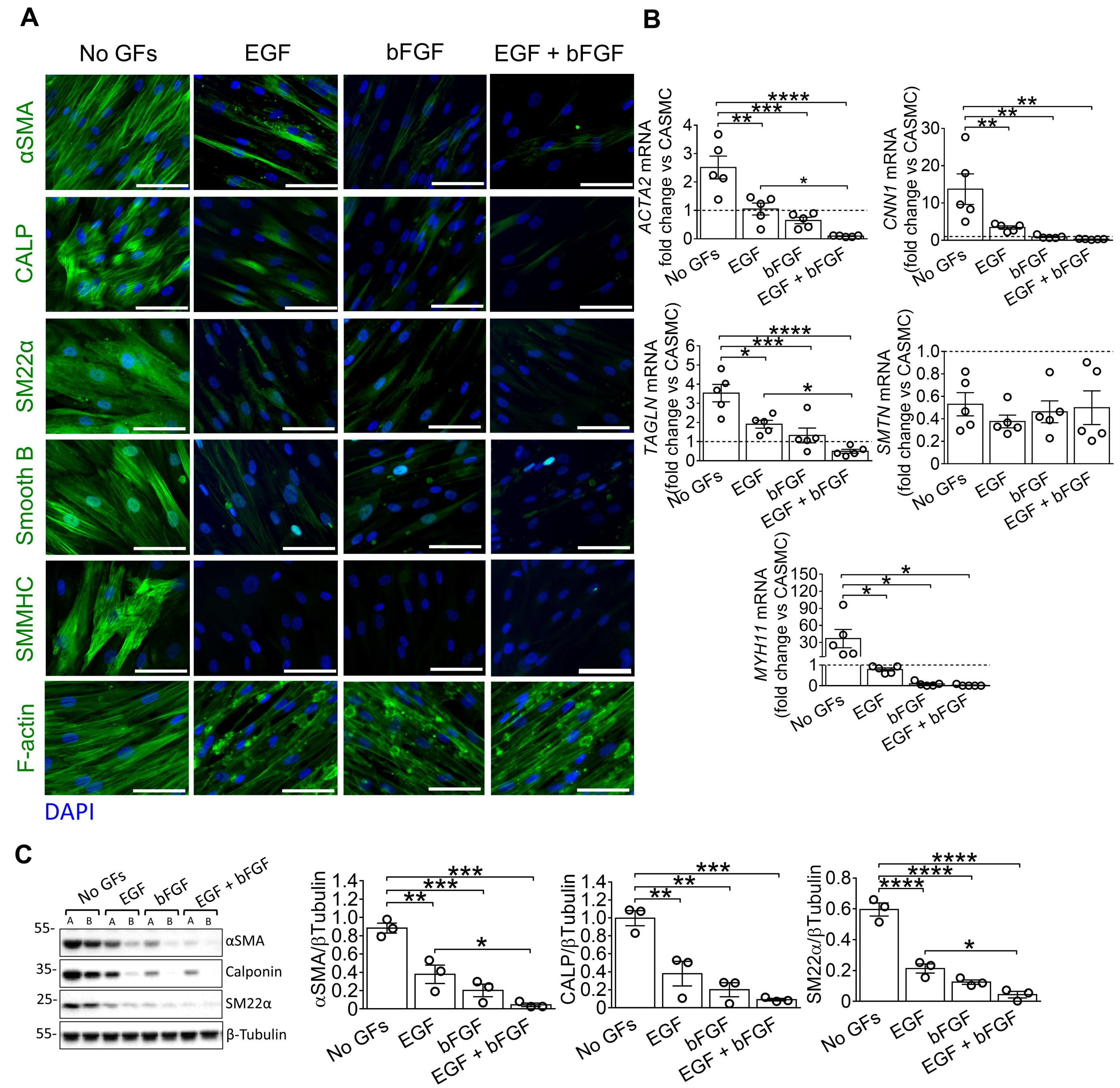

Supplemental Figure 2. EGF and bFGF control the expression of contractile VSMC genes in human cardiac PC. (A) Immunofluorescence images showing the expression of VSMC markers and the general cytoskeletal protein F-actin by PC. Cells were cultured for 10 days. Scale bar: 50 µm. Representative images are from one patient. **(B)** Transcriptional analysis of VSMC genes in PC and differentiated cells. Dashed lines indicate coronary artery SMC (CASMC) used as reference cell population. Values are expressed as a fold change vs CASMC. *n*=5 patients' PC. **(C)** Western blot analysis of VSMC proteins. In representative blots A and B indicate cells from two patients. Graphs summarise data for *n*=3 patients' PC. Data are reported as individual values and means ± SEM. *Statistical test*: ordinary two-way ANOVA followed by Tukey's multiple comparisons test. * *P*<0.05, ** *P*<0.01, *** *P*<0.001, **** *P*<0.0001.

Disclosure: the same *No GF* and *EGF+bFGF* images are presented in Figure 2 as they are part of the same experiment comparing with single GFs.

Supplemental Figure 3

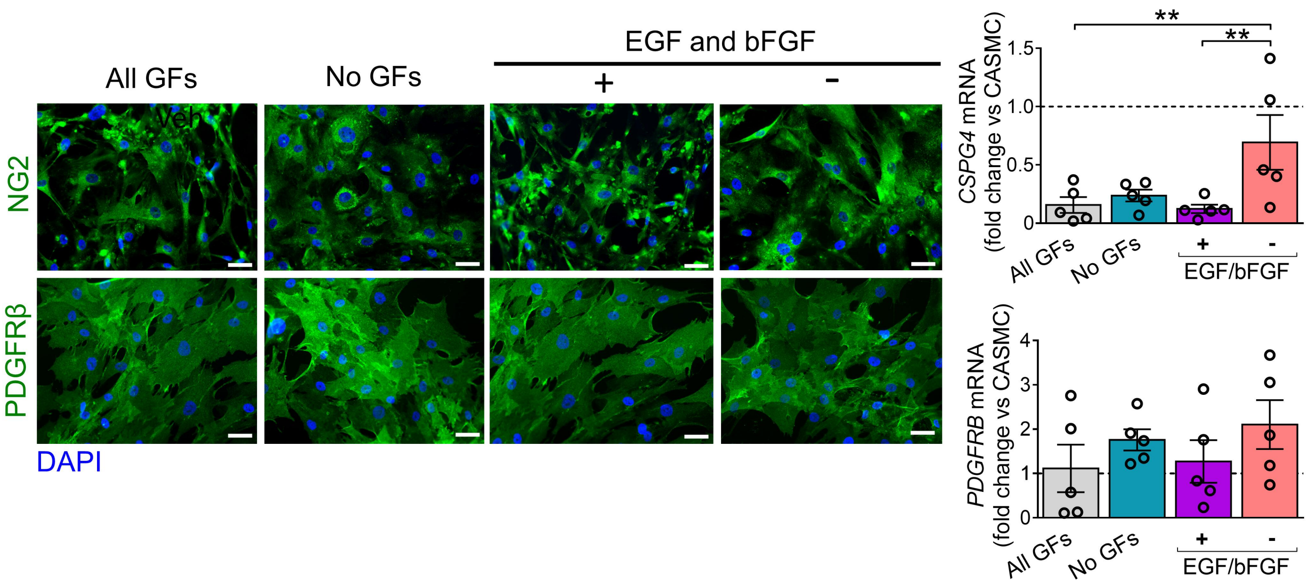

Supplemental Figure 3. Differentiated human cardiac PC retain the expression of common pericyte/VSMC genes. (A) Immunofluorescence images showing the expression of NG2 and PDGFRβ by PC and derived VSMC-like cells following culture for 10 days with different media as indicated. Scale bar: 50 µm. Representative images are from one patient. **(B)** Transcriptional analysis of the two genes. The dashed line indicates coronary artery SMC (CASMC) used as reference cell population. Values are expressed as a fold change vs CASMC. *n*=5 patients' PC. Data are reported as individual values and means ± SEM. *Statistical test*: ordinary two-way ANOVA followed by Tukey's multiple comparisons test. ** *P*<0.01.

Supplemental Figure 4

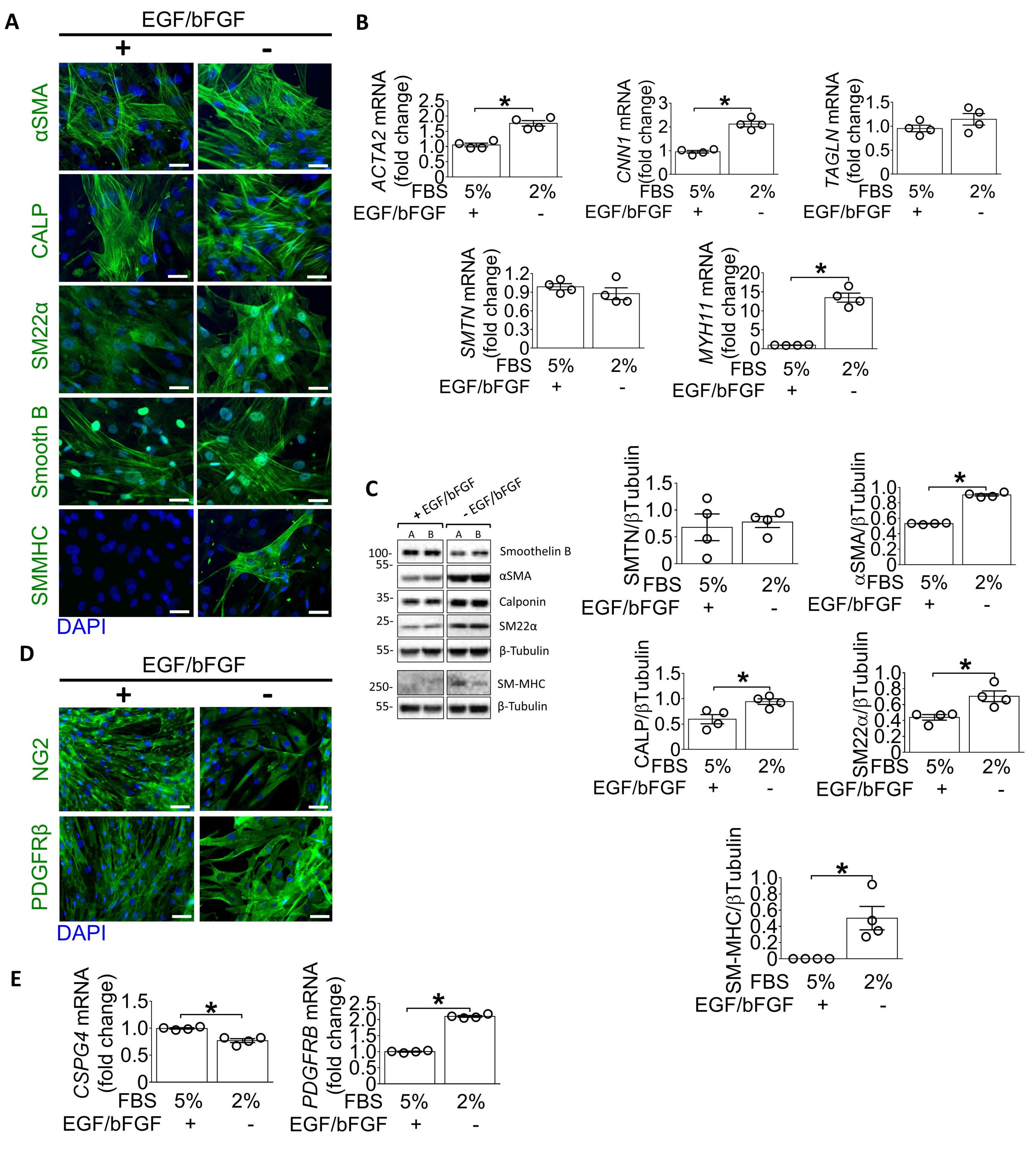

Supplemental Figure 4. Human coronary artery SMC (CASMC) were used as a positive control for experiments with cardiac PC. Cells were cultured for 10 days with their growth media in the presence or absence of GFs as indicated. Depletion of GFs and reduction of the % of FBS were applied to stimulate the differentiation of cells into the contractile phenotype. **(A)** Immunofluorescence images showing expression of VSMC markers. Scale bar: 50 μm. **(B)** Transcriptional analysis of VSMC genes in control and differentiated cells. Values are expressed as a fold change vs control CASMC. **(C)** Western blot analysis of VSMC proteins. A and B indicate two different donors. **(D)** Immunofluorescence images showing expression of pericyte/VSMC markers. Scale bar: 50 μm. **(E)** Transcriptional analysis of pericyte/VSMC genes in control and differentiated cells. Values are expressed as a fold change vs control CASMC. All graphs summarise data for *n*=4 samples (2 donors, two independent replicates each). Data are illustrated as individual values and means ± SEM. *Statistical test*: unpaired Mann-Whitney U test. * *P*<0.05 vs control cells. Representative images are from one donor.

Supplemental Figure 5

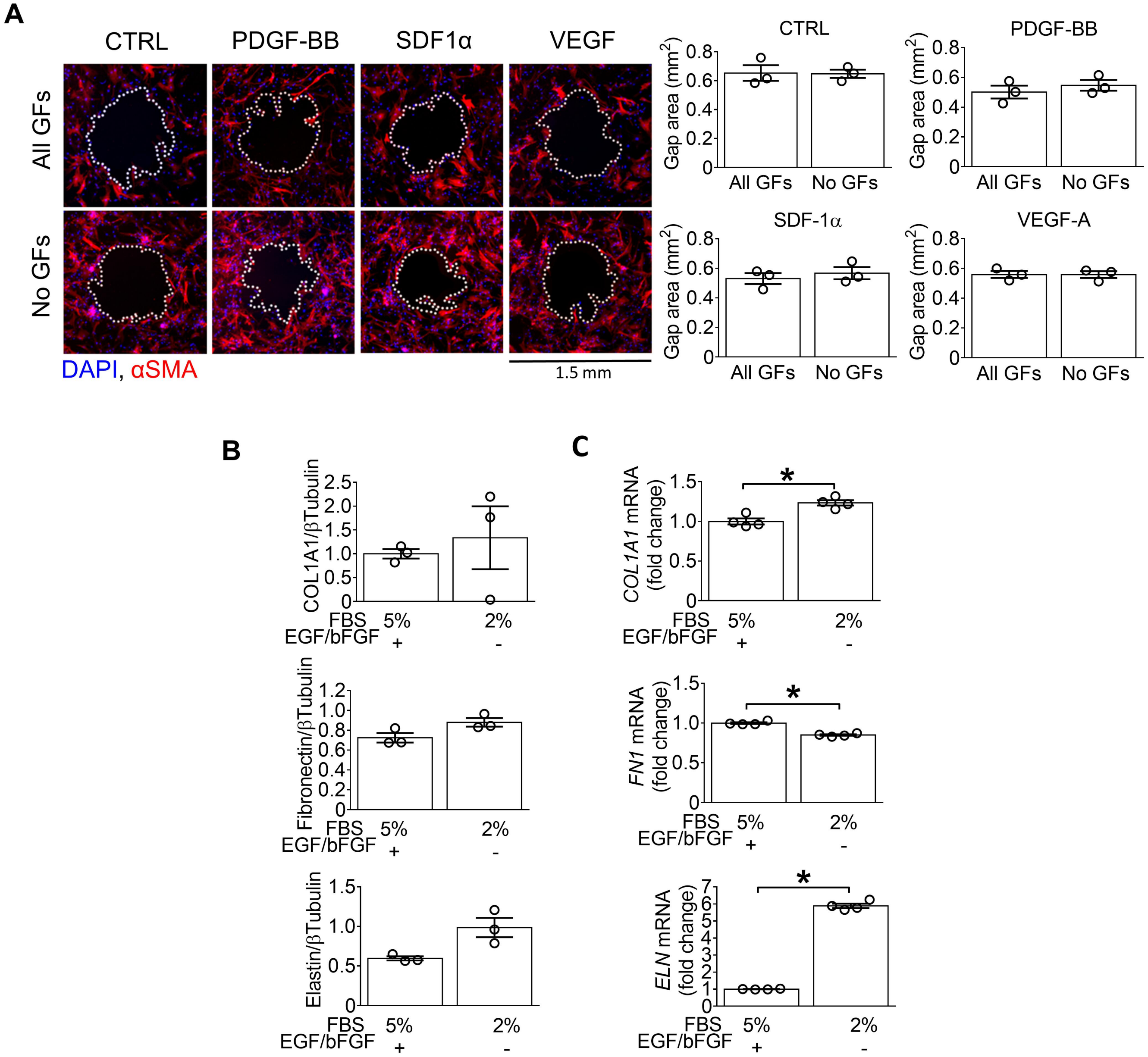

Supplemental Figure 5. Human coronary artery SMC (CASMC) were used as a control in functional assays with PC. (A) Gap closure migration assay. Migration time is 24h. Cells were differentiated for 10 days in the absence of EGF/bFGF and reduced FBS (2%) and then used in the migration assay. Control cells were cultured with full GF and FBS medium. *n*=3 donors. Representative images are from one patient. **(B-C)** Expression of extracellular matrix (ECM) proteins. **(B)** Expression of Collagen I, Elastin and Fibronectin in control and differentiated CASMC using Western blotting. *n*=3 donors. **(C)** Transcriptional analysis of ECM markers. *n*=4 (2 donors assayed in independent duplicates). Data are illustrated as individual values and means ± SEM. *Statistical test*: unpaired Mann-Whitney U test. * $P<0.05$.

Supplemental Figure 6

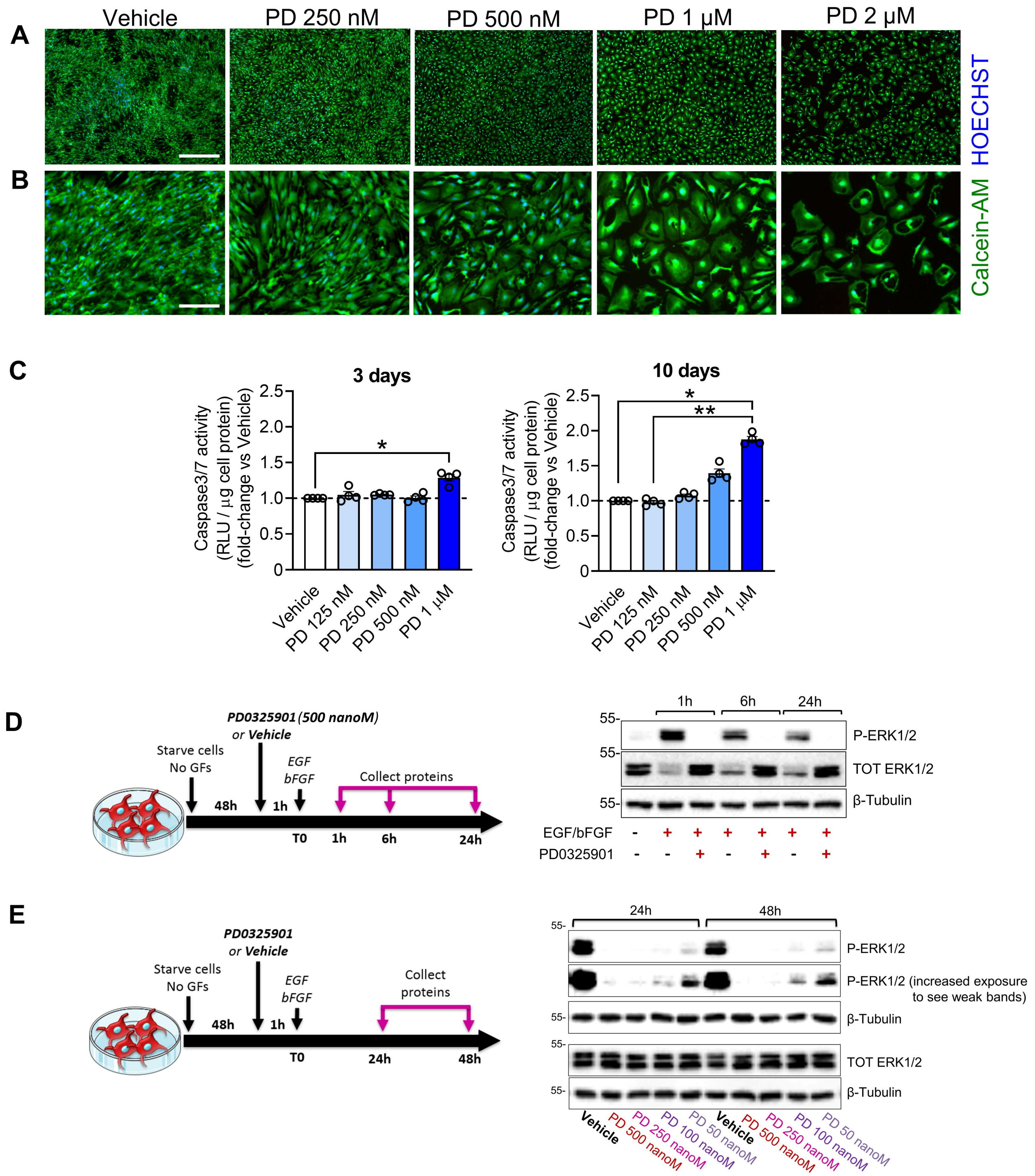

Supplemental Figure 6. Dose-response study to assess PD0325901 efficacy and cytotoxicity in human cardiac PC. (A&B) PC were cultured with four increasing doses of PD0325901 for nine days. The DMSO corresponding to the higher concentration of the drug was used as control. The Calcein-AM/EthDIII viability assay was performed to assess cell viability. Viable cells have a green cytoplasm, while dead cells are identified by red nuclei. *n*=2 patients' PC. Representative images are from one patient. Scale bars: (A) 1 mm; (B) 200 µm. **(C)** Apoptosis assay. Caspase 3/7 activity was measured as relative luminescence units (RLU) and normalised versus the total cellular protein content and finally expressed as a fold-change vs vehicle. *n*=4 patients' PC. Values are means ± SEM. *Statistical test*: unpaired Kruskal-Wallis followed by Dunn's multiple comparisons test. **P*<0.05, ***P*<0.01. **(D)** The drug used at a concentration of 500 nM was able to prevent ERK1/2 phosphorylation in PC (*n*=1 patient) for up to 24h. **(E)** Dose-response study to determine the minimum dosage of the drug able to prevent ERK1/2 phosphorylation for up to 48h. (*n*=1 patient). In (D&E), cells were initially starved for 48h to switch off intracellular signalling, then pre-treated with the drug/vehicle for 1h before stimulation with EGF and bFGF.

Supplemental Figure 7

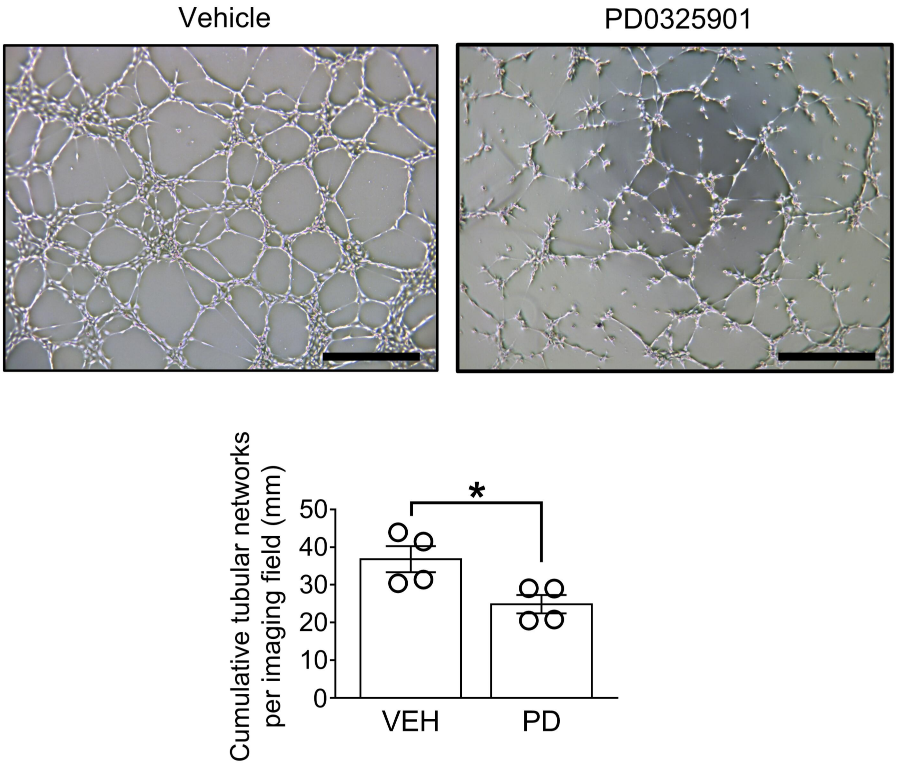

Supplemental Figure 7. Effect of PD0325901 treatment on coronary artery EC angiogenic activity. EC, cultured in full GF medium, were pre-conditioned for 5 days with 250 nM PD0325901 or vehicle, with media replacement every 2 days. At the end of this period cells were seeded on the top of Matrigel and images snapped after 5 hours. Scale bar: 0.5 mm. *n*=2 different donors, each assayed in independent experimental duplicates, for a total *n*=4. Data are plotted as individual values and means ± SEM. *Statistical test*: unpaired Mann-Whitney U test. * P<0.05. **Veh**: vehicle. **PD**: PD0325901.

Supplemental Figure 8

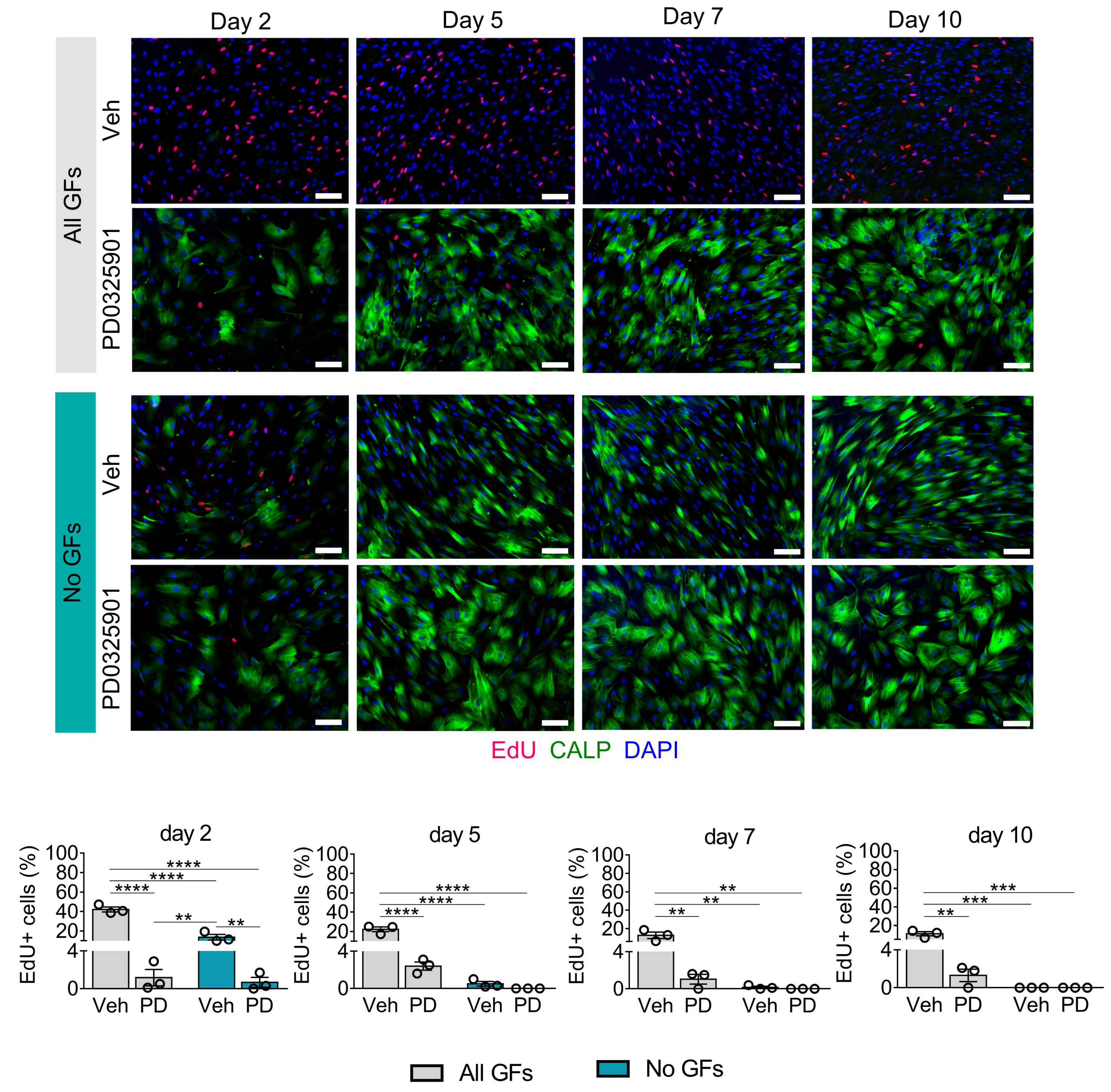

Supplemental Figure 8. Effects of PD0325901 treatment on PC proliferation. PC were cultured for 10 days with either *All GFs* or *No GFs*. Media were supplemented with 250 nM PD0325901 or vehicle, with replacement every 2 days. Cells were incubated with EdU for 24h during days 2, 5, 7, 10. In the immunofluorescence images, EdU is shown in red, Calponin in green. Scale bar: 100μm. Representative images are from one patient. *n*=3 patients' PC. Data are plotted as individual values and means ± SEM. *Statistical test*: ordinary two-way ANOVA followed by Tukey's multiple comparisons test. ** P<0.01, *** P<0.001, **** P<0.0001. **Veh**: vehicle. **PD**: PD0325901.

Supplemental Figure 9

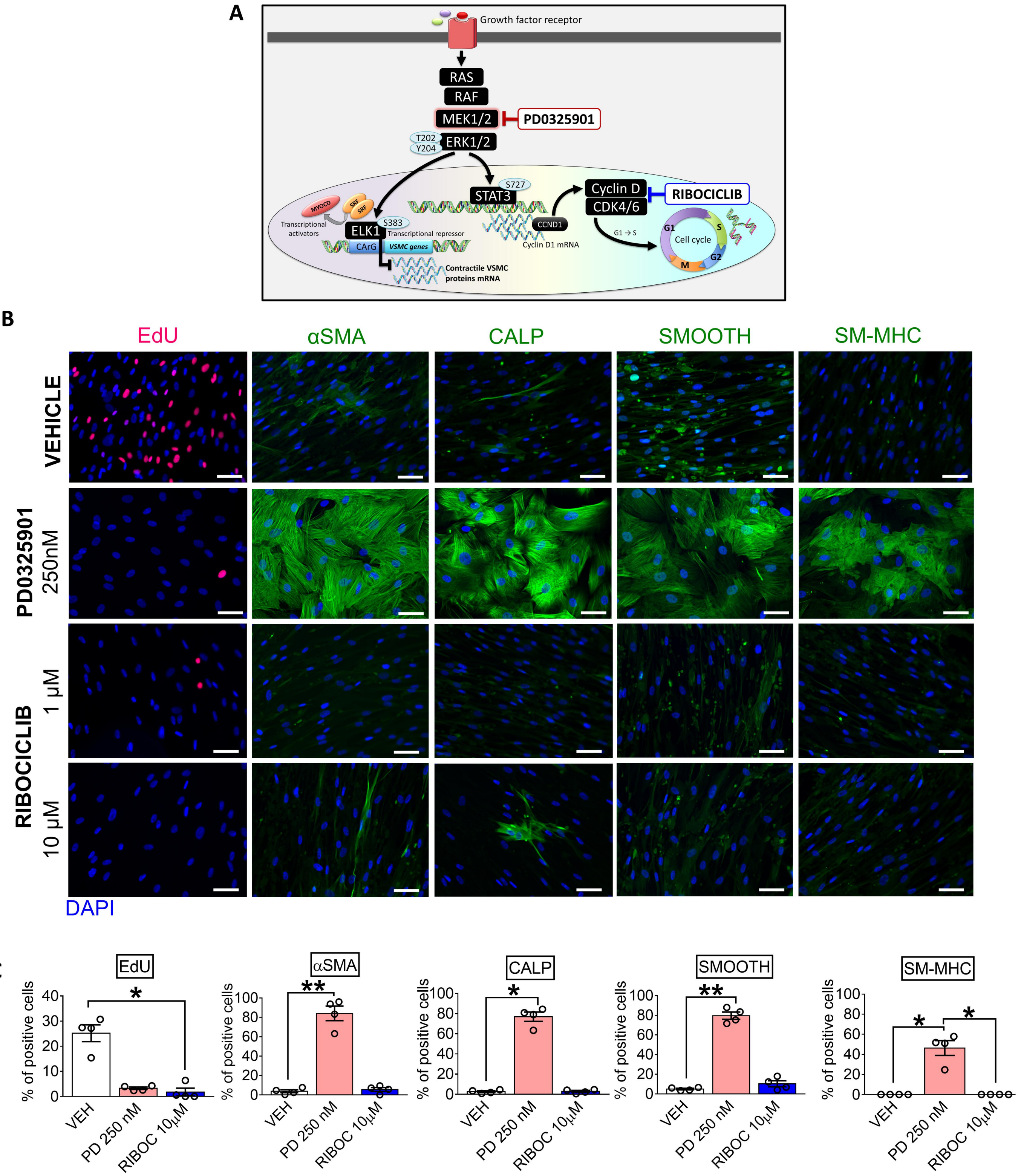

Supplemental Figure 9. Comparison of the effects of MEK inhibition and cell cycle arrest on PC differentiation. (A) Cartoon showing the pharmacological inhibitors used and respective targets. In addition to PD0325901, we used a potent, specific inhibitor of the cell cycle – Ribociclib, compound that blocks the activity of the cyclin D1/cyclin dependent kinases 4/6 complexes, thus preventing the G1 to S phases transition. **(B)** Immunofluorescence images showing cell proliferation (EdU) and the expression of VSMC markers by PC cultured for 10 days with all GFs and the different drugs as indicated. EdU was added to the culture medium for 48 h during the last 2 days of the protocol. Scale bar: 50 µm. Representative images are from one patient. **(C)** Quantification of cells expressing the different markers. *n*=4 patients' PC. Data are plotted as individual values and means ± SEM. *Statistical test*: unpaired Kruskal-Wallis followed by Dunn's multiple comparisons test. * *P*<0.05, ** *P*<0.01.

Supplemental Figure 10

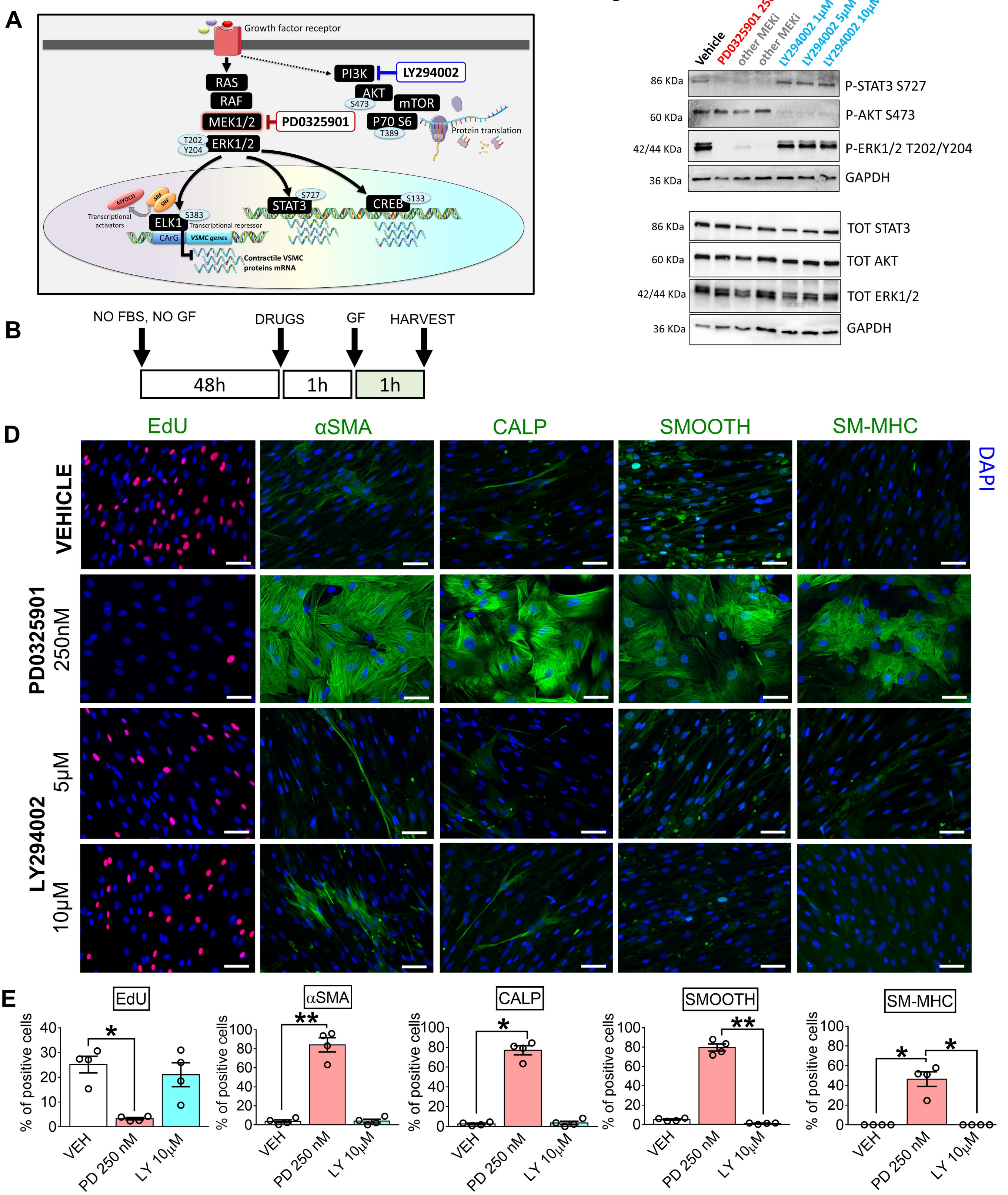

Supplemental Figure 10. Effects of PI3K/AKT axis inhibition on PC differentiation. (A) Cartoon showing the inhibitors employed and respective targets. In addition to PD0325901, we tested the PI3K inhibitor LY294002, that prevents downstream AKT phosphorylation/activation. **(B-C)** Signalling study using western blot showing the compounds efficacy in cardiac PC treated with the drugs for 1 hour before stimulation with GFs (all GFs present in the PC medium). *n*=2 patient PC. Representative blots are from one patient. **(D)** Immunofluorescence images showing the expression of the proliferation marker EdU and VSMC antigens by PC cultured for 10 days with the different drugs as indicated. EdU was added to the culture medium for 48 h during the last 2 days of the protocol. Scale bar: 50 μm. The experiment was performed with *n*=4 patients' PC. Representative images are from one patient. **(E)** Quantification of cells expressing the different markers. *n*=4. Data are plotted as individual values and means ± SEM. *Statistical test*: unpaired Kruskal-Wallis followed by Dunn's multiple comparisons test. * $P<0.05$, ** $P<0.01$.

Disclosure: the same Vehicle and PD0325901 images are presented in Supplemental Figure 9 as they are part of the same experiment comparing different drugs with PD0325901.

Supplemental Figure 11

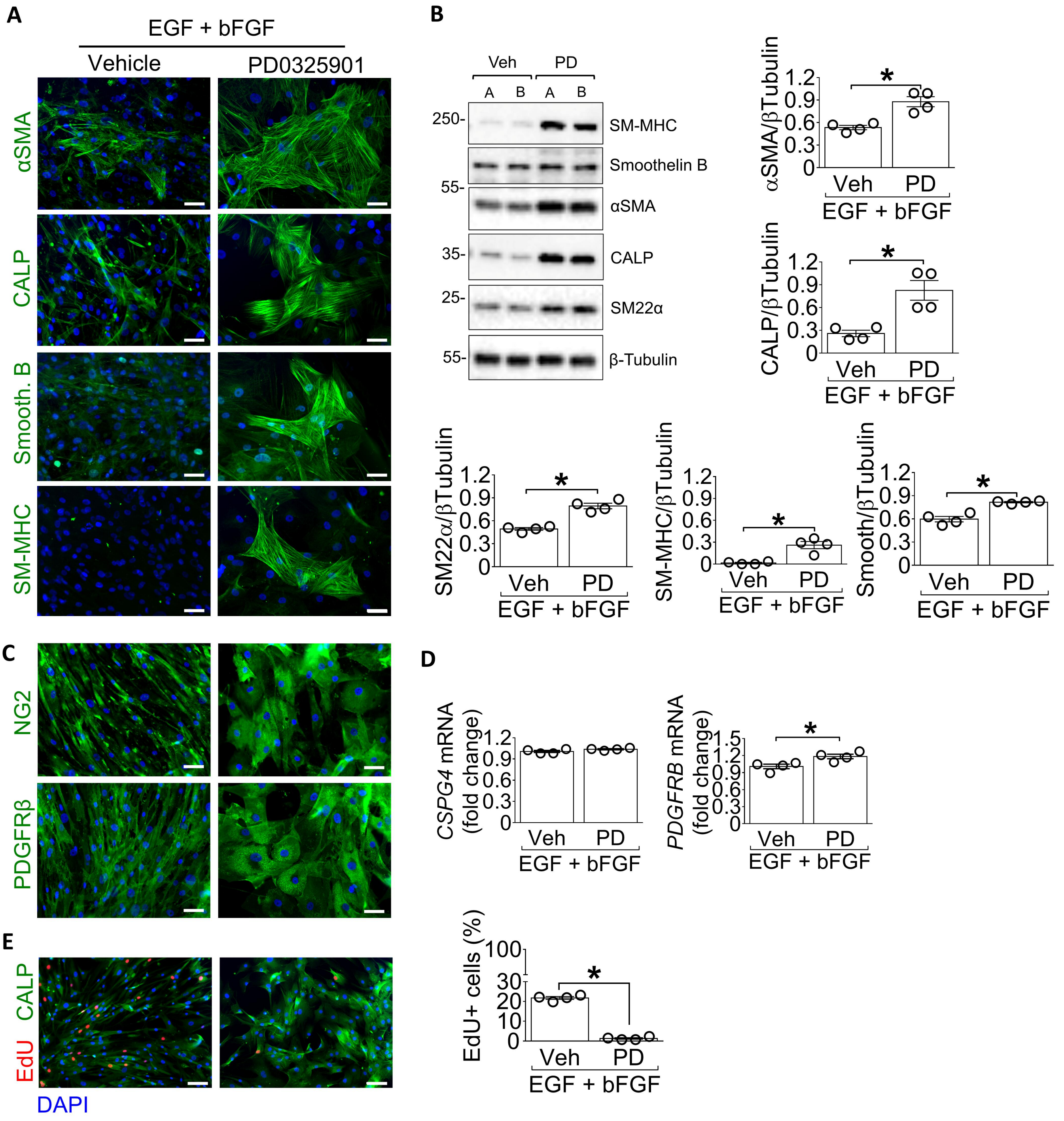

Supplemental Figure 11. PD0325901 promotes the expression of contractile markers in human coronary artery SMC (CASMC). Cells were cultured for 10 days with the complete medium containing EGF and bFGF supplemented with either 250 nM PD0325901 or vehicle, with media replacement every two days. Cells receiving the drug upregulated contractile VSMC markers, as showed by immunofluorescence **(A)** and western blotting **(B)** analyses. Scale bar: 50 µm. Representative images are from one donor. Representative blots are from two donors (A and B). **(C-D)** Expression of pericyte/VSMC markers assessed by immunocytochemistry and qPCR. Scale bar: 50 µm. **(E)** Proliferation assay. Cells were incubated with EdU for 24h starting at day 3. EdU is shown in red, Calponin in green. Scale bar: 100 µm. In all graphs, *n*=4 (*n*=2 donors, 2 independent replicates each). **Veh**: vehicle. **PD**: PD0325901. Data are illustrated as individual values and means ± SEM. *Statistical test*: unpaired Mann-Whitney U test. * *P*<0.05.

Supplemental Figure 12

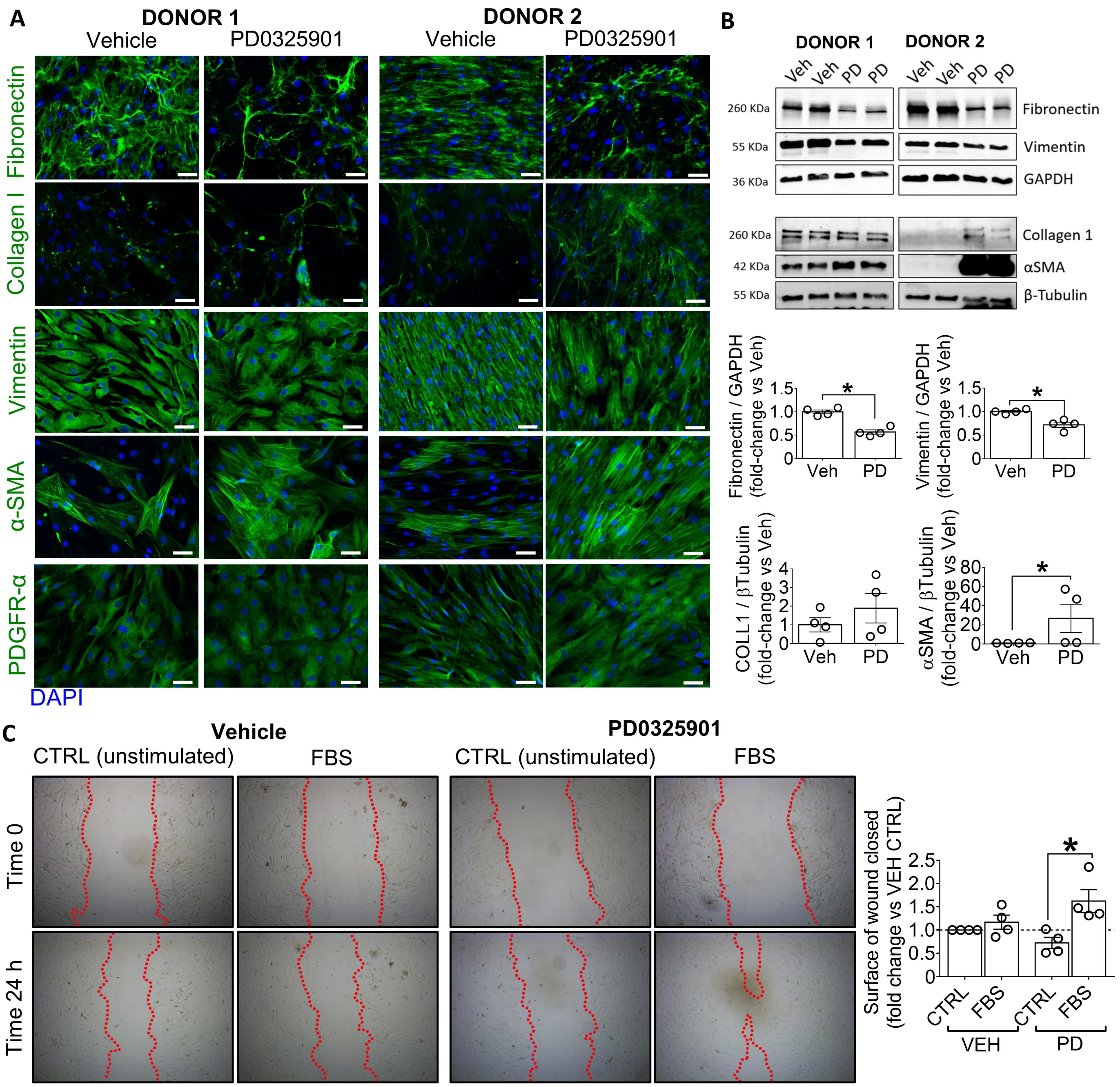

Supplemental Figure 12. Effect of PD0325901 treatment on cardiac fibroblasts differentiation and migration. Fibroblasts, cultured in full GF medium, were pre-conditioned for 7 days with 250 nM PD0325901 or vehicle, with media replacement every 2 days. At the end of this period cells were used for functional assays. n=2 different donors, each assayed in independent experimental duplicates, for a total n=4. **(A)** Immunofluorescence staining for fibroblasts and myofibroblasts markers (in green). Scale bar: 50 µm. **(B)** Western blot and graphs showing blots densitometry. Data are plotted as individual values and means ± SEM. *Statistical test*: unpaired Mann-Whitney U test. * P<0.05. **(C)** Wound closure assay. A scratch was created in confluent fibroblasts and images taken at baseline (time 0). Cells were incubated with the migration stimulus FBS for 24 h and final images were recorded. Control cells were not stimulated. The surface of wound closure was calculated as % of the baseline area and expressed as fold-change vs vehicle control. *Statistical test*: unpaired Kruskal-Wallis followed by Dunn's multiple comparisons test. * P<0.05. Representative images are from one donor. **Veh**: vehicle. **PD**: PD0325901. **CTRL:** control.

Supplemental Figure 13

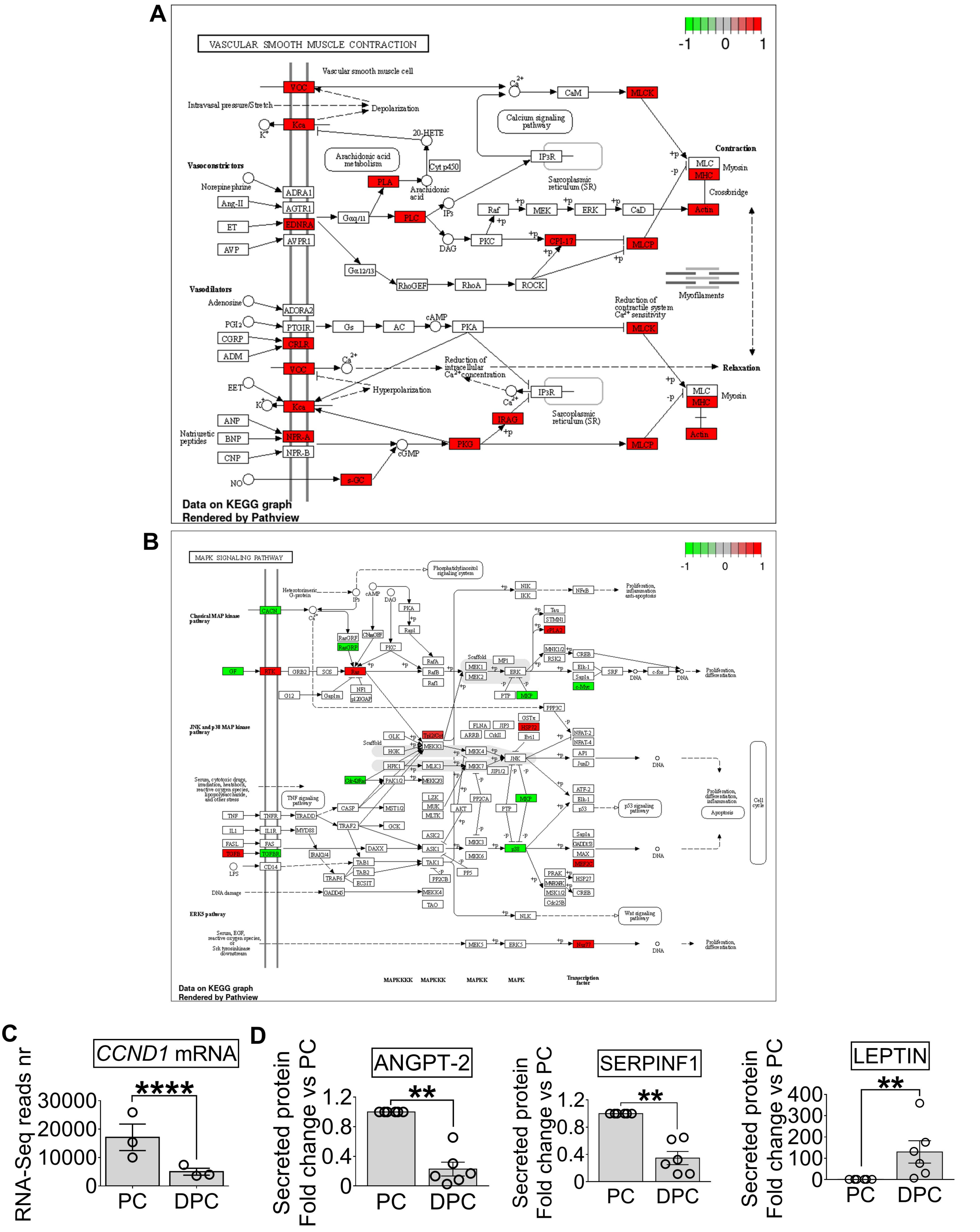

Supplemental Figure 13. (A&B) Transcriptomics analysis of PC differentiated with PD0325901 (DPC) and naïve PC. Schematic view of the main differentially regulated hits emerging from the pathways **(A)** “Vascular smooth muscle contraction” and **(B)** “MAPK signalling”**. (C) Expression of cyclin D1 mRNA.** Log2FC -1.77, *p*-value 0.0000481. *n*=3 patients’ PC. **(D) Validation of main angiogenic proteins emerged by the RNA-Seq analysis.** Secreted factors were measured using ELISA in the cell conditioned medium, normalised versus the total intracellular protein content and finally expressed as a fold change vs undifferentiated PC. *n*=6 patients’ PC. *Statistical test*: unpaired Mann-Whitney U test. ** *P*<0.01.

Supplemental Figure 14

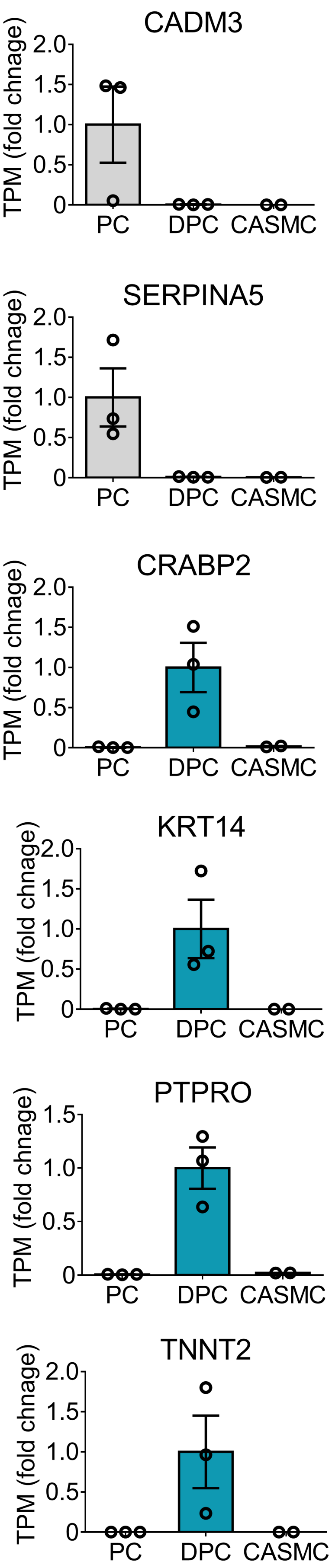

Supplemental Figure 14. Top genes selectively expressed by naïve PC and PD0325901-differentiated PC (DPC). Genes were selected as the top genes ranked by transcripts per million (TPM) of the positive population, and not expressed by the other cell populations. Bar graphs show the fold-change TPM expression. CAMSC: reference coronary artery smooth muscle cells. Data are means ± SEM.

Supplemental Figure 15

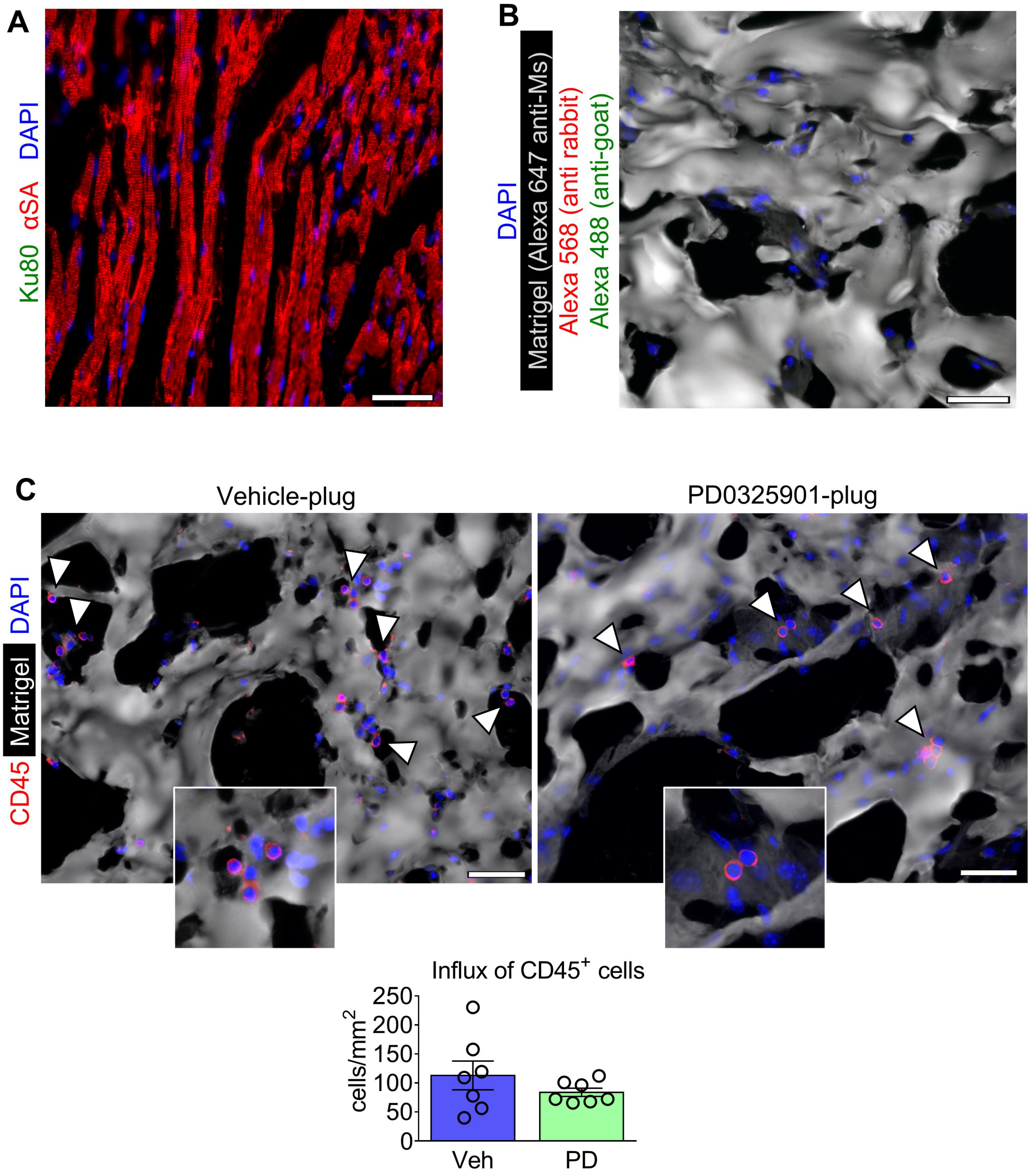

Supplemental Figure 15. Matrigel plugs *in vivo* experiment. (A) Immunostaining for human Ku80 in mice hearts ruled out the cross-reactivity of the antibody with mouse tissues. **(B)** Secondary antibody controls in Matrigel plugs. A secondary antibody Alexa 647 anti-mouse was used to mark the structure of the matrix (Matrigel matrix derived from mouse sarcoma). **(C)** Influx of CD45+ immune/inflammatory cells in the Matrigel plugs. Immunofluorescence images showing CD45+ cells in red and the matrix in the pseudo-colour white. Arrowheads point to CD45+ cells. Graphs indicate the density of CD45+ cells in the plugs, expressed as number of cells per mm^2 of area. *n*=7 plugs (from 4 mice). All scale bars: 50 µm. **Veh**: vehicle. **PD**: PD0325901. Data are illustrated as individual values and means ± SEM. *Statistical test*: unpaired Mann-Whitney U test.

Supplemental Figure 16

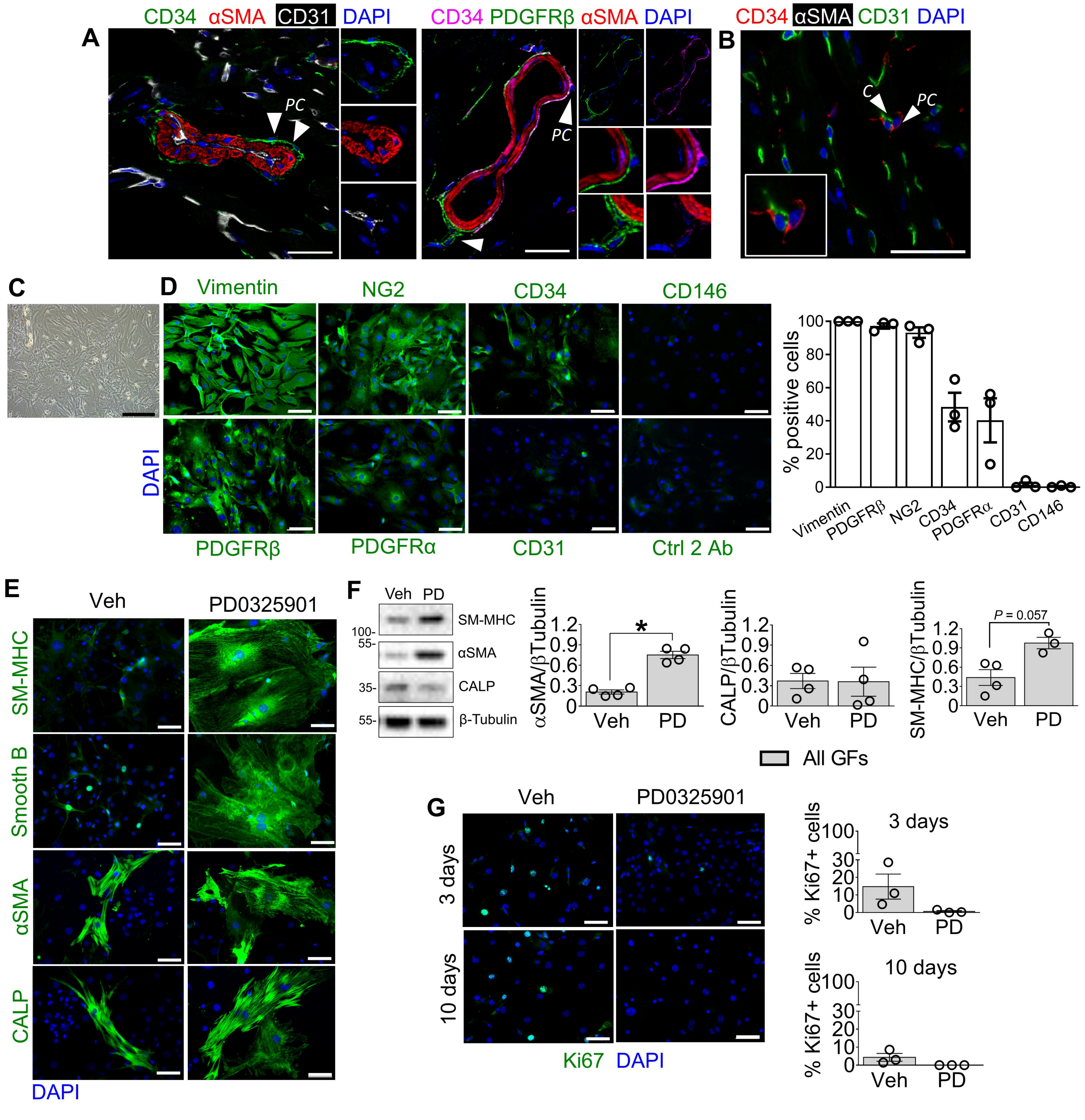

Supplemental Figure 16. Effects of PD0325901 on murine cardiac PC. (A&B) Confocal immunofluorescence images of mouse hearts. Arrows point to CD31-neg αSMA-neg CD34-pos PDGFRβ-pos PC around arterioles (A) and capillaries (B). *C*: capillary. *PC*: pericyte. Scale bars: A&B: 20 µm, C: 25 µm. **(C)** Brightfield microphotograph of expanded PC at passage 4. Scale bar: 100 µm. **(D)** Antigenic profile of expanded PC at passage 4. *n*=3 mice PC. Scale bar: 50 µm. Representative images are from one mouse. **(E&F)** PC differentiation into VSMC-like cells using PD0325901. PC were cultured with *All GFs* in the presence of 250 nM PD0325901 or vehicle for 10 days, with media replacement every 2 days. (E) Immunofluorescence images show the expression of VSMC markers. Scale bar: 50 µm. Representative images are from one mouse. (F) Blots showing representative bands from one mouse and graphs reporting blots densitometry. *n*=4 mice PC. **(G)** PC proliferation after 3 and 10 days of PD0325901 treatment. Immunofluorescence images show Ki67 in green and graphs report quantitative analysis. *n*=3 mice PC. Scale bar: 50 µm. Representative images are from one mouse. **Veh**: vehicle. **PD**: PD0325901. Data are illustrated as individual values and means ± SEM. *Statistical tests*: unpaired Mann-Whitney U test. * $P<0.05$.

Disclosure: the image in A (second panel) was presented also in Figure 10J as they are part of the same experiment investigating peri-arteriolar PC.

Supplemental Figure 17

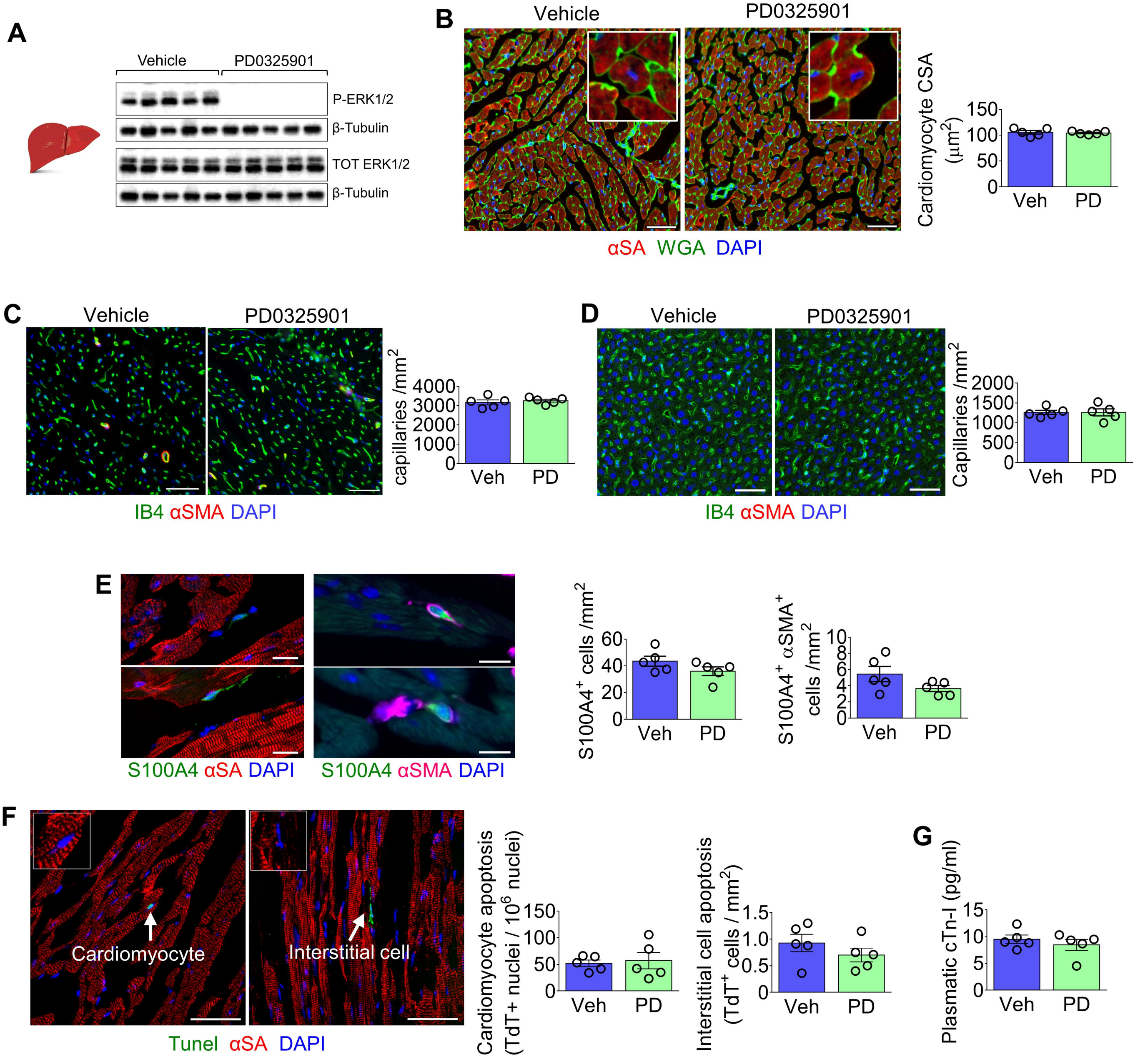

Supplemental Figure 17. Efficacy and effects of PD0325901 treatment in healthy mice. (A) Western blotting for P-ERK on total liver proteins. **(B)** Measurement of the cardiomyocyte cross sectional area (CSA). Values are reported as average CSA per mouse. Cardiomyocytes are identified by the red fluorescence of α-Sarcomeric actin (αSA), Wheat Germ Agglutinin (WGA, green) was used to identify the cardiomyocytes borders for an accurate measurement of the cell area. Scale bar: 25 μm. **(C&D)** Capillary density in the mice heart (C) and liver (D). Capillaries are identified by Isolectin B4 (green), arterioles by αSMA (red). Scale bar: 50 μm. Results are expressed as number of capillaries per tissue area. **(E)** Analysis of fibroblasts in the mice heart. Fibroblasts are recognised by expression of the antigen S100A4 (green), myofibroblasts by the co-expression of S100A4 and αSMA (magenta), cardiomyocytes are labelled by αSA (red). Scale bar: 20 μm. Results are expressed as number of cells per tissue area. **(F)** Analysis of cell apoptosis in the mice hearts. Arrows point to apoptotic nuclei (green). Cardiomyocytes are identified by the red fluorescence of αSA. Apoptotic cardiomyocytes are expressed as a fraction of total cardiomyocytes. Apoptotic αSA-negative interstitial cells are expressed as number of cells per mm^2 of myocardial area. Scale bar: 50 μm. **(G)** ELISA for Cardiac Troponin T in mice plasma. According to guidelines for mice, healthy animals have cTn-I < 15 ng/mL. For all analyses, *n*=5 mice. **Veh**: vehicle. **PD**: PD0325901. All data are illustrated as individual values and means ± SEM. *Statistical tests*: unpaired Mann-Whitney U test.

Supplemental Table 1. Markers of contractile VSMC phenotype investigated in this study.

PROTEIN	GENE	FUNCTION	PHENOTYPE SPECIFICITY
**Smooth muscle α-actin (α-SMA)**	***ACTA2***	Contractile filament<br>Force generation	***Early-intermediate contractile***
**Transgelin (SM22α)**	***TAGLN***	Actin associated<br>Modulation of contraction	***Intermediate contractile***<br>Restricted to smooth muscle in adults
**Smooth muscle basic Calponin (CALP)**	***CNN1***	Actin associated<br>Regulation of contraction	***Intermediate contractile***
**Smoothelin B**	***SMTN***	Actin associated<br>Regulation of contraction	***Contractile***<br>Predominantly expressed in the vasculature of the adult. Specific to VSMC
**Myosin heavy chain, smooth muscle isoform (SM-MHC)**	***MYH11***	Contractile filament<br>Force generation	***Contractile***<br>The most specific marker of contractile VSMC. Rapidly lost by cultured VSMC

Supplemental Table 2 - Top 30 differentially expressed genes in DPC vs. CASMC. The list includes the 15 most upregulated and 15 most downregulated genes.

Gene	Encoded protein	Log2FC DPC vs CASMC	*p*-Value
ACTC1	Actin Alpha Cardiac Muscle 1	+14.35944897	1.19E-10
KRT14	Keratin 14	+14.12078901	4.17E-18
XIST	X Inactive Specific Transcript	+13.02662598	0.003056818
OMD	Osteomodulin	+12.84812084	9.30E-05
SULT1E1	Sulfotransferase Family 1E Member 1	+12.81940701	3.63E-17
ZBTB16	Zinc Finger And BTB Domain Containing 16	+12.44644752	8.93E-15
TNNT2	Troponin T2, Cardiac Type	+12.40191342	3.03E-09
IGF2	Insulin Like Growth Factor 2	+12.12131439	2.08E-22
APOB	Apolipoprotein B	+11.95344066	2.47E-12
MYH11	Myosin Heavy Chain 11	+11.59188659	2.16E-78
CCDC190	Coiled-Coil Domain Containing 190	+10.40053405	5.04E-21
IQCA1	IQ Motif Containing With AAA Domain 1	+10.26011211	5.62E-09
SMOC2	SPARC Related Modular Calcium Binding 2	+10.24856388	0.001162021
CNR1	Cannabinoid Receptor 1	+10.16169224	8.24E-06
KRT16	Keratin 16	+9.869654755	1.51E-08
SERPINB2	Serpin Family B Member 2	-10.68741351	8.65E-14
DIPK2B	Divergent Protein Kinase Domain 2B	-10.75296482	1.61E-16
KCNN2	Potassium Calcium-Activated Channel Subfamily N Member 2	-10.82659468	2.27E-14
TNFSF18	TNF Superfamily Member 18	-10.86332649	1.11E-15
LINC01705	Long Intergenic Non-Protein Coding RNA 1705	-10.86868553	1.61E-17
CXCL1	C-X-C Motif Chemokine Ligand 1	-10.93201819	1.80E-16
LAMA1	Laminin Subunit Alpha 1	-10.97440249	7.51E-23
DMKN	Dermokine	-11.07939671	0.001933422
CXCR4	C-X-C Motif Chemokine Receptor 4	-11.14078522	9.85E-08
IL1B	Interleukin 1 Beta	-12.00296383	3.78E-05
MMRN1	Multimerin 1	-12.04519567	2.46E-11
MMP10	Matrix Metallopeptidase 10	-12.25297202	1.71E-05
ESM1	Endothelial Cell Specific Molecule 1	-12.61206416	8.49E-05
CRHBP	Corticotropin Releasing Hormone Binding Protein	-13.81052195	4.00E-12
MMP1	Matrix Metallopeptidase 1	-16.96428738	2.35E-08

Legend
CASMC: coronary artery SMC
DPC: PD0325901-differentiated PC
Log2FC: log2 fold change

Supplemental Table 3 - Differentially expressed genes relative to vascular smooth muscle contraction emerged from the contrast DPC vs. PC.

Gene	Encoded protein	Log2FC DPC vs PC	*p*-Value
MYH11	Myosin heavy chain, smooth muscle isoform	+12.71921042	8.24E-64
ELN	Elastin	+11.0093795	1.26E-146
KCNMB1	Calcium-activated potassium channel subunit beta-1	+9.539404041	3.77E-13
DES	Desmin	+9.371328427	1.07E-24
GUCY1A2	Guanylate cyclase soluble subunit alpha-2	+6.555865027	8.34E-06
ADRA2C	Alpha-2C adrenergic receptor	+5.906057628	3.70E-16
MRVI1	Inositol 1,4,5-triphosphate receptor associated 1	+5.468979277	2.38E-56
CACNA1C	Voltage-dependent L-type calcium channel subunit alpha-1C	+5.005524647	0.000991929
NPR1	Regulatory protein NPR1	+4.727254331	3.10E-15
RAMP1	Receptor activity-modifying protein 1	+4.535942973	2.48E-18
ACTA2	Actin, smooth muscle	+4.528986882	2.20E-08
CNN1	Calponin, smooth muscle	+4.44397214	7.53E-07
LMOD1	Leiomodin-1	+4.386479124	1.05E-28
HSPB6	Heat shock protein beta-6	+3.735747968	7.35E-36
MYLK	Myosin light chain kinase, smooth muscle	+3.347110572	6.77E-05
SYNPO2	Synaptopodin-2	+3.313820191	0.000935787
ITGA1	Integrin alpha-1	+2.866169072	3.26E-23
EDNRB	Endothelin receptor type B	+2.777922766	1.62E-07
EDNRA	Endothelin receptor type A	+2.699588749	6.50E-11
PPP1R12B	Protein phosphatase 1 regulatory subunit 12B	+2.663698574	5.04E-19
PPP1R14A	Protein phosphatase 1 regulatory subunit 14A	+2.652969262	2.18E-22
TAGLN	Transgelin-2	+1.773055801	1.97E-09

Legend
PC: cardiac pericytes
DPC: PD0325901-differentiated PC
Log2FC: log2 fold change

Supplemental Table 4 - Differentially expressed genes relative to angiogenesis emerged from the contrasts DPC vs. PC.

Gene	Encoded protein	Log2FC DPC vs PC	*p*-Value
LEP	Leptin	+7.55730856	5.51E-06
LYVE1	Lymphatic vessel endothelial hyaluronic acid receptor 1	+7.264998946	3.39E-08
PDGFB	Platelet-derived growth factor subunit B	+3.135408101	5.45E-21
THBS2	Thrombospondin-2	+2.216547256	9.01E-15
FBLN5	Fibulin-5	+2.069546776	4.79E-21
COL4A1	Collagen alpha-1(IV) chain	+1.868858066	3.62E-15
ANGPTL1	Angiopoietin-related protein 1	+1.555426144	0.000433708
COL4A2	Collagen alpha-2(IV) chain	+1.546143251	2.44E-10
MMP2	Matrix metalloproteinase-2	-1.508320265	1.07E-09
MDK	Midkine	-1.51698201	0.00965503
TYMP	Thymidine phosphorylase	-1.542275733	0.002290804
PDGFRA	Platelet-derived growth factor receptor alpha	-1.647252614	3.35E-06
KDR	Vascular endothelial growth factor receptor	-1.85481061	0.00030415
S1PR1	Sphingosine 1-phosphate receptor 1	-2.104668468	4.95E-05
CDH5	Cadherin-5	-2.519919527	3.14E-05
EPHB2	Ephrin type-B receptor 2	-3.079482476	1.40E-12
CXCL12	Stromal cell-derived factor 1	-3.147803764	1.16E-05
ANGPTL4	Angiopoietin-related protein 4	-3.164639029	2.95E-17
SERPINF1	Serpin Family F Member 1	-3.422907759	3.22E-30
TIE1	Tyrosine-protein kinase receptor Tie-1	-4.336370196	3.33E-06
ANGPT2	Angiopoietin-2	-4.893596067	5.27E-16
FOXC2	Forkhead box protein C2	-5.031813621	0.000398193

Legend
PC: cardiac pericytes
DPC: PD0325901-differentiated PC
Log2FC: log2 fold change

Supplemental Table 5 - Unique genes expressed by PC and DPC.

Gene (PC)	Encoded protein	Description	Localization	Human-Mouse protein identity
CADM3	**Cell adhesion molecule 3**	Calcium-independent cell-cell adhesion protein	Cell membrane	94.47%
AREG	**Amphiregulin**	Ligand of the EGF receptor/EGFR	Secreted and RE	68.6%
ACKR1	**Atypical chemokine receptor 1**	Glycosylated membrane protein and non-specific receptor for several chemokines	Cell membrane and endosomes	59.5%
SERPINA5	**Serpin family A member 5**	Glycoprotein that inhibits several serine proteases	Cell membrane and secreted	62.89%
EREG	**Epiregulin**	Secreted peptide hormone and member of the epidermal growth factor (EGF) family of proteins. Ligand of the EGFR	Cell membrane and secreted	77.5%
ALOX15B	**Arachidonate 15-lipoxygenase type B**	Member of the lipoxygenase family of structurally related nonheme iron dioxygenases involved in the production of fatty acid hydroperoxides	Nucleus, cytosol, cell membrane, and cytoskeleton	77.99%
**Gene (DPC)**	**Encoded protein**	**Description**	**Localisation**	**Human-Mouse protein identity**
CRABP2	**Cellular retinoic acid binding protein 2**	Cytosol-to-nuclear shuttling protein, which facilitates RA binding to its cognate receptor complex and transfer to the nucleus	Cytosol, nucleus, RE, and secreted	93.48%
ACTC1	**Actin alpha cardiac muscle 1**	Alpha actins are found in muscle tissues and are a major constituent of the contractile apparatus	Cytosol and cytoskeleton	100%
RCAN2	**Regulator of calcineurin 2 / Calcipressin-2**	These proteins bind to the catalytic domain of calcineurin A, inhibiting calcineurin-mediated nuclear translocation of the transcription factor NFATC1. It may also play a role in endothelial cell function and angiogenesis	Cytosol, nucleus, and mitochondria	97.46%
KRT14	**Keratin 14**	Type I keratin, usually found as a heterotetramer with two keratin 5 molecules, a type II keratin.	Cytosol, nucleus, cytoskeleton, and extracellular	88.75%
AQP1	**Aquaporin 1 (Colton blood group)**	Small integral membrane protein with six bilayer spanning domains that functions as a water channel protein. This protein permits passive transport of water along an osmotic gradient	Cell membrane, nucleus, and extracellular	94%
PI16	**Peptidase inhibitor 16**	Inhibits cardiomyocyte growth	Secreted, and RE	59.4%
PTPRO	**Protein Tyrosine Phosphatase Receptor Type O**	R3 subtype family of receptor-type protein tyrosine phosphatases. Localized to the apical surface of polarized cells and may have tissue-specific functions through activation of Src family kinases	Cell membrane, nucleus, and extracellular	90.86%
NKD1	**NKD Inhibitor Of WNT Signalling Pathway 1**	Cell autonomous antagonist of the canonical Wnt signalling pathway	Cell membrane, and nucleus	87%

JPH2	**Junctophilin 2**	Component of junctional complexes between the plasma membrane and endoplasmic/sarcoplasmic reticulum. Common feature of all excitable cell types. Mediates cross talk between cell surface and intracellular ion channels	Cytosol, nucleus, RE, and cell membrane	85.69%
TNNT2	**Troponin T2, Cardiac Type**	Tropomyosin-binding subunit of the troponin complex, which regulates muscle contraction in response to alterations in intracellular calcium ion concentration	Cytosol and cytoskeleton	83.76%

List of top genes unique to PC or DPC. The antigens later used for histological analyses of the mouse hearts were selected according to the following criteria:

- Intracellular/membrane marker suitable for microscopy imaging;
- Exclusion of soluble factors because unsuitable to localise in PC in situ;
- High identity between the human and mouse proteins;
- Availability of antibodies for histology.

Legend
PC: cardiac pericytes
DPC: PD0325901-differentiated PC
RE: rough endoplasmic reticulum

Supplemental Table 6 - Echocardiography indices in healthy mice (In vivo study 2).

	**Vehicle**		**PD0325901**	
	Basal	***Final***	***Basal***	***Final***
**E/A ratio**	1.44 ± 0.11	1.73 ± 0.19	1.53 ± 0.11	1.41 ± 0.06
**Heart rate (bpm)**	441 ± 4	454 ± 4	442 ± 3	440 ± 5
**Cardiac Output (mL/min)**	12.7 ± 1.1	13.8 ± 0.9	13.5 ± 1.0	11.6 ± 0.9
**LV Ejection Fraction (%)**	67.9 ± 5.3	74.5 ± 2.7	69.2 ± 2.4	63.8 ± 3.0
**Fractional Shortening (%)**	37.8 ± 4.3	42.6 ± 2.6	38.1 ± 1.9	33.8 ± 2.2
**Stroke volume (mL)**	27.8 ± 2.5	30.8 ± 2.3	29.9 ± 2.1	24.8 ± 2.3
**End Systolic Volume (µL)**	15.9 ± 3.2	11.7 ± 0.5	14.7 ± 1.7	14.5 ± 2.4
**End Diastolic Volume (µL)**	41.1 ± 3.9	41.1 ± 1.6	47.5 ± 2.4	39.6 ± 4.2
**LV ant wall thickness (µm)**	0.72 ± 0.06	0.66 ± 0.02	0.80 ± 0.07	0.68 ± 0.06

Echocardiography indices in healthy mice given vehicle or PD0325901. Echocardiography measurements were performed at baseline and after 14 days of treatment with vehicle or PD0325901. *N*=6/group. Values are means and SEM. *Statistics*: Two-way ANOVA was used to compare the effect of treatment and time and possible interaction. The two groups were balanced at baseline for all the measured parameters, except for CO and SV which required the use of analysis of covariance (ANCOVA). No significant differences attributable to treatment were observed.

Supplemental Table 7 - Echocardiography indices in mice with MI (In vivo study 3).

	Vehicle		PD0325901	
	Basal	***Final***	***Basal***	***Final***
**Heart rate (bpm)**	467 ± 31	531 ± 20	448 ± 34	543 ± 21
**End Systolic Volume (µL)**	15 ± 3	121 ± 32	16 ± 5	94 ± 13*
**End Diastolic Volume (µL)**	62 ± 14	152 ± 26	54.3 ± 9.6	142 ± 17
**LV ant wall thickness systole (µm)**	1.22 ± 0.13	0.34 ± 0.05	1.26 ± 0.10	0.49 ± 0.05**
**LV ant wall thickness diastole (µm)**	0.85± 0.14	0.31 ± 0.05	0.86 ± 0.16	0.38 ± 0.05**
**LV Ejection Fraction (%)**	71.6 ± 7.9	21.4 ± 8.3	72.6 ± 6.0	32.8 ± 9.3*
**Stroke volume (mL)**	46.9 ± 13.9	30.9 ± 8.5	38.7 ± 8.0	47.1 ± 16.2**
**Cardiac Output (mL/min)**	21.6 ± 46.1	16.4 ± 4.3	18.3 ± 3.4	25.6 ± 8.8**

Echocardiography indices in mice given vehicle or treatment after MI. Echocardiography measurements were performed at baseline and after MI induction and 14 days of treatment with vehicle or PD0325901. *N*=8-12 Vehicle group and *n*=11-12 PD group. Values are means and SD, * $P<0.05$ and ** $P<0.01$ vs. Final vehicle.

Supplemental Table 8 - List of antibodies employed for analyses in human samples (including Matrigel plugs).

			APPLICATION and DILUTION		
ANTIGEN	HOST	SUPPLIER - CATALOGUE - (CLONE)	IF (frozen)	ICC	WB
**CD34**	Sheep	R&D AF7227	1:100		
**CD34**	Mouse	Dako M7165 (QBEnd-10)	1:100	1:100	
**CD31**	Rabbit	abcam Ab28364	1:50	1:20	
**CD31**	Mouse	R&D BBA7 (9G11)	1:100		
**CD146**	Rabbit	Abcam ab75769 (EPR3208)		1:100	
**CD45**	Rat	NovusBio NB100-77417 (30-F11)	1:500		
**NG2**	Rabbit	Millipore AB5320	1:50	1:50	
**PDGFRβ**	Goat	R&D AF385	1:50	1:50	
**PDGFRα**	Mouse	Santa Cruz sc-398206 (C-9)		1:100	
**Vimentin**	Rabbit	Abcam ab92547 (EPR3776)		1:500	1:5000
**αSMA**	Mouse	Sigma Cy3-conjugated C6198 (1A4)	1:400	1:200	
**αSMA**	Mouse	Dako GA611 (1A4)		1:100	1:5000
**Calponin**	Rabbit	Abcam ab46794 (EP798Y)	1:100	1:100	1:10000
**SM-MHC**	Rabbit	Abcam ab53219	1:100	1:50	
**SM-MHC**	Rabbit	Invitrogen 702544 (17H3L51)			1:500
**Smoothelin B**	Rabbit	Santa Cruz sc-28562 (H-300)		1:100	1:500
**SM22α**	Mouse	Santa Cruz sc-271719 (C-11)		1:50	1:500
**CADM3**	Rabbit	NovusBio NBP1-88604		1:100	1:500
**AQP1**	Mouse	GeneTex GTX11023 (1/A5F6)		1:200	1:2000
**CRABP2**	Mouse	Proteintech 66468-1-Ig (1A5F3)		1:200	1:2000
**Ku80-XRCC5**	Goat	R&D AF5619	1:200		
**Elastin**	Mouse	Abcam ab77804 (10B8)			1:500
**COL1A1**	Rabbit	Invitrogen PA1-26204			1:1000
**Fibronectin**	Rabbit	Abcam ab299			1:5000
**P-ERK1/2**	Rabbit	Cell Signaling 4370 (D13.14.4E)			1:2000
**ERK1/2**	Rabbit	Cell Signaling 4695 (137F5)			1:1000
**P-EGFR**	Rabbit	Cell Signaling 3777 (D7A5)			1:1000
**EGFR**	Rabbit	Cell Signaling 4267 (D38B1)			1:1000
**P-FGFR**	Rabbit	Cell Signaling 3471			1:1000
**FGFR1**	Rabbit	Cell Signaling 9740 (D8E4)			1:1000
**P-ELK1**	Rabbit	Cell Signaling 9181			1:1000
**ELK1**	Rabbit	Cell Signaling 9182			1:1000
**P-STAT3**	Rabbit	Cell Signaling 9134			1:1000
**STAT3**	Rabbit	Cell Signaling 4904 (79D7)			1:2000
**P-AKT**	Rabbit	Cell Signaling 9271			1:1000
**AKT**	Mouse	Cell Signaling 2920 (40D4)			1:2000
**TCF21**	Rabbit	Abcam ab182134 (EPR13449)			1:1000
**β-Tubulin**	Mouse	Cell Signaling 86298 (D3U1W)			1:5000
**β-Actin**	Mouse	Sigma A5441 (AC-15)			1:5000
**GAPDH**	Mouse	Cell Signaling 97166 (D4C6R)			1:1000

Supplemental Table 9 - List of antibodies employed for analyses in mouse samples.

			APPLICATION and DILUTION		
**ANTIGEN**	**HOST**	**SUPPLIER - CATALOGUE - (CLONE)**	**IF (frozen)**	**ICC**	**WB**
**CD34**	Rat	Abcam ab8158 (MEC 14.7)	1:50	1:50	
**CD31**	Rabbit	Abcam ab28364	1:50	1:20	
**CD146**	Rabbit	Abcam ab75769 (EPR3208)		1:100	
**CD45**	Rat	NovusBio NB100-77417 (30-F11)	1:500		
**NG2**	Rabbit	Millipore AB5320		1:50	
**PDGFRβ**	Goat	R&D AF1042	1:50	1:50	
**PDGFRα**	Mouse	Santa Cruz sc-398206 (C-9)		1:100	
**Vimentin**	Rabbit	Abcam ab92547 (EPR3776)		1:500	
**Ki67**	Rabbit	Abcam ab16667 (SP6)		1:200	
**IB4**	-	I21414 Life Tech Biotin-conjugated	1:200		
**αSMA**	Mouse	Sigma Cy3-conjugated C6198 (1A4)	1:400	1:400	
**αSMA**	Mouse	Sigma A2547 (1A4)			1:1000
**Calponin**	Rabbit	Abcam ab46794 (EP798Y)	1:100	1:100	1:5000
**SM-MHC**	Rabbit	Abcam ab53219	1:100	1:50	
**SM-MHC**	Rabbit	Invitrogen #702544 (17H3L51)			1:500
**Smoothelin B**	Rabbit	Santa Cruz sc-28562 (H-300)		1:100	
**α-Sarc Actin**	Mouse	Sigma A2172 (5C5)	1:100		
**P-ERK1/2**	Rabbit	Cell Signaling #4370 (D13.14.4E)			1:2000
**ERK1/2**	Rabbit	Cell Signaling #4695 (137F5)			1:1000
**P-ERK1/2**	Rabbit	R&D AF1018	1:20		
**P-ERK1/2**	Rabbit	GeneTex GTX24819	1:20		
**AQP1**	Rabbit	Proteintech 20333-1-AP	1:100		
**CRABP2**	Rabbit	GeneTex GTX101551	1:100		
**S100A4**	Rabbit	Abcam ab197896 (EPR14639(2))	1:200		
**β-Tubulin**	Mouse	Cell Signaling #86298 (D3U1W)			1:1000

Supplemental Table 10 - List of TaqMan probes used for expressional analyses in human cells.

**Gene**	**TaqMan Assay ID**
ACTA2	Hs00426835_g1
CNN1	Hs00154543_m1
TAGLN	Hs01038777_g1
SMTN	Hs01022255_g1
MYH11	Hs00975796_m1
CSPG4	Hs00361541_g1
PDGFRB	Hs01019589_m1
FN1	Hs01549976_m1
COL1A1	Hs00164004_m1
ELN	Hs_00355783_m1
TCF21	Hs00162646_m1
UBC	Hs00824723_m1

Supplemental Table 11 - Congenital heart patients enrolled to the study.

Cell line	Sample type	Sample weight (g)	Surgery	Patient age (years)	Experiments performed
PC 1	RV	0.100	Pulmonary valve repair	14	*In vitro*
PC 2	RV	1.059	Pulmonary valve repair	15	*In vitro*
PC 3	RA	0.190	Atrial septal defect repair	54	*In vitro, RNA-Seq, In vivo*
PC 4	RA	0.085	Atrial septal defect repair	17	*In vitro, RNA-Seq, In vivo*
PC 5	RV	0.120	Tricuspid valve replacement + Pulmonary valve repair	23	*In vitro, RNA-Seq, In vivo*
PC 6	RV	2.700	RV outflow tract resection Previous pulmonary valvotomy within the TOF spectrum	56	*In vitro, In vivo*
PC 7	RV	0.936	Bicuspid aortic valve undergoing the Ross procedure	19	*In vitro*

Legend:
RV	right ventricle
RA	right atrium

Online extended methods

Where not otherwise stated, all chemicals were purchased from Sigma-Aldrich.

Ethics for human and animal studies

This study complies with the ethical guidelines of the Declaration of Helsinki. Human myocardial samples (right ventricle or atrium) were discarded material from surgical repair of congenital heart defects (ethical approval number 15/LO/1064 from the North Somerset and South Bristol Research Ethics Committee). Adult patients and pediatric patients' custodians gave informed written consent. Donors and samples characteristics (*n*=7) are described in **Supplemental Table 11**.
Procedures involving animals were performed under ethical licenses from the British Home Office (PPL numbers 30/3373, PP1377882, and PFF7D0506) and in compliance with the EU Directive 2010/63/EU, and following approval from Animal Ethics Committee at the University of Otago (AEC10/14), New Zealand. Procedures were carried out according to the principles stated in the Guide for the Care and Use of Laboratory Animals (The Institute of Laboratory Animal Resources, 1996).
Results are reported following the guidelines contained in the Animal Research Report of In Vivo Experiments (ARRIVE).

Studies on human and mouse cells

Isolation and primary cultures of cardiac pericytes (PC)

Human pericytes: Myocardial samples were collected in sterile, cold DMEM (Gibco) and processed within 24 h. The cell extraction protocol was as described previously (1). Briefly, samples were finely minced using scissors and scalpel until nearly homogenous and digested with Liberase enzyme (Roche) for up to 1 hour at 37 C, with gentle rotation. The digest was passed through 70-, 40- and 30-µm strainers. Finally, the cells were recovered and sorted using anti-human CD31 and anti-human CD34 microbeads (Miltenyi) to deplete the population of CD31+ endothelial cells and select CD31- CD34+ cells (these are the PC). Cardiac PC were cultured onto plastic plates with full Endothelial Cell Growth Medium 2 (ECGM2, PromoCell) including recombinant human EGF (5 ng/mL), bFGF (10 ng/mL), VEGF (0.5 ng/mL), R3-IGF1 (5 ng/mL), and 2% v/v FBS. Cells were fed every three days and passaged at 90% confluence. Optimal seeding density was 8,000 cells/cm^2. Frozen stocks of cells were preserved with 90% v/v FBS + 10% v/v DMSO. Centrifugation speed and time were 300g x 6 min. Unless otherwise stated, all experiments were performed between passage 4 and 7.

Mouse pericytes: C57BL/6 mice (*n*=5) were humanely killed under schedule 1 procedure and the heart harvested for cell extraction. Each heart was processed separately. Briefly, samples were finely minced using scissors and scalpel until nearly homogenous and digested with Liberase enzyme for up to 1 hour at 37 C, with gentle rotation. The digest was passed through 70-, 40- and 30-µm strainers. Final cells were recovered and sorted using anti-mouse CD31 microbeads (Miltenyi) and anti-mouse biotinylated-CD34 (Invitrogen) followed by anti-biotin microbeads (Miltenyi) to deplete CD31+ endothelial cells and select CD31- CD34+ cells (these are the PC). Cardiac PC were cultured with basal ECBM2 medium (Promocell) supplemented with 10% v/v FBS, recombinant mouse EGF (5 ng/mL) and bFGF (10 ng/mL) (both from Peprotech) and Amphotericin B (Gibco, final concentration of 0.25 µg/mL), onto plastic plates pre-coated with collagen from bovine skin (final concentration of 30 µg/mL). Cells were fed every 3 days and passaged at 90% confluence. Optimal seeding density was 20,000 cells/cm^2. Frozen stocks of cells were preserved with 90% v/v FBS + 10% v/v DMSO. Centrifugation speed and time were 300g x 5 min. Unless otherwise stated, all experiments were performed between passage 4 and 7.

Culture of other human cells

Cardiac fibroblasts were purchased from PromoCell and Lonza, and expanded in complete Fibroblast Growth Medium 2 (PromoCell). Coronary artery EC (CAEC) were purchased from PromoCell and

expanded in full Endothelial Cell Growth MicroVascular medium 2 (ECGMV2, PromoCell) according to manufacturer guidelines. Coronary artery smooth muscle cells (CASMC) were purchased from PromoCell and cultured with the Smooth Muscle Cell Growth Medium 2 (SMCGM2, PromoCell) according to the vendor instructions. All cells were used between passage 4 and 6.

Mycoplasma testing

All human cells used in this study tested negative for mycoplasma contamination (assessed using the PCR Mycoplasma Test Kit I/C, PromoCell, cat# PK-CA91-1096).

In vitro cellular assays

Immunocytochemistry analysis of human and mouse cells

Cells were rinsed with PBS and fixed with 4% w/v PFA in PBS for 15 min at 20°C. After washing with PBS, the cells were permeabilized with 0.1% v/v Triton-X100 in PBS for 10 min at 20°C as required. Cells were blocked with 10% v/v FBS and incubated with the antibodies as reported in **Supplemental tables 8 and 9**, for 16 h at 4°C. Secondary antibodies (conjugated with Alexa 488, Alexa 568, Alexa 647) were all purchased from ThermoFisher Scientific and used at a dilution of 1:200, for 1 h at 20°C, in the dark. Nuclei were counterstained using Hoechst (1:10,000 in PBS, 3 min at 20°C). Cells were mounted using Fluoromount-G medium (ThermoFisher).

2D-Matrigel *in vitro* angiogenesis assay

CAEC were seeded on the top of Matrigel (Corning® Matrigel® Growth Factor Reduced Basement Membrane Matrix, cat# 356231) either alone (4,000 cells/well) or in co-culture with PC (4,000 CAEC + 1,500 PC /well), using Angiogenesis µ-Slides (IBIDI, UK). Images were snapped after 5 hours, and the total tube length per imaging field was measured. To assess the interaction between PC and CAEC, PC were labelled with the red fluorescent tracker *Vybrant™ DiI Cell-Labeling Solution* (Invitrogen) according to manufacturer instructions.

In experiments with only PC (naïve or differentiated using PD0325901), 4,000 cells/well were seeded on the top of Matrigel.

In experiments with only CAEC preconditioned with PD0325901 or vehicle for 5 days, 6,000 cells/well were seeded on the top of Matrigel.

Differentiation of human and mouse pericytes into vascular smooth muscle cells (VSMC) based on GF

For experiments aiming to assess the role of the different GF in the differentiation of PC, species-specific GF were added to, or removed from, the culture medium as indicated in the single experiments. While the concentration of FBS was always kept constant. Cells were cultured for a continuous period of 10 days with full media replacement every 48 h.

Differentiation of human CASMC based on GF

CASMC grow in SMCGM2 medium, which contains recombinant human EGF and bFGF and 5% v/v FBS. For differentiation experiments, EGF and bFGF were depleted from the medium and the FBS concentration reduced to 2% (equal to experiments with PC). Cells were cultured for a continuous period of 10 days with full media replacement every 48h.

Pilot studies to determine PD0325901 efficacy and cytotoxicity

PD0325901 (Sigma-Aldrich) was reconstituted with DMSO to get a final stock concentration of 500 µM. The compound was diluted in the culture medium as required, and the same amount of DMSO used as vehicle control. Human PC were cultured for either three or ten days with increasing concentrations of the drug (125 nM, 250 nM, 500 nM, 1 µM, 2 µM). To determine the cell viability, either the live cells Calcein-AM/EthDIII staining (Biotium) or Caspase Glo 3/7 assay (Promega) were used. For this latter, the relative luminescence units (RLU) were normalized against the total intracellular protein content. Based on these findings, we determined the maximum, non-toxic dose usable for the *in vitro* and *in vivo* cell transplantation experiments. Also, Western blotting analysis of ERK1/2 phosphorylation was used to determine the minimum dose of the compound that effectively prevented ERK1/2 activation during stimulation with GF.

Differentiation of human and mouse cells into VSMC with PD0325901

Cells were cultured with media supplemented with either 250 nM PD0325901 or the same volume of DMSO (vehicle) for ten days, with complete medium exchange every 48 h.

Role played by the cell cycle arrest in human PC differentiation

To investigate whether the PC differentiation was a consequence of the cell cycle arrest, PC were cultured with full GF medium supplemented with either 1 or 10 µM Ribociclic (TOCRIS) for ten days, with complete medium exchange every other day. The same volume of DMSO was used as control. The effective inhibition of cell proliferation was confirmed using an EdU assay (below). EdU was added to the cells during say 9 and 10 of the protocol. Expression of VSMC markers was assessed using immunocytochemistry.

Treatment of human cardiac PC with the PI3K inhibitor LY294002

To investigate if the AKT signaling impacted on PC differentiation, PC were cultured with full GF medium supplemented with LY294002 (FOCUS BIOMOLECULES), a PI3K inhibitor. Three concentrations were tested: 1, 5 or 10 µM (TOCRIS). Cells were cultured for ten days, with complete medium exchange every other day. The same volume of DMSO was used as control. The effective inhibition of AKT phosphorylation was confirmed using Western blotting (described below, see signaling studies). Expression of VSMC markers was assessed using immunocytochemistry.

EdU proliferation assay

The Click-iT EdU Cell Proliferation Kit for imaging (C10337 - ThermoFisher Scientific) was used to assess cell proliferation. Cells were incubated with EdU for 24 or 48 h and then analyzed. Fluorescence staining for EdU was performed according to the manufacturer instructions.

Contraction assay

A Cell Contraction Assay (CBA-201, Cell Biolabs) was used to assess the capacity of PC to contract upon stimulation with vasoconstrictors. At the end of the 10-days differentiation protocol, differentiated and control PC were embedded in collagen gels following the manufacturer instructions. Endothelin-1 (ET1) was used at a concentration of 0.1 µM to stimulate cell contraction. Butanedione Monoxime (BDM) - contraction inhibitor provided in the kit - was added to the culture medium at a 1:100 dilution. Gels were released and the area of the gel measured at baseline and after 24h. Results are expressed as percentage of gel contraction (measuring the gel area).

Intracellular calcium flux assay

The Fluo-4 AM fluorescent calcium indicator (ThermoFisher Scientific) was employed to study the intracellular calcium flux in naïve PC and differentiated PC following stimulation with Endothelin-1. Cell media was replaced with a buffer made of 20 mM HEPES, 137 mM NaCl, 5 mM KCl, 2 mM $MgCl_2$, 1.8 mM $CaCl_2$, 5.6 mM Glucose, 1 mg/mL bovine serum albumin, 0.5 mM NaH_2PO_4 and 1 mM $NaHCO_3$, final pH = 7.4. The Fluo-4 dye (2 µM) was loaded into the cells for 15 min. HOECHST was used to counterstain the nuclei (1:1000 dilution). At the end of this incubation time, cells were stimulated with 0.1 µM ET-1 or vehicle (dH_20). Images were recorded every 20 sec for 10 min using an INCell Analyzer 2200 microscope (GE Healthcare) equipped with a 20 X objective. ET1 was introduced in the cellular system after 120 sec using a built-in injection needle. The intracellular calcium was measured as relative fluorescence units (RFU). Data analysis was carried out using the dedicated software In Cell Analyser Workstation 3.7.

Gap closure migration assay

The Radius™ 96-Well Cell Migration Assay (CBA-126, Cell Biolabs) was used to assess the migratory properties of cardiac PC and CASMC at baseline and after differentiation. Stimuli used to induce cell migration were recombinant human VEGF-A (10 ng/mL), human SDF1α (100 ng/mL) and human PDGF-BB (20 ng/mL) (all from Peprotech). Migration time was 24h, in the presence of an inhibitor of cells proliferation (hydroxyurea, 2 mM). Absence of stimuli served as a control for migration. At the end of the protocol, cells were fixed with PFA and stained for VSMC markers (α-SMA or Calponin) and HOECHST (1:10,000 in PBS). The area of the gap not covered by cells was measured.

Experiments with cardiac fibroblasts

Fibroblasts were cultured in full GF medium and preconditioned for 7 days with 250 nM PD0325901 or vehicle. For the wound closure assay, 15 to 20,000 cells/well were seeded in 96-well plates. After 24 h, a scratch was created in the confluent cell monolayer using a 20 µL micropipette tip. Cells were washed twice with PBS and incubated for 24 h using basal medium depleted of GF and FBS as control, and full GF + FBS medium as stimulus for migration. Media were supplemented with 2 mM hydroxyurea to stop cell proliferation. Images were snapped at baseline and after 24 h. The surface of wound closed was calculated. For phenotype characterization, fibroblasts were used for immunocytochemistry and Western blotting after the 7-day treatment.

Molecular analyses

Enzyme-linked immunosorbent assay (ELISA)

For measurement of GF in cell-conditioned medium, confluent cells were cultured for 48 h with basal media without FBS and GF. Media were then collected, centrifuged for 10 min at 5,000g, aliquoted and stored at -80 C until used in the experiments. Quantikine ELISA Kit anti-human VEGF, ANGPT-1, ANGPT-2 and HGF were purchased from R&D Systems. The amount of secreted factors was normalized against the total intracellular protein content. For experiments with PD0325901-differentiated PC, the conditioned medium was collected at the end of the 10-day differentiation protocol. Analytes included also LEP and SERPINF1 (R&D). For all experiments, conditioned media were diluted as required for OD readings to fall within the standard curve.

Cardiac troponin I was measured in mouse plasma using the Mouse cTn-I ELISA kit from Elabscience (E-EL-M1203), according to manufacturer instructions.

Western blotting on human and mouse whole cell lysates

Whole-cell protein lysates were collected using RIPA buffer supplemented with 1:50 proteases inhibitors cocktail and 1:100 phosphatases inhibitors. Protein lysates were centrifuged at 10,000 g, at 4°C, for 15 min. After the assessment of protein concentration (BCA Protein Assay Kit, ThermoFisher Scientific), the supernatants were aliquoted and kept at -80°C. Equal amounts of protein samples (10 to 30 µg as required) were prepared in Laemmli loading buffer, incubated for 8 min at 98°C, resolved on 8-12% SDS-PAGE and transferred onto 0.2 µm PVDF membranes (all from Bio-Rad). Membranes were blocked using 5% w/v BSA or 5% w/v non-fat dried milk (Bio-Rad) in Tris-buffered saline (TBS, BioRad) supplemented with 0.05% v/v Tween-20 for 2 h at 20°C. Primary antibodies (listed in **Supplemental Tables 8 and 9**) were incubated for 16 h at 4°C on a tube rocker. β-tubulin, β-Actin or GAPDH were used as a loading controls. Anti-rabbit IgG or anti-mouse IgG HRP-conjugated antibodies were employed as secondary antibodies (both 1:5000, GE Healthcare). Membrane development was performed by an enhanced chemiluminescence-based detection method (ECL™ Prime Western Blotting Detection Reagent, GE Healthcare) and observed using a ChemiDoc-MP system (Bio-Rad). Proteins with similar MW were assessed on different gels. No more than one stripping procedure was performed on an individual membrane when required (RestoreTM Plus Western Blot Stripping Buffer; Thermo Fisher Scientific). Western blot data were collected using the BioRad ImageLab software and analyzed using the free ImageJ software.

Western blotting on mouse liver and heart samples

Freshly frozen liver and heart samples were homogenized in RIPA buffer supplemented with proteases and phosphatases inhibitors (as described above) using gentleMACS M tubes (Miltenyi). Tissue lysates were centrifuged at 10,000 g, at 4°C, for 15 min and processed as described above.

Signaling studies in human cardiac PC

For the study of signaling activated by specific GF, human PC were cultured for 48 h with medium depleted of all GF and FBS. Cells were then stimulated with specific combinations of GF or nothing for control, for the time described in the different experiments (1, 6, 24 h). The whole-cell protein lysates were collected for Western blotting or protein arrays. A Human Phospho-kinase array kit (R&D Systems, catalogue number ARY003B) was used for quick screening of 43 kinase phosphorylation sites. For

experiments employing small inhibitory molecules (PD0325901 and LY294002), cells were treated with the drug for 1 hour before stimulation with GF.

Gene expression analysis by real-time qPCR

Extracted total RNA was reverse-transcribed into single-stranded cDNA using a High Capacity RNA-to-cDNA Kit (ThermoFisher Scientific) according to manufacturer instructions. The RT-PCR was performed using first-strand cDNA with TaqMan Fast Universal PCR Master Mix (ThermoFisher Scientific). The assay numbers for the endogenous controls and target transcripts are listed in **Supplemental Table 10**. TaqMan primer-probes were all obtained from ThermoFisher Scientific. Quantitative PCR was performed on a QuantStudio™ 5 System (ThermoFisher). All reactions were performed in a 10 µL volume in triplicate, using 7.5 ng cDNA per reaction. The mRNA expression levels were normalized against the UBC gene and determined using the $2^{-\Delta\Delta Ct}$ method.(2) For cardiac PC, the transcriptional data were further normalized versus basal CASMC for two reasons: (i) To show the difference between naïve PC and CASMC - that means the intrinsic PC commitment to the VSMC lineage; (ii) Some transcripts in PC were not expressed at baseline (e.g. *MYH11* and *ELN* were not detected in most samples and assigned Ct = 40).

Next-generation RNA-Sequencing

A whole-transcriptome analysis was carried out in 3 human PC differentiated for 10 days using PD0325901 (DPC) and compared with the respective naïve controls (DMSO-treated PC). CASMC (n=2) were used for internal reference. Strand specific RNA-sequencing was performed starting from total RNA (GENEWIZ).

RNA-Seq was carried out on an Illumina HiSeq platform, with a 2x150bp configuration, ~20M reads per sample. Sequence reads were trimmed to remove possible adapter sequences and nucleotides with poor quality using Trimmomatic v.0.36. The trimmed reads were mapped to the Homo sapiens GRCh38 reference genome available on ENSEMBL using the STAR aligner v.2.5.2b, and BAM files generated. Unique gene hit counts were calculated by using featureCounts from the Subread package v.1.5.2. Only unique reads that fell within exon regions were counted. After extraction of gene hit counts, the gene hit counts table was used for downstream differential expression analysis. Using DESeq2, a comparison of gene expression between the groups of samples was performed. The Wald test was used to generate p-values and log2 fold changes. Genes with a false discovery rate (FDR) < 0.05 and absolute log2 fold change > 1 were called as differentially expressed genes (DEGs) for each comparison. A gene ontology (GO) analysis was performed on the statistically significant set of genes by implementing the software GeneSCF v.1.1-p2. The goa_human GO list was used to cluster the set of genes based on their biological processes and determine their statistical significance.

Extended gene annotations, hierarchical clusterings, MA-plots, pathway analyses, heatmaps were generated using the open-access bioinformatics softwares iDEP 9.1 (http://bioinformatics.sdstate.edu/idep/)(3) and Morpheus (https://software.broadinstitute.org/morpheus). Venn diagrams were generated using InteractiVenn (http://www.interactivenn.net/index.html).(4) Protein-protein interaction networks were generated using the STRING software (https://string-db.org/cgi/input?sessionId=bM1QcP9Hl6oN&input_page_show_search=on).

Discovery of genes unique to PC and DPC

For discovery of unique genes characterizing the two cell populations, we extracted from the RNA-Seq dataset a list of transcripts expressed only by either PC or DPC, and not expressed by CASMC. The top genes ranked by average transcript per million (TPM) and characterized by average TPM ≥ 200 were selected per each cell type. Genes later used for histology analysis were validated at the protein level using immunocytochemistry and Western blot.

Protocols in mice

Three independent studies were conducted in mice, all following randomized controlled protocols. Study 1 was performed by AC (animal procedures) and EA (histology) in the UK. Study 2 was performed by EA (animal procedures and histology/molecular analyses) and ACT (animal procedures) (UK). Study 3 was performed by RK and DS (New Zealand).

Study 1**: *In vivo* Matrigel plugs with human PC**

Cold Matrigel (Corning® Matrigel® Growth Factor Reduced Basement Membrane Matrix, cat# 356231) was mixed with human PC ($2x10^6$) and treated with PD0325901 (500 nM) or vehicle (DMSO). Twelve-week-old male and female C57BL6/J mice (Charles River) (n=8, equal gender distribution) were injected subcutaneously into both abdominal flanks with 400 µL Matrigel, under anesthesia induced by isoflurane inhalation.

Seven days later, the animals were sacrificed, and the Matrigel plugs were harvested, fixed in 4% PFA at 4°C overnight, immersed in 30% w/v sucrose in PBS overnight and embedded in OCT for histological analyses. 15-µm thick sections were cut for immunostainings.

Study 2**: Administration of PD0325901 to healthy mice**

Seven-week-old female C57BL6/J mice (Charles River) (n=28) were housed in individual cages, in an enriched environment within a bio-secure unit under a 12 h light/dark cycle, fed with EURodent Diet (5LF5, LabDiet) and given drinking water ad libitum. PD0325901 was given orally and voluntarily each day by inclusion of the compound in sugar-free strawberry-flavored jelly, as previously described.(5, 6)

PD0325901-jelly preparation. The jelly was prepared every 2 days, stored at 4 °C and used within 48 h to ensure the stability of the drug. Mice were conditioned to eat the jelly for 5 days (twice daily) before the start of the experimental protocol. PD0325901 was given to mice (n=14) at 10 mg/kg body weight once daily; it was dissolved in DMSO and incorporated within the jelly. The control group (n=14) received DMSO-jelly. 50 µL DMSO/drug were dissolved in a final volume of 4 mL jelly, so that the final dose of DMSO was not toxic for the animals. Mice were given jelly 8 µL/g body weight. Individual housing was necessary to observe jelly consumption.

All of the mice ate the entire jelly during the experiment; therefore, none were excluded from the study. Animals were terminated 2-to-4 hours after the last jelly administration.

- A *first cohort of mice (n=3/group)* were terminated after 5 days for collection of fresh, non-fixed hearts for Western blotting.
- A *second cohort of mice (n=5/group)* were culled after 14 days. Blood was collected with EDTA for plasma separation and analysis of circulating biomarkers. A piece of livers was harvested and freshly frozen in liquid N2 for molecular biology before PFA perfusion. Hearts were stopped in diastole using KCl, perfusion-fixed (PBS-EDTA followed by 4% PFA) and harvested. Hearts and a piece of liver were then fixed in 4% w/v PFA at 4°C overnight, immersed in 30% w/v sucrose in PBS overnight and embedded in OCT (Tissue-Tek® O.C.T. Compound, VWR) for cryopreservation and histological analyses. 5-µm thick sections were cut for immunostainings.
- A *third cohort of mice (n=6/group)* underwent echocardiography at baseline and at the end of the protocol. Cardiac dimensional and functional parameters were measured with using a high-frequency, high-resolution, 3D echocardiography system (Vevo3100, Fujifilm, VisualSonics) using an MX550D transducer, with mice under isoflurane anesthesia to maintain the heart rate at 450 bpm.

  Blood flow measurement. Myocardial blood flow was assessed in the third cohort of mice, according to previously published protocols.(7-9) Briefly, carboxylate-modified green-fluorescent microspheres (cat# F8813, Invitrogen, 0.5µm diameter) were injected in the left ventricle cavity over one minute, for a total volume of 200 µL, and flushed with 150 µL of warmed saline. Reference blood samples were obtained from the descending aorta over a time of 2 min. The left ventricle, kidneys

and reference blood were collected for subsequent recovery of microspheres and fluorescence reading. The weight of the organ post-harvest was recorded. Regional LV blood flow was calculated as the absolute blood flow in ml/min/g of tissue using the formula described earlier.(8, 9) The Kidneys were analyzed to confirm the uniform distribution of fluorescent microspheres in the systemic circulation.

***Study 3*: Administration of PD0325901 in a mouse model of myocardial infarction (MI)**

This study was conducted in eight-week-old female C57/Bl6 mice (Hercus) (*n*=24). Mice were acclimatized with sugar-free strawberry-flavored jelly (prepared as above) for five days before surgery. MI was induced by permanent ligation of the left anterior descending coronary artery (LAD) as described before.(8) In brief, following anesthesia (2,2,2 tribromo ethanol, 0.3gm/kg, i.p.) and artificial ventilation, the chest cavity was opened and, after careful dissection of the pericardium, LAD was located and permanently ligated using a 7-0 silk suture. After confirming for the absence of bleeding, chest cavity was closed in layers. Animals were allowed to recover for at least 4 hours before returning to the housing unit. Mice were monitored twice a day for the first 5 days post-surgery and thereon once every day. All the mice received analgesic and antibiotic from just before the surgery for the next 3 days. Following 3 days of recovery post-MI, mice were randomized to receive the sugar-free strawberry-flavored jelly with or without PD0325901 for next 14 days. Cardiac function was assessed using echocardiography measurement at baseline and at 14 days post-MI. At the end of the treatment period and following echocardiography, and under anesthesia, hearts were stopped in diastole using KCl, and ventricular tissue was collected following perfusion-fixation with 4% PFA. The collected tissues were used to determine the effect of PD0325901 on vascularization and fibrotic remodeling following MI as described below.

Histological procedures with human and mouse hearts

Preparation and staining of tissue sections

Human myocardial samples were fixed in 4% PFA at 4°C overnight, immersed in 30% w/v sucrose in PBS overnight and embedded in OCT for cryopreservation and histological analyses. 5-μm thick sections were cut for identification of cardiac PC *in situ*.

Mice samples were prepared as described above. Per each analysis, sections were cut from different levels of the LV and distant 100 to 500 μm from each other.

Human and mouse frozen sections were post-fixed and permeabilized with ice-cold acetone (VWR) for 5 min at -20 °C, and let air dry for 30 min. Sections were rehydrated with PBS for 10 min. For P-ERK and S100A4 staining, the protocol required heat-induced antigen retrieval with Citrate Buffer 0.01M pH=6, for 10 min at 98 °C, followed by permeabilization with Triton-X100 0.1% w/v in PBS for 5 min. Tissue sections were blocked with 5% v/v normal donkey or goat serum (as appropriate) and incubated with primary antibodies for 16h at 4°C. Antibodies are reported in the **Supplemental Tables 8 and 9**. Secondary antibodies (Alexa 488-, Alexa 568-, Alexa 647-conjugated) were all purchased from ThermoFisher Scientific and used at a dilution of 1:200, for 1h at 20°C in the dark. Slides were mounted using ProLong™ Gold Antifade Mountant with DAPI (ThermoFisher Scientific).

Special stainings:

Capillaries and arterioles: Cryosections were incubated with a mix of biotinylated isolectin B4 (Griffonia Simplicifolia Lectin I isolectin B4, Biotinylated, B-1205, Vector Laboratories) to stain endothelial cells and Cy3-conjugated monoclonal anti-alpha smooth muscle actin antibody to stain arterioles, overnight at 4°C, followed by streptavidin Alexa-488-conjugated for 1 h at 20°C.

Apoptosis: The ApopTag® Fluorescein In Situ Apoptosis Detection Kit (Millipore) was employed, following manufacturer instructions.

Cardiomyocytes cross sectional area: Alexa-488 conjugated wheat germ agglutinin (WGA) (ThermoFisher) (30 min, 1:200 dilution) was used to identify cardiomyocytes borders, and alpha-Sarcomeric actin for cardiomyocytes.

Fibrosis: To identify the fibrotic tissue in the samples from the MI study, Azan Mallory staining was performed as previously described.(8, 10)

Histological analyses of in vivo studies

Study 1 - PC-Matrigel plugs

Expression of VSMC markers by human PC: The human Ku80-XRCC5 antigen was utilized to recognize the human cells. The antibody was validated in cultured human cardiac PC (positive control) and mouse tissues (negative control). Cytoplasmatic staining ruled out cell senescence.(11, 12) Antibodies anti-αSMA, Calponin and SM-MHC were used to recognize VSMC proteins. Results are expressed as the percentage of human PC expressing the indicated VSMC marker. N=2 sections were analyzed per each sample.

Infiltration of immune/inflammatory cells: an antibody anti-CD45 was employed to assess the presence of immune cells within the plugs. Results are expressed as the number of $CD45^{pos}$ cells per square millimeter of plug area. N=2 sections were analyzed per each sample.

Study 2 and 3 - Mice heart and liver

Arteriole density: Small, large and total arterioles density was expressed as the number of arterioles per square millimeter of LV area. Arterioles were classified as small (< 20µm) and large (> 20, <50 µm) according to their lumen size. All the arterioles present in two entire sections of LV were analyzed per each mouse in study 2, while arterioles were calculated in 6 fields at X200 magnification in study 3, in the peri-infarct area and in the remote myocardium separately.

Capillary density: this was evaluated in six to ten random fields (snapped using a 10x objective) in which capillaries were transversally oriented. Per each mouse, it was expressed as the number of capillaries per square millimeter of LV myocardial tissue. This analysis was also performed in mice livers.

Total arterial area: the diameter of at least 40 arterioles was measured per each mouse, in two LV sections, and the mean diameter and luminal area calculated. The mean luminal area was then multiplied for the total number of arterioles in the LV. Finally, the obtained total arterial area was normalized against the whole LV area and expressed as percentage.

Analysis of $PDGFR\beta^{pos}$ PC around arterioles: The total number of $PDGFR\beta^{pos}$ PC around arterioles - analysed in two entire sections - was expressed as the average number of PC per arteriole.

Analysis of $PDGFR\beta^{pos}$ $AQP1/CRABP2^{pos}$ PC around arterioles: $AQP1^{pos}$ and $CRABP2^{pos}$ PC were expressed as the percentage of total $PDGFR\beta^{pos}$ PC around arterioles. All the arterioles in two entire sections of LV were analyzed per each mouse.

Cardiomyocytes cross-sectional area (CSA): the CSA was measured in cardiomyocytes in which the nucleus was centrally located within the cell. All suitable cardiomyocytes were evaluated in two entire LV sections. Data are reported as the average CSA per each mouse.

Cardiomyocyte apoptosis: Apoptotic cardiomyocytes were expressed as the number of $Tunel^{pos}$ cardiomyocytes per 100,000 cardiomyocytes. Two entire sections of the LV were analyzed.

Interstitial cells apoptosis: $Tunel^{pos}$ α-Sarc $Actin^{neg}$ interstitial cells were expressed as the number of cells per square millimeter of myocardial area.

Fibroblasts and myofibroblasts density: these were evaluated in two entire sections of the LV. Fibroblasts were recognized for positivity to S100A4, while myofibroblasts for double positivity to S100A4 and αSMA. Data were expressed as the number of fibroblasts or myofibroblasts per square millimeter of LV myocardial area.

Infarct size: Data were expressed as the percentage of LV occupied by the fibrotic scar (Azan Mallory staining). A whole LV section was analyzed per each mouse.

Microscopy equipment and post-imaging analysis

Most of the analyses were carried out on photomicrographs obtained using an Axio Observer Z1 (Zeiss) equipped with 10 X and 20 X objectives. For confocal imaging, we used a Leica SP5-II AOBS multi-laser confocal laser scanning microscope attached to a Leica DM I6000 inverted epifluorescence microscope (Leica Microsystems), equipped with a 20 X, 40 X and 63 X immersion oil objectives. Images processing

was performed using the dedicated ZEN PRO and Leica LAS X software, and Photoshop CC (Adobe). Images analyses were performed using the open-access ImageJ (NIH) software. A 3D reconstruction of multiple z-stacks acquisitions was performed during the Matrigel plug experiment.

Statistical analysis

Continuous variables are presented as means ± standard error of the mean (SEM) or standard deviation (SD) of independent samples and as individual values. The D'Agostino-Pearson and Kolmogorov–Smirnov normality tests were used to check for normal distribution when applicable. Continuous variables normally distributed were compared using the Student's t-test (two-group comparison) or one-way analysis of variance (ANOVA; for multiple group comparisons). Two-way ANOVA analyses were used to compare the mean differences between groups when appropriate. Non-parametric tests, including the Mann–Whitney U test (two-group comparison) and the Kruskal-Wallis test (multiple group comparison) were used to compare data not normally distributed. Post-hoc analyses included Tukey and Dunn tests, as appropriate. Echocardiography parameters (baseline and final assessed in the same animal) were compared using paired tests; for all other analyses, unpaired tests were applied. For in vivo studies, post-hoc analyses of outcomes were conducted according to the intention-to-treat principle. In Study 2, when baseline echo measurements were found to differ between groups, the analysis of covariance (ANCOVA) was used, as it provides the optimum statistical analysis in terms of bias, precision, and statistical power. In Study 3, where final values were missing due to the early animal mortality, we used a mixed-effects model to analyze repeated measures data with missing values. Significance was assumed when $p \leq 0.05$. Analyses were performed using GraphPad Prism 8.0 and 9.0.

References

1. Avolio E, et al. Expansion and characterization of neonatal cardiac pericytes provides a novel cellular option for tissue engineering in congenital heart disease. *J Am Heart Assoc.* 2015;4(6):e002043.
2. Livak KJ, and Schmittgen TD. Analysis of relative gene expression data using real-time quantitative PCR and the 2(-Delta Delta C(T)) Method. *Methods.* 2001;25(4):402-8.
3. Ge SX, et al. iDEP: an integrated web application for differential expression and pathway analysis of RNA-Seq data. *BMC Bioinformatics.* 2018;19(1):534.
4. Heberle H, et al. InteractiVenn: a web-based tool for the analysis of sets through Venn diagrams. *BMC Bioinformatics.* 2015;16:169.
5. El-Hoss J, et al. A Combination of rhBMP-2 (Recombinant Human Bone Morphogenetic Protein-2) and MEK (MAP Kinase/ERK Kinase) Inhibitor PD0325901 Increases Bone Formation in a Murine Model of Neurofibromatosis Type I Pseudarthrosis. *J Bone Joint Surg Am.* 2014;96(14):e117.
6. Zhang L, et al. Additive actions of the cannabinoid and neuropeptide Y systems on adiposity and lipid oxidation. *Diabetes Obes Metab.* 2010;12(7):591-603.
7. Fluorescent Microsphere Resource Center UoW, Division of Pulmonary and Critical Care Medicine. Manual for Using Fluorescent Microspheres to Measure Regional Organ Perfusion. 2015;Chapter 5 - Microsphere Measurement of Regional Organ Perfusion.
8. Katare R, et al. Transplantation of human pericyte progenitor cells improves the repair of infarcted heart through activation of an angiogenic program involving micro-RNA-132. *Circ Res.* 2011;109(8):894-906.
9. Cardinal TR, and Hoying JB. A modified fluorescent microsphere-based approach for determining resting and hyperemic blood flows in individual murine skeletal muscles. *Vascul Pharmacol.* 2007;47(1):48-56.
10. Avolio E, et al. Combined intramyocardial delivery of human pericytes and cardiac stem cells additively improves the healing of mouse infarcted hearts through stimulation of vascular and muscular repair. *Circ Res.* 2015;116(10):e81-94.
11. Allard J, et al. Immunohistochemical toolkit for tracking and quantifying xenotransplanted human stem cells. *Regen Med.* 2014;9(4):437-52.
12. Seluanov A, et al. Changes in the level and distribution of Ku proteins during cellular senescence. *DNA Repair (Amst).* 2007;6(12):1740-8.
